# Long-distance air pressure differences correlate with European rain

**DOI:** 10.1038/s41598-022-14028-w

**Published:** 2022-06-17

**Authors:** Gisela Müller-Plath, Horst-Joachim Lüdecke, Sebastian Lüning

**Affiliations:** 1 Technische Universität Berlin, Berlin, Germany; 2 University of Applied Sciences HTW, Saarbrücken, Germany; 3 Institute for Hydrography, Geoecology and Climate Sciences, Hauptstraße 47, 6315 Ägeri, Switzerland

**Keywords:** Climate sciences, Hydrology, Ocean sciences

## Abstract

Precipitation in Europe shows natural fluctuations that differ considerably between seasons and geographical regions. A number of studies have linked local or seasonal rainfall variability to various long-distance air pressure differences in north–south or west–east direction. This paper presents the first continent-wide analysis of European rainfall variability on a month-by-month and country-by-country basis. We calculated Pearson r values for unsmoothed monthly rainfall data of 39 European countries over the period 1950–2019 with five potential climatic drivers, namely the North Atlantic Oscillation (NAO), the Arctic Oscillation (AO), the North Sea Caspian Pattern (NCP), and two indices of Mediterranean Oscillation (MOI2, WeMOI). For a variety of countries and months we found substantial and statistically significant r values of up to r = 0.7 and more. The dynamic temporal-spatial evolution of the Pearson correlations was mapped out across the continent, tracking the gradual or abrupt expansion, displacement and subsequent waning of the various effects over the course of the year. The correlation analysis was complemented by best subset multiple regression, controlling for intercorrelation of the potential drivers. Our results may help to improve short- to midterm rainfall prognoses in Europe and provide important calibration data for the further refinement of climate models.

## Introduction

Rainfall in Europe is characterised by significant year-to-year decadal and multidecadal variability. Previous studies identified several oceanic modes of variability that systematically correlate regionally and seasonally with rainfall over the continent^1–6^. Nevertheless, more work is needed to transition our knowledge from mostly qualitative to fully quantitative models. In this contribution we are comparing monthly precipitation data from 39 European countries for the past seven decades with five well-known long-distance atmospheric pressure indices. For the analysis, we selected two north–south-defined indices (North Atlantic Oscillation NAO and Arctic Oscillation AO), two west–east-defined indices (North Sea Caspian Pattern NCP and Mediterranean Oscillation Index MOI2), and one southwest-northeast-defined index (West Mediterranean Oscillation Index WeMOI). For the first time, we are mapping out Pearson correlation coefficients *r* separately for all 12 months of the year and for all 39 countries. Every rainfall series is then modelled by a multiple regression, using a best subset approach including validation. The objective of the study is to identify sweet spots in Europe where the regional precipitation time series correlate best with the modes of variability and are best predicted by them. This continent-wide analysis relies exclusively on statistical correlations and does not attempt to dwell into the underlying complex physical processes. However, these statistical relationships may help to improve early seasonal to midterm rainfall prognoses in Europe and provide important calibration data for the further refinement of climate models. A better understanding of potential rainfall drivers and teleconnections is also needed to more reliably attributing extreme weather events such as floods and drought to natural or anthropogenic triggers^7–9^.

**Figure 1:**
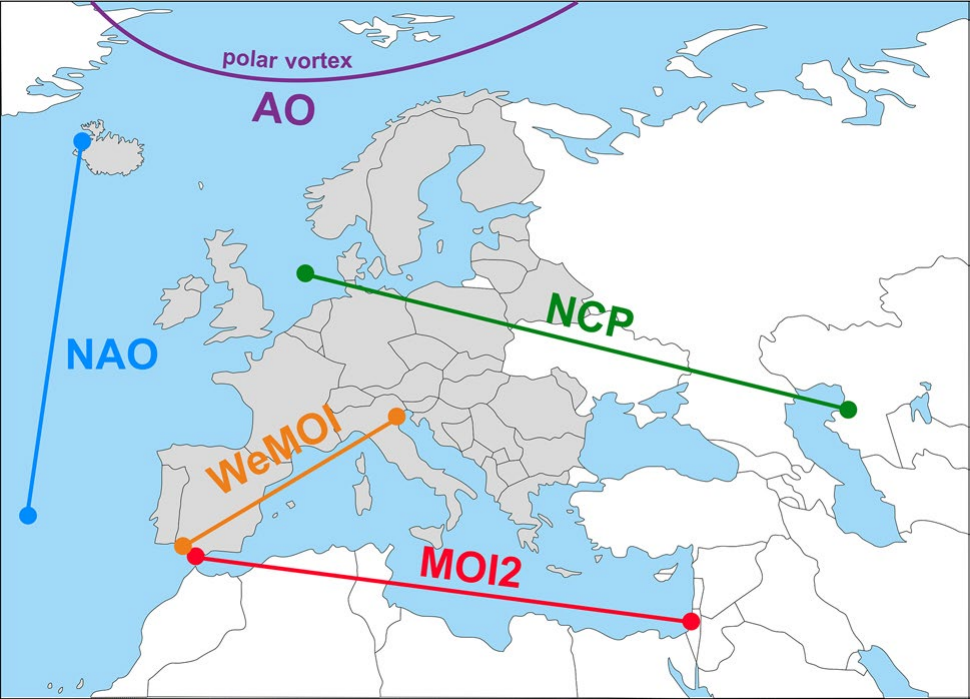
Geographical location of the precipitation area (39 European countries, coloured grey) and the five long-range air pressure indices studied in this paper. For NAO, NCP, MOI2 and WeMOI, the map shows the locations of reference points to calculate the air pressure differences. The AO is shown schematically as it is not defined by a point-to-point air pressure difference (see text). The geographical base map was retrieved from URL https://www.d-maps.com/carte.php?num_car=13180&lang=en.

**Figure 2:**
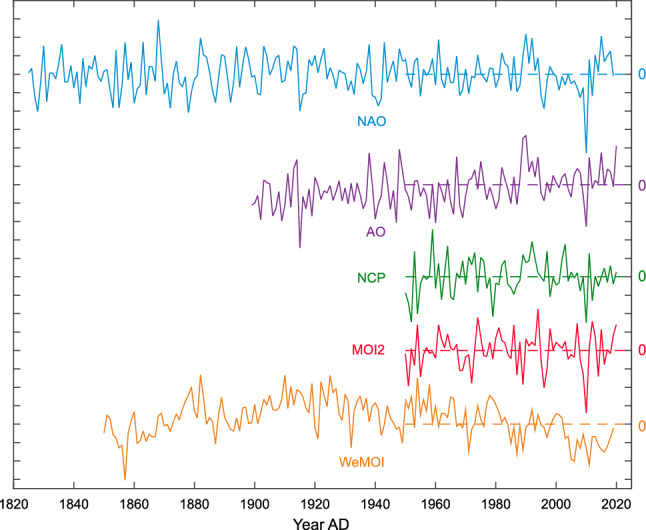
Time Series of NAO (blue), AO (purple), NCP (green), MOI2 (red), and WeMOI (orange) index over the respective available period. Shown are mean annual values with a step size of 0.5. The horizontal dashed lines mark index zero across the study period 1950–2019.

Variability of precipitation is undoubtedly caused by multiple and interacting factors, ranging from sea surface temperature (SST) variations to different types of atmospheric variability to changes in landscape features and local thermal and cloud-forming processes^10^. The present paper focuses on long-range pressure differences, so-called teleconnections, in near-surface atmospheric layers (up to 500 m height above sea level). For our comprehensive analysis, we selected five such modes that cover the studied area in Europe, are spatially sufficiently heterogeneous, and have been described in the literature as being related to precipitation, arriving at NAO, AO, NCP, MOI2 and WeMOI. Figure 1 shows their geographical location, Fig. 2 their temporal evolution.

A second issue we had to decide on was the spatial resolution of the precipitation data. Whereas grid data better characterise the physics of precipitation, country data are more concise in the presentation of results. To cover all of Europe, the two approaches require different inferential statistics and thus separate papers because of the large number of grid cells. Having chosen the country analysis here, we already provide some results for grid cells in the supplement for comparison and outlook.

### North Atlantic Oscillation (NAO)

The NAO index is based on the surface sea-level pressure difference between the subtropical Azores High and the subpolar Icelandic Low^11^. During the positive NAO index phase, the Azores High is stronger and the Icelandic Low lower, resulting in a greater pressure difference. Westerlies are increased and the North Atlantic jet stream and storm track takes a more northerly route. Positive NAO conditions are typically associated with cool summers and mild and wet winters in the British Isles and Scandinavia^12–14^. In contrast, the negative NAO index phase shows a weak subtropical high and a weak Icelandic low, leading to a reduced pressure gradient, suppressed westerlies and a shift of the North Atlantic jet stream and storm track southwards toward the Mediterranean Sea. Northern European winters during negative NAO conditions are colder and drier than usual, whilst southern Europe and North Africa receive more rainfall and storms^9,12,14,15^. NAO and AO share many similarities, but also exhibit some key differences, justifying separate analyses in this study^16–18^.

### Arctic Oscillation (AO)

The Arctic Oscillation or Northern Annular Mode/Northern Hemisphere Annular Mode (NAM) is defined by the strength of atmospheric pressure over the Arctic^19^. During the positive AO index phase, the Arctic air pressure is lower than average and the jet stream farther north. Strong westerly winds circulating counter clockwise around the Arctic confine the cold polar air, resulting in fewer cold air outbreaks to mid latitude regions. During the AO's negative index phase, atmospheric pressures over the Arctic region are higher than average, shifting the jet stream towards the equator. The winds circulating around the Arctic are weaker, leading to more frequent outbreaks of frigid polar air to mid latitudes in winter. The north–south shift in the westerlies affects precipitation in Europe. Positive AO conditions are typically associated with higher precipitation in northern Europe and drought in the Mediterranean region, whilst negative AO conditions result in enhanced southern European and Mediterranean winter rainfall^20,21^.

### North Sea Caspian Pattern (NCP)

The NCP refers to an atmospheric teleconnection between the North Sea and North Caspian at the 500 hPa geopotential height level^22^. NCP correlations with rainfall in the Balkans, Turkey and Israel are reported as complex^23^. At the Turkish Aegean coast, rainfall correlates negatively in winter and positively in summer^24^. Other authors reported negative correlations of NCP and rainfall for central, western and north-western Europe^25^.

### Mediterranean Oscillation Index (MOI2)

Various versions of the MOI exist that differ in their definitions^26–29^. Significant correlations between different MOI indices and climatic parameters such as precipitation and temperature have been reported from the Mediterranean area^6,29–33^. For this paper we have selected the station-based MOI2 (or MO_GI_) which is defined as normalised pressure difference between Gibraltar and Israel, covering the entire Mediterranean Basin in west–east direction^27^.

### West Mediterranean Oscillation Index (WeMOI)

The WeMOI is an index measuring the difference between the standardized atmospheric pressure recorded at Cádiz (southwest Spain) and Padua (northern Italy)^8^. With positive WeMOI conditions, rainfall in southwest and eastern Iberia is typically reduced, whilst above-average rain occurs in central northern Iberia^8,34,35^. Precipitation anomalies reverse during negative WeMOI conditions.

## Results

### Pearson correlations between atmospheric indices and rainfall

**Figure 3:**
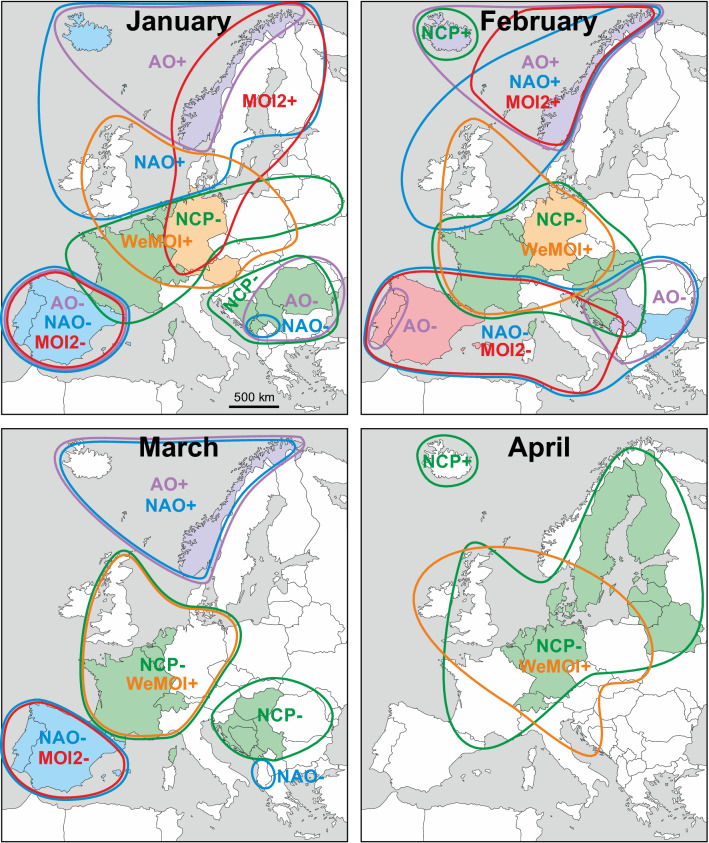

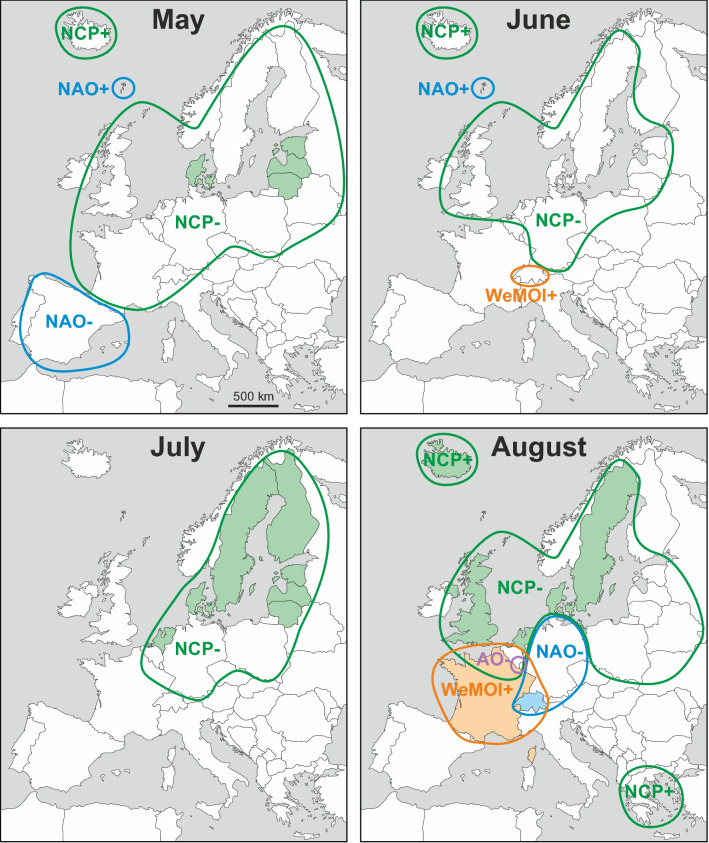

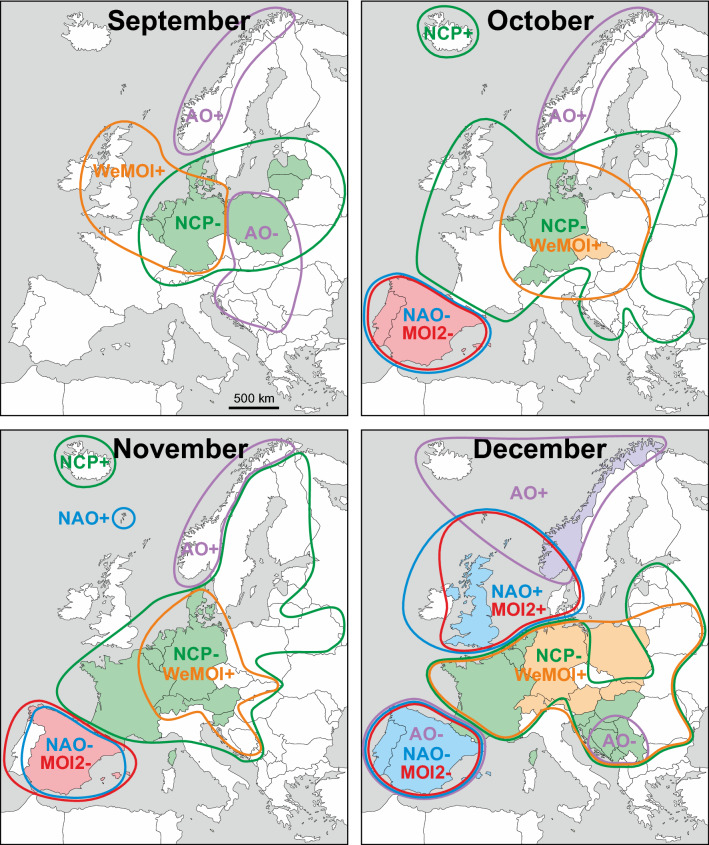
Monthly maps showing regions in which rainfall correlated with atmospheric indices with *r* ≥ 0.50/ ≤ − 0.50 (coloured framing) and *r* ≥ 0.60 / ≤ − 0.60 (coloured filling). The plus/minus signs to the right of the atmospheric indices denote positive and negative correlations with rainfall (e.g. NAO+ and NAO−, not to be confused with the positive and negative phases of the index). The colours are: NAO = blue, AO = purple, NCP = green, MOI2 = red, WeMOI = orange. The geographical base map of Europe was retrieved from https://www.d-maps.com/carte.php?num_car=2232&lang=en. Detailed correlation maps and statistical results tables are provided in the supplementary Figs. S1–S5 and Tables S1–S5.

**Figure 4:**
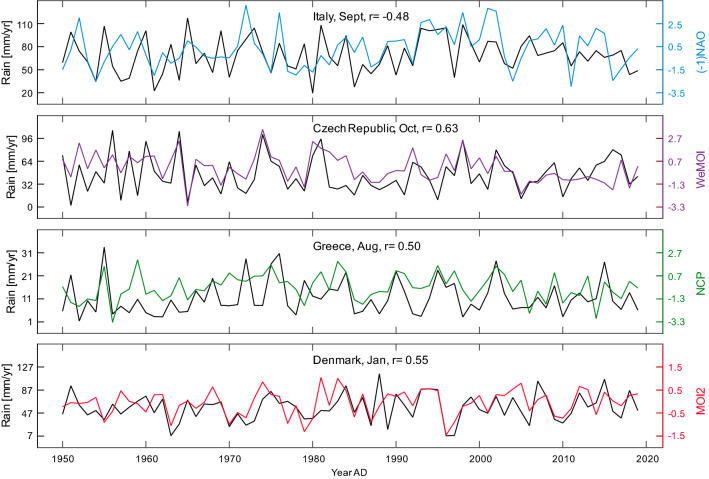
Illustration of four exemplary correlations *r* in a time series format: NAO and rain in Italy in September, WeMOI and rain in Czech in October, NCP and rain in Greece in August, and MOI2 and rain in Denmark in January. In the uppermost graph, the NAO series was mirrored at zero to facilitate comparison. More time series are provided in the Supplement (Fig. S6).

The most important relationships are summarised in Fig. 3, geographically comparing all atmospheric indices (potential drivers of rainfall) with a correlation *r* equal or larger than ± 0.5. This threshold was chosen to maintain clarity and guarantees statistical significance even after applying Bonferroni correction for multiple testing. Some interesting correlations are exemplarily illustrated in time series format in Fig. 4 and more examples in the supplementary Fig. S6. The *r* values are regionally mapped out in Figures S1-S5 using a colour code, which is based on absolute *r* values and not on statistical significance in order to allow comparison of effect sizes across countries, drivers and with the literature. Tables S1-S5 in the Supplement show all 2340 linear correlation coefficients *r* computed for 39 countries, 12 months, and 5 atmospheric indices. Seasonal differences are emphasised in the graphs in the supplementary Fig. S7. In order to compare how spatial resolution affects correlation, some exemplary results for gridded precipitation in more or less extended countries data are provided in the supplementary Tables S10–S14.

#### Winter (December–February)

Throughout the winter months, NAO, AO, and MOI2 correlate positively with precipitation in northwest (NW) Europe, and negatively in southwest (SW) Europe. In December, a small area in the Balkans at the Adriatic Sea shows negative correlations with the AO (henceforth termed AO−) which in February and March expand eastwards towards the Black Sea coast. Towards the end of winter in February, the NAO− correlations cover the entire Mediterranean area, and the MOI2− correlations reach from Iberia eastwards up to the Adriatic Sea. Large parts of central Europe are characterised by NCP− and WeMOI+ correlations during winter.

#### Spring (March–May)

In March, the Mediterranean area with NAO− and MOI2− correlations shrinks significantly compared to the winter months. NAO+ and AO+ correlations still exist in parts of NW Europe. NCP− and WeMOI+ correlations occur in parts of western, central Europe and in the Balkans. These expand and shift towards the Baltic and Scandinavian region in April and May. Iceland is characterised by NCP+ correlations. NAO, MOI2 and AO do not play a major role during April. In May, Spain shows NAO− and the Faroe Islands NAO+ correlations.

#### Summer (June–August)

The dominant relationship in summer is represented by NCP− correlations in parts of western and central Europe as well as in Scandinavia. Additional correlations are registered in August, namely WeMOI+ in France, NAO− in Germany and Switzerland, and NCP+ in Iceland and the Balkans.

#### Autumn (September–November)

The area dominated by NCP correlations shifts southwards in autumn, covering variable regions from France/British Isles to the Baltic. Some of the area shows additional overlapping WeMOI+ correlations. Throughout autumn, Norway is represented by AO+ correlations. An eastern European area stretching from Poland to the Balkans has AO− correlations during September. Iberia is characterised by NAO and MOI2 correlations in October and November, but not yet in September.

#### Gridded precipitation

Especially for spatially extended countries like Italy, it is obvious that the relationship between atmospheric indices and precipitation is not homogeneous across the entire country. The correlations in Fig. 3, mapped out for country data, suggest already that for example, in November northern Italy is characterised by NCP− whereas southern Italy is not. The correlations computed for two single 1° × 1° grid cells in northern and southern Italy in the supplementary Table S12 confirm this exactly. Similarly differential results were confirmed e.g. for WeMOI+ in northern but not southern France in September (supplementary Table S14).

### Interdependence of atmospheric indices, best subsets and validation

**Figure 5:**
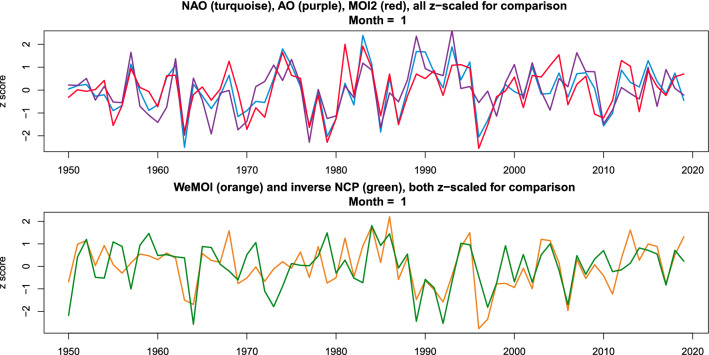
January time series of the five atmospheric indices, clustered according to the *r* values in Table 1: The upper panel shows the synchronicity of NAO, AO, and MOI2, the lower panel that of WeMOI and NCP.

In every month of the year, there was substantial intercorrelation of the atmospheric indices analysed in this paper, which is exemplarily shown in Table 1 and Fig. 5 for January, and supplementary Table S6 for all months. In general, intercorrelations weakened in summer, except for NAO-AO.

**Table 1:** Pairwise correlation (Pearson *r* values) of the January indices of the five atmospheric indices across the years 1950–2019.

	NAO	AO	NCP	MOI2	WeMOI
NAO	1	0.79***	0.18	0.83***	0.37**
AO	0.79***	1	0.38**	0.55***	0.02
NCP	0.18	0.38**	1	0.06	− 0.61***
MOI2	0.83***	0.55***	0.06	1	0.55***
WeMOI	0.37**	0.02	− 0.61***	0.55***	1

Significant values are marked with *(*p* < 0.05), **(*p* < 0.01), ***(*p* < 0.001), without correction for multiple testing.

**Figure 6:**
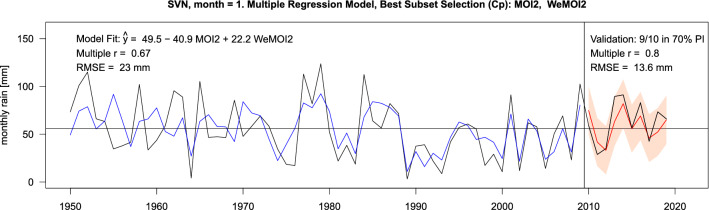
Best subset regression and validation for January rainfall in Slovenia. The black line represents the actual rainfall 1950–2019—average marked by the horizontal. In the left part of the graph (1950–2009), the blue line shows the rainfall $\widehat{Y}$ as modelled by the regression equation in row 1 of Table 2: Other atmospheric indices beside MOI2 and WeMOI were not relevant for this example. The right part illustrates the validation in the years 2010–2019 with the red line as the predicted rainfall that resulted from inserting the MOI2 and WeMOI values observed in 2010–2019 into the regression equation from 1950 to 2009. The pink area is the 70% confidence interval of prediction (see *Methods* below and *Statistical Methods* in Suppl. ch. 2.2).

The results of the best subset regression and validation are exemplarily displayed for January rainfall in the countries along the Balkan coast in Table 2 and more detailed for Slovenia in Fig. 6. Results for other European regions and months can be found in Suppl. ch. 8 (supplementary Tabs. S7–S9 and Figs. S8–S10).

**Table 2:** Best subset regression for seven countries along the Balkan coast in January, ordered north to south.

Country	$\hat{\alpha }$	$\hat{\beta }_{1}$	$\hat{\beta }_{2}$	$\hat{\beta }_{3}$	$\hat{\beta }_{4}$	$\hat{\beta }_{5}$		
	Intercept	NAO	AO	NCP	MOI2	WeMOI	r (fit)	r (val)
Slovenia	49.5	–	–	–	− 40.9	+ 22.2	0.67	0.80
Croatia	71.6	–	–	–	− 56.4	+ 29.9	0.79	0.90
Bosnia-Herz	68.9	–	–	− 8.7	− 40.1	+ 19.7	0.78	0.80
Montenegro	98.9	− 7.2	–	–	− 54.3	+ 39.5	0.81	0.83
Albania	101.4	− 9.3	–	–	− 38.2	+ 38.0	0.77	0.63
N Macedonia	46.3	− 7.7	–	–	–	+ 12.8	0.73	0.46
Greece	97.3	− 16.2	–	–	+ 22.6	+ 24.4	0.71	0.65

Shown are the estimated coefficients in the regression equation $\hat{Y} = \hat{\alpha } + \hat{\beta }_{1} X_{1} + \cdots + \hat{\beta }_{5 } X_{5}$*,* with $\hat{Y}$ denoting the modelled rainfall and $X_{1} , \ldots , X_{5}$ the five atmospheric indices, fitted to the data of the years 1950–2009 ("fit sample", see Methods). The slash indicates that the respective index $X_{j}$ was dispensable according to the best subset criterion. The last two columns show the correlation between predicted and observed rainfall in the fit sample (1950–2009) and the validation sample (2010–2019).

In our detailed example, January rainfall in Slovenia (Fig. 6), the validation proved successful according to the three criteria used (see *Methods* below and *Statistical Methods* in Suppl. ch. 2.2): The 70% confidence interval of prediction (PI), marked by the pink area, contained not only 7 out of 10 (70%) but even 9 out of 10 observed rainfall values. The correlation *r* = 0.80 between predicted and observed rainfall, and the average deviation of observed from predicted rainfall RMSE = 13.66 mm were both very good in absolute terms and did not deteriorate in the validation versus the fit sample.

Summarising Table 2, January precipitation in the northern Balkan coast countries is mainly driven by WeMOI+ and MOI2−. From Bosnia-Herzegovina southwards, additionally NAO− comes into play. In northern Macedonia, the impact of MOI2− ceases, and in Greece (which is a country that extends largely to the east and south of the Mediterranean) it even turns positive. This geographically coherent pattern of impacts of the atmospheric indices may be regarded as a further validation of the approach.

In other parts of Europe, we obtained similar successful model fits and validations according to confidence intervals of prediction, multiple correlation, RMSE, and geographical coherent patterns of impacts (further examples are shown in Suppl. ch. 8). Overall, however, results for the autumn, winter and early spring months were much better than for late spring and summer, where not for all countries a satisfactory regression model could be identified and validated. These seasonal differences can already be seen from the graphs in supplementary Fig. S7, where the *r* values for all atmospheric indices and all countries are plotted across the months of the year.

In sum, the approach of best subset regression and validation proved successful for most months of the year according to our three criteria: The proportion of observed rainfall data in 2010–2019 falling within the 70% confidence interval of prediction (PI) was 67%. Broken down by months, exactly 70% predictions were successful in the months 1–4 and 8–12 but only 61% in the months 5–7. In more than half of the series, the multiple correlation exceeded *r* = 0.6 in both the fit and validation sample, and RMSE, which can be termed prediction accuracy, was overall much less than one standard deviation of the monthly rainfall series. To further bolster our approach, we finally repeated the entire analysis for the full multiple regression model with all five possible drivers as predictors. In no case did the full model outperform the model with the best subset of predictors.

## Discussion

### Seasonal and regional trends

#### NAO

The previously described general winter dipole with rainfall correlating positively with the NAO (NAO+) in the British Isles and Scandinavia and negatively (NAO−) in southern Europe ^12–15^ is supported by the current study. Our month-by-month approach documents the systematic build-up, regional expansion and finally the waning of the cold season NAO correlations in Europe between October and May. During the majority of the summer months, however, trends in NAO (and AO) show no major similarity with rainfall. A noteworthy exception is a distinct NAO− correlation in Germany-Switzerland during August.

#### AO

The AO+ and AO− correlations reported in the literature in northern and southern Europe^20,21^ have been confirmed by our study. Due to some similarity of trends in NAO and AO, it does not surprise that correlation areas of the two modes of variability often overlap. Nevertheless, distinct differences exist. From September to December, persistent AO+ correlations occur in Norway, whilst the NAO plays no major role.

#### NCP

The NCP− correlations reported for central, western and north-western Europe^25^ were generally confirmed and specified by our study, as well as the NCP− relationship in winter turning to NCP+ in summer in the Balkans^24^. We found strong NCP correlations in every single month of the year, with the correlation area lying more southerly in winter than in summer and being largest in April/May. At the peak of summer in July, NCP is the only index that keeps a relationship with rainfall in Europe, whilst the other four studied indices lack substantial correlations. NCP correlations in Europe are typically negative. The only exceptions are Iceland and the Balkans which in some months show a NCP+ relationship.

#### MOI2

Various papers have reported MOI2− correlations for the northern Mediterranean region based on annual and monthly data^6,29–32^, a relationship that is generally supported by our study. Comparing countries and seasons, we found the strongest correlations in autumn, winter and early spring in the Iberian Peninsula, somewhat weaker and shorter lasting ones in Italy and the northern Balkans, and in the latter even a second MOI2− phase in summer, For the southern Levant, a MOI+ correlation of an Algiers vs. Cairo dipole for winter rainfall has been reported^33^, indicating possible dipole patterns with parts of the southern Mediterranean region. Notably, MOI2 correlations often overlap with NAO and AO patterns. This is particularly surprising for the Scandinavian MOI2+ correlations in January and February, given that the definition of the MOI2 parameter is based on two points in southern Europe. In January, the MOI2+ area reaches southwards into Germany where it overlaps with WeMOI+ and NCP− correlations.

#### WeMOI

This parameter was originally introduced to explain deviations in different parts of Iberia^8,34,35^. Such higher resolution within countries was not the objective of our study which focused on countrywide precipitation data. Nevertheless, WeMOI unexpectedly yields excellent correlations for western and central Europe for most months of the year. This is probably because the reference points of this index are oriented SW-NE, in contrast to the W-E direction of MOI2.

In general, for most seasons and European regions, substantial and statistically significant correlations could be established for at least some of the atmospheric indices. However, there were a few noteworthy exceptions (see e.g. the evolution of *r* values across the year in supplementary Fig. S7): None of the indices analysed here showed a significant correlation with precipitation in the Balkans in late spring or in the Iberian Peninsula in midsummer.

Comparing the results of best subset regressions to those of the Pearson correlations, we found that often but not always the atmospheric indices with high Pearson *r* values "survived" the best subset selection. For example, in Fig. 4 the indices in the upper three time series (September NAO, October WeMOI, August NCP) were confirmed as relevant predictors for the respective rainfall series (Italy, Czech, Greece), whereas in the bottom graph, the January MOI2 was identified as a by-product of the NAO (*r* = 0.83 in Table 1) and thus not relevant by itself for the rainfall in Denmark.

### Forecasting potential

In line with the various literature cited above, the correlations and multiple regressions presented in this study do not involve any time lags. Therefore, a mid-term rainfall forecast cannot be based on temporally preceding observations of the atmospheric indices. However, rainfall predictions based on the observed temporal-spatial correlation patterns become possible if the future development of the different atmospheric indices can be prognosticated: Particularly our best subset regression equations would then immediately provide a reliable forecast of rainfall for most months of the years and most European countries, as the successful validations show. For large countries with inhomogeneous precipitation, however, this can only be a first step towards a reliable forecast and needs to be complemented by spatially better resolved results, as hinted at by the preliminary results for grid cells in the supplementary Tables S10–S14).

Several groups have already developed empirical and dynamical models which allow skilful NAO forecasts from one month to more than a year ahead^36–39^. Attempts have also been made to predict the AO^40^ and WeMOI^41^. Multidecadal NAO changes have been observed for the past^42^ with 30–35 years cycles related to combined teleconnections^43^. Multidecadal NAO forecasts are linked to the Atlantic Multidecadal Oscillation (AMO) with an opposite-sign relationship between the polarities of the AMO and the NAO, whereby the AMO signal precedes the NAO by 10–15 years^44^. For long term trends on rainfall, the future development of the indices may be simulated in climate models and rainfall response deduced from the observed correlations^45^.

## Methods

### Data

The years 1950–2019 were chosen for analysis because this was the largest overlapping period for which both precipitation data and data on all five modes of variability were available. Monthly rainfall data for 39 European countries were downloaded from the Climatic Research Unit (CRU) of the University of East Anglia (dataset CRU CY v4.04 Country Averages, variable pre). The monthly NAO, MOI2, and WeMOI data were sourced from the CRU, the AO data from NOAA (1950–2019) and Colorado State University (1899–1949), and the NCP data were calculated according to the formula of Kutiel and Benaroch ^22^ based on gridded monthly geopotential heights provided by the NOAA. Web addresses, dates of access, and details of calculating NCP values are listed in Supplement ch. 2.1. All data were used without any smoothing.

### Statistical processing

#### Correlation coefficients and statistical significance testing

For every month of the year, the Pearson correlation coefficient *r* between the monthly value of each atmospheric index NAO, AO, NCP, MOI2, WeMOI and the monthly precipitation in each European country was computed across the years 1950–2019, without any smoothing of the data. In order to test whether the *r* values differed significantly from zero, we applied the *t*-test. The assumption of independence the *t*-test relies on is justified by the long-known observation that rainfall series show almost no autocorrelation^46,47^. The assumption of normality could be dispensed with because of the central limit theorem. With N = 70 years and significance level *p* < 0.05, the *t*-test yields a critical *r* of ± 0.235, i.e. with a probability of less than 5%, an empirical *r* will exceed this threshold by chance. With 12 months, 39 countries and 5 potential drivers we are well aware that most likely there will arise a certain amount of false positives among the results. For remedy, we applied a Bonferroni correction across the 12 × 39 = 468 *t*-tests for every potential driver, limiting the risk of one or more false positives arising among these to overall 5%. With Bonferroni correction, the critical *r* is ± 0.447. However, since all available correction routines for multiple tests inflate the false negative rate, and this even more the stronger the tests are interdependent (^48^, pp. 257–261), we report both the significance of *r* values with and without Bonferroni correction in the supplementary Tables S1–S5.

#### Interdependence of drivers and best subset regression

In interpreting the correlations, one has to take into account that our five atmospheric indices (potential drivers of rainfall) may be intercorrelated. In order to investigate which of them have their own effect on precipitation and which are merely the by-product of others, we statistically controlled the effect of each mode by means of multiple regression. However, a multiple regression model with all available modes as predictors, especially highly correlated ones, usually leads to "overfitting", i.e. regression coefficients that are largely random and not replicable by future data. We therefore used a "best subset" regression to individually model precipitation in every country and month. Hereby, only those predictors entered the regression equation that made a measurable contribution to rainfall prediction in the context of the others (the criterion was Mallow's Cp ^49^, ch. 13.2.2, 22.1, 22.3). For selecting and validating the relevant subset of predictors, we applied a simple and statistically honest procedure: We divided the data into two parts, using the years 1950–2009 for selecting the best subset of drivers ("fit sample") and the years 2010–2019 for validating it ("validation sample"). In the latter, precipitation was predicted with the multiple regression equation obtained from the former and then compared to observed precipitation. The correspondence of predicted and observed was assessed with the following three criteria: A confidence interval of prediction PI (^49^, p. 239), the correlation between predicted and observed values, and the root mean squared error RMSE (the average deviation of observed from predicted rainfall in millimetres). For details, see the Supplement ch. 2.2.

Statistical data analysis was carried out with MatLab version R2015a (Figs. 2, 4, supplementary Figure S6; supplementary Tables S1–S5, S10–S14) and R version 4.0.2 ^50^ (Figs. 5, 6, supplementary Figures S7–S9; Tables 1, 2, supplementary Tables S6–S9). For the map Figs. 1, 3, and the supplementary map Figures S1–S5, vectorised base maps were retrieved from https://www.d-maps.com/carte.php?num_car=13180&lang=en and https://www.d-maps.com/carte.php?num_car=2232&lang=en and modified using Corel Draw Graphics Suite X8.

## Supplementary Information

Supplementary Information.

## Data Availability

Web addresses, dates of access, and details of data processing, particularly of calculating NCP values, are listed in Supplement ch. 2.1. The datasets generated during the current study are available from the corresponding author on reasonable request.
