# Supplementary material for: Long-distance air pressure differences correlate with European rain
Source: Sci Rep. 2022 Jun 17;12:10191. doi: 10.1038/s41598-022-14028-w (PMC9206005; doi:10.1038/s41598-022-14028-w)
Supplement: Supplementary file 1 — Supplementary Information. [file 41598_2022_14028_MOESM1_ESM.pdf]

Supplement to

Long-distance air pressure differences correlate with European rain

Gisela Müller-Plath[1]*, Horst-Joachim Lüdecke[2], Sebastian Lüning[3]

[1]Technische Universität Berlin, Berlin, Germany. E-Mail: gisela.mueller-plath@tu-berlin.de

[2]University of Applied Sciences HTW, Saarbrücken, Germany. E-mail: moluedecke@t-online.de

[3]Institute for Hydrography, Geoecology and Climate Sciences, Hauptstraße 47, 6315 Ägeri, Switzerland. E-mail: luening@ifhgk.org

*Corresponding author

Contents of this file **page**

1. Overview 2

2. Data Sources and Statistical Methods **3**

2.1 Data Sources.. 3
2.2 Statistical Methods 4

3. Pearson Correlation Coefficients................... 7

Table S1: NAO vs. Precipitation 8
Table S2: AO vs. Precipitation 9
Table S3: NCP vs. Precipitation.............................. 10
Table S4: MOI2 vs. Precipitation 11
Table S5: WeMOI vs. Precipitation 12

4. Correlation Coefficient Maps........................... **13**

Figure S1: NAO vs. Precipitation 14
Figure S2: AO vs. Precipitation............................... 18
Figure S3: NCP vs. Precipitation 22
Figure S4: MOI2 vs. Precipitation 26
Figure S5: WeMOI vs. Precipitation........................ 30

5. Comparative Times Series of Best Correlations in z Score Format **34**

Figure S6: 32 graphs for largest absolute *r* values (|*r*| ≥ 0.72) 34

6. Evolution of Pearson *r* for all European Countries through the Year **41**

Figure S7 41

7. Intercorrelation of Atmospheric Indices (Potential Drivers) for all months 44

Table S6 44

8. Best Subset Regression: Fit and Validation for Exemplary Regions and Months46

Figures S8-S10 and Tables S7-S9:
Best subset regression and validation for geographically adjacent countries in three selected European areas and one month each 47

9. Exemplary Pearson Correlation Coefficients for Gridded Precipitation 50

Tables S10-S14: NAO, AO, NCP, MOI2, WeMOI vs. Precipitation 50

10. References 52

1. Overview

This Supplement contains detailed methods and results that could not be shown in the main manuscript for reasons of brevity.

Supplement Chapter 1 is represented by this overview.

Supplement Chapter 2 lists the detailed web URLs of all data sources and how the time series for this study were computed. Further, the chapter contains a detailed description of the statistical methods, which are based on linear modelling.

Supplement Chapter 3 shows the correlation coefficients and their statistical significance without and with Bonferroni correction for comparing precipitation with NAO (Tab. S1), AO (Tab. S2), NCP (Tab. S3), MOI2 (Tab S4) and WeMOI (Tab. S5).

Supplement Chapter 4 contains a complete set of Europe maps showing the Pearson *r* coefficients equal or more than ± 0.40 for all 12 months of the year arranged by season, separately for all five atmospheric indices as potential rainfall drivers.

Supplement Chapter 5 presents selected time series of monthly rainfall compared to the five atmospheric indices for the 32 best positive and negative *r* coefficients found in the study, sorted by atmospheric index and in descending order of absolute *r*. Time series are shown as anomalies divided by the standard deviation (z -score) for better comparison.

Supplement Chapter 6 shows the evolution of Pearson *r* values throughout the year from January to December in all European countries, i.e. it summarises the *r* values from Tables S1-S5 by contrasting months with higher and lower correlations.

Supplement Chapter 7 gives the intercorrelation of the atmospheric indices (potential rainfall drivers) in the form of 12 monthly correlation matrices.

Supplement Chapter 8 shows exemplary results of best subset multiple regression modelling and validation for three European areas and selected months.

2. Data Sources and Statistical Methods

2.1 Data Sources

Data for European rainfall and the examined five atmospheric indices originate from the following sources (accessed Dec. 2021 / March 2022 for gridded precipitation):

Precipitation (complete data available for period 1901-2019: CRU CY v4.04 Country Averages, variable pre),
https://crudata.uea.ac.uk/cru/data/hrg/cru_ts_4.04/crucy.2004161557.v4.04/countries/pre/

> **Gridded precipitation for supplementary analysis in ch. 9 of this supplement.**
> We exemplary analysed grid data that are provided by the CRU and used by NOAA (available only up to 2018):
>
> https://psl.noaa.gov/data/atmoswrit/timeseries/index.html
>
> The mask on this webpage was filled in as follows.
> Dataset 1: CRU_TS; Dataset 2: none
> Which variable for D1: Precipitation Rate
> Start Year: 1950; , End Year: 2018
> Time Averaging: no
> First Month Season: Jan, Last Month: Dec
> Grid point Region: see the seven grids listed in ch. 9 of this Supplement.
> Land/Ocean Mask options: Land only
> Output type: Time series
> Plot Type: Line
> Running mean Smoothing: No
> Override default Y axis Bounds Low to High: 0 to 0
> Override default Year Range/X-Axis Low to High: 0 to 0
> Markers on line plots: No
> Legend location: Top right
> Then press "create plot"
> Then download Output Products: Time-series 1 values, Data table.

NAO (complete data available for period 1825-2020),
https://crudata.uea.ac.uk/cru/data/nao/nao_3dp.dat

AO (complete data available for period 1950-2020: Monthly mean AO index):
https://www.cpc.ncep.noaa.gov/products/precip/CWlink/daily_ao_index/ao.shtml,
for period 1899-1950:
https://www.atmos.colostate.edu/~davet/ao/Data/ao_index.html

NCP: Since the CRU provides NCP values only up to 2005, we calculated the values for the period 1950-2019 ourselves, using the formulae of Kutiel and Benaroch (2002: p. 18-19). A validation of our values for 1959-1998 with those published by Kutiel & Benaroch (Table 1) proved successful. In detail, the calculation consists of four steps:

(1) Download gridded monthly geopotential heights from 1949 till today and pressures from 10-1000 mb from the source (data format netCDF):

https://iridl.ldeo.columbia.edu/SOURCES/.NOAA/.NCEP-NCAR/.CDAS-1/.MONTHLY/.Intrinsic/.PressureLevel/.phi/datafiles.html

(2) From the four-dimensional data array, extract the monthly geopotential heights for the analysis period, here 1950-2019, for pressure 500 mb ("gph500") at four grid points: A1: 55°N, 0°E; A2: 55°N, 10°E; B1: 45°N, 50°E; B2: 45°N, 60°E. For each month, average the gph500 across points A1 and A2 ("gph500.A") and across points B1 and B2 ("gph500.B").

(3) For each month, compute the difference of the resulting two geopotential heights, i.e. gph500.A – gph500.B. This is the unstandardised NCP ("NCP intensity", as termed by Kutiel & Benaroch).

(4) Standardise every monthly NCP intensity value by subtracting the mean and then dividing by the standard deviation, where mean and standard deviation were computed across all NCP intensities of the respective month in the years 1958-1998. The resulting values are the NCP values. (Remark: The years 1958-1998 were used for standardisation by Kutiel & Benaroch and should be kept because otherwise, NCP values would change retroactively as time goes by. Note that for other atmospheric indices, e.g. the NAO, also a fixed sample of years is used for standardisation.)

MOI2 (complete data available for period 1948-2020: File moi2.output.dat), https://crudata.uea.ac.uk/cru/data/moi/

Since the data are provided as daily values, we calculated monthly values as arithmetic means across all days of a month).

WeMOI (complete data available for period 1821-2019: File Web_WeMOi-2020.txt), https://crudata.uea.ac.uk/cru/data/moi/

2.2 Statistical Methods

Pearson Correlation, Simple Linear Regression, and Significance Tests. The study consists of two parts. In the first, pairwise linear correlation coefficients *r* (Pearson's product-moment correlation) between the monthly precipitation series in 39 European countries and the monthly series of the five atmospheric indices NAO, AO, NCP, MOI2, WeMOI without time lag were computed across the years 1950-2019. In order to test whether the *r* values were significantly different from zero, we relied on the theory of linear models (e.g. Fox, 2016) and applied the *t*-test for the slope of the regression line in linear least-squares regression which is zero if and only if *r* = 0, as justified in the following:

When fitting the model equation of simple linear regression

$$Y_i = \alpha + \beta\, X_i + \varepsilon_i \text{ (} i \text{ =index of year)}$$

with regression coefficients α and β and regression error ε_i to our data (a precipitation series Y_{1950}, … Y_{2019}, in the linear model conceptualised as stochastic, and an atmospheric index series x_{1950}, …, x_{2019}, conceptualised as deterministic), the least-squares fit yields estimated precipitation values

$$\hat{Y}_i = \hat{\alpha} + \hat{\beta}\, X_i$$

with estimated regression coefficients

$$\hat{\beta} = \frac{Cov(X,\ Y)}{Var(X)},\quad \hat{\alpha} = \bar{Y} - \hat{\beta}\, \bar{X}$$

where the bar indicates the mean of a data series, *Var* its variance, and *Cov* the covariance between two series. Since the Pearson correlation is

$$r = \frac{Cov(X, Y)}{\sqrt{Var(X)}\sqrt{Var(Y)}},$$

it is clear that

$$r = \hat{\beta}\ \frac{\sqrt{Var(X)}}{\sqrt{Var(Y)}}$$

so that zero correlation is equivalent to zero slope in linear regression. This holds likewise for the theoretical parameters β and ρ. This null hypothesis (H_0: β = 0 ⇔ ρ = 0) is tested with a *t* test with *n*-2 degrees of freedom (Fox, 2016, p. 111). The assumption of stochastic independence of the Y_i and thereby the ε_i values the *t*-test relies on is justified by the long-known observation that rainfall series show almost no autocorrelation (Matalas 1963; Kantelhardt et al., 2001). The normality assumption the *t*-test relies on could be dispensed with because of the central limit theorem. With *n* = 70 years and significance level $p < 0.05$, the *t*-test (df = 68) yields a critical *r* of ± 0.235, i.e. with a probability of less than 5 %, an empirical *r* will exceed this threshold by chance if the null hypothesis holds. With Bonferroni correction for multiple testing (12 x 39 = 468 *t*-tests were performed for every potential driver), the critical *r* is ±0.447, i.e. with a probability of less than 5 %, even a single one of the 468 *r* values will exceed this threshold by chance if the null hypothesis holds in every case.

Intercorrelation and Multiple Regression. In the second part of the study, the precipitation series for every month and country were modelled with multiple regressions in order to control for intercorrelations of the five atmospheric indices (shown in ch. 7, Table S6 of this Supplement). The multiple regression model with five predictors reads

$$Y_i = \alpha + \beta_1\, X_{1i} + \beta_2\, X_{2i} + \beta_3\, X_{3i} + \beta_4\, X_{4i} + \beta_5\, X_{5i} + \varepsilon_i \quad (i = \text{index of year})$$

where for the given month and country, Y_i denotes the rainfall in year *i and* X_{ki} the value of the *k*-th atmospheric index (*k* = 1, .., 5), β_k the *k*-th regression coefficient, and ε_i the stochastic regression error (assumed independently normally distributed with homogeneous variances).

Predictor Selection. It is clear that when the number of predictors in the regression equation is increased, the model fit cannot get worse. However, a multiple regression model with too many predictors, particular highly intercorrelated ones, usually leads to "overfitting" the data (i.e. modelling even the random error variance). Then the model does not describe structural relationships as intended, and is not suited to predict future rainfall data (Fox 2016, p. 690).

A best subset approach (Fox 2016, ch. 13.2.2 and ch. 22) was thus chosen for selecting those atmospheric indices as predictors that contributed substantially and omitting those that

were dispensable for explaining the variability of the rainfall in the context of the others. This method consists of two steps: First, least-squares fits to the data are obtained for the regression equations with all possible subsets of predictors (here, the set of five predictors has $2^5 - 1 = 31$ non-empty subsets, i.e. 31 regression equations were fitted to each rainfall series). Second, the outcomes are evaluated against each other. Since the goodness of fit, conceptualised as the proportion of explained variance, and its (positive) square root, which is termed multiple correlation and equals the Pearson correlation between predicted and observed rainfall,

$$R^2 = \frac{Var(\hat{Y})}{Var(Y)} \ , \quad r(\hat{Y}, Y) = \sqrt{R^2} \, ,$$

always increase with the number of predictors, several coefficients have been suggested in the literature that "penalise" the goodness of fit for the number of parameters in the model and can thus prefer a smaller model to a larger one (Fox 2016, ch. 22.1.1). We chose Mallow's Cp (Fox 2016, p. 672) to identify the best subset of predictors because it is theoretically founded but does not rely on other model assumptions than the regression model itself. With computing and comparing Mallow's Cp for all 31 possible subsets of predictors, we identified the best subset of predictors for every rainfall series and fitted the respective regression model to the data. (By the way, the purely descriptive leave-one-out cross validation criterion LOOCV, Fox 2016, p. 673, yielded the same subsets of predictors for 465 of our 468 precipitation series.)

Validation. Since it is obviously problematic to use the same data for selecting and for validating the relevant predictors, and since it is likewise problematic to base predictor selection on sequential statistical significance tests (Fox, 2016, p. 670), we applied a simple and statistically honest procedure: We divided the data into two parts, using the years 1950-2009 ("fit sample") for selecting the best subset of drivers according to Mallow's Cp (see above), and the years 2010-2019 ("validation sample") for validating the obtained model. With the fit sample, we identified the relevant predictors and obtained numerical estimations $\hat{\beta}_k$ for the regression coefficients β_k for the years 1950-2009. In the validation sample, we predicted precipitation by inserting the values of the atmospheric indices of 2010-2019 into the best subset regression equation with coefficient estimates obtained from the years 1950-2009. For validation, the so predicted precipitation values were then compared to the observed ones.

For this comparison, i.e. for assessing the correspondence between predicted rainfall $\hat{Y}$ and observed rainfall Y in the years 2010-2019, we used three criteria: first, the multiple correlation $r(\hat{Y}, Y)$between predicted and observed data (see above; please note that the correlation is insensitive to additive and multiplicative biases which is a drawback in using it for assessing the correspondence); secondly, the root mean squared error RMSE, which can be regarded as an average deviation from predicted and observed rainfall in millimetres per year

$$RMSE = \sqrt{\frac{1}{10} \sum_{i=2010}^{2019} \left(Y_i - \hat{Y}_i\right)^2} \, .$$

Thirdly, a confidence interval of prediction (PI): The PI can be computed for every probability ("confidence") between 0 and 100% and gives the interval around a predicted rainfall $\hat{Y}_i$ in which the observed rainfall Y_i will lie with the given probability if the regression obtained from the fit sample still holds in the validation sample. There is a trade-off between level of confidence and practical usefulness: The higher a confidence one requires, the larger and thus the less useful will be the interval. In this study, we decided on computing and evaluating the 70% PI: From our 10 observed rainfall data 2010-2019, 70%, i.e. at least 7 out of 10, should lie within the 70% PI if the regression model was true. This criterion was assessed for validation.

The formula for the confidence interval of prediction of level *p*, i.e. the (1-*p*) 100 % PI, is

$$\left[\hat{Y}_i - t_{n-m;\,1-\frac{p}{2}}\,\sqrt{Var(D_i)},\qquad \hat{Y}_i + t_{n-m;\,1-\frac{p}{2}}\,\sqrt{Var(D_i)}\;\right].$$

Here, $\hat{Y}_i$ is the rainfall predicted for the year *i* in the validation sample (*i* = 2010, ..., 2019). $t_{n-m;\,1-\frac{p}{2}}$ is the $1-(p/2)$ quantile of the Student *t* distribution with *n-m* degrees of freedom, where *n* is the length of the fit sample (here: 60) and *m* the number of predictors in the best subset regression model. $Var(D_i)$ is the variance of the prediction error D_i as estimated from the regression carried out in the fit sample.

Brief justification of the PI formula: D_i is a stochastic variable which under the assumptions of the multiple regression model is normally distributed with theoretical mean 0 and theoretical variance

$$V(D_i) = \sigma^2\,(1 + \mathbf{x}_i'\,(\mathbf{X}'\mathbf{X})^{-1}\,\mathbf{x}_i)$$

(Fox 2016, p. 239), where $\mathbf{x}_i$ is the vector of the *m* predictor values in year *i* and **X** the $n \times (1+m)$ matrix of the *m* predictor values (columns) in the *n*=60 years 1950-2009 (rows), including a leading column of ones for the regression intercept (**X** is termed the "design matrix" of the linear model). Since in linear regression with *m* predictors, the theoretical variance σ^2 of the normally distributed error variable ε_i is estimated by the regression error $S_E^2 = \frac{1}{n-m}\sum_{i=1}^{n}(Y_i - \hat{Y}_i)^2$, we arrive at the estimated variance $Var(D_i) = S_E^2\,(1 + \mathbf{x}_i'\,(\mathbf{X}'\mathbf{X})^{-1}\,\mathbf{x}_i)$, consequently at a *t* distribution with *n-m* degrees of freedom for the standardised D_i, and finally at the above formula for the confidence interval of prediction.

3. Pearson correlation coefficients

This chapter contains tables that list the Pearson correlation coefficients *r* between rainfall and the potential drivers NAO, AO, NCP, MOI2 and WeMOI for 39 European countries and all months of the year for the period 1950-2019. Statistically significant ($p < 0.05$) *r* values are highlighted in bold red if positive and bold blue if negative. Background colouring indicates that the coefficients remained statistically significant after Bonferroni correction across all months and countries for a given driver.

3.1 NAO vs. Precipitation (Table S1)

	Jan	Feb	Mar	Apr	May	Jun	Jul	Aug	Sept	Oct	Nov	Dec
Albania	**-0.48**	**-0.59**	**-0.51**	0.02	-0.11	-0.01	-0.06	-0.09	**-0.26**	**-0.33**	**-0.29**	**-0.39**
Austria	0.15	0.02	0.01	0.19	0.19	-0.11	-0.11	**-0.39**	-0.23	-0.16	-0.03	0.06
Belarus	0.12	-0.03	0.01	-0.22	-0.11	**-0.27**	-0.08	**-0.34**	**-0.24**	-0.23	-0.05	-0.05
Belgium	**0.40**	**0.27**	0.05	0.09	-0.03	**-0.37**	**-0.33**	**-0.40**	**-0.25**	-0.11	0.12	**0.25**
Bosnia and Herz.	**-0.45**	**-0.53**	**-0.44**	0.12	0.01	-0.19	-0.16	-0.16	**-0.47**	**-0.44**	**-0.36**	**-0.36**
Bulgaria	**-0.38**	**-0.61**	**-0.33**	0.01	-0.06	-0.01	0.01	-0.09	**-0.29**	-0.20	-0.15	-0.18
Croatia	**-0.45**	**-0.41**	**-0.42**	0.11	0.02	-0.22	-0.15	**-0.29**	**-0.46**	**-0.39**	**-0.31**	**-0.39**
Czech Republic	0.06	-0.02	-0.08	0.15	0.00	-0.12	-0.19	**-0.42**	**-0.27**	-0.10	-0.17	0.04
Denmark	**0.55**	**0.45**	**0.37**	0.16	**0.24**	-0.12	-0.18	-0.15	0.01	0.13	**0.29**	**0.55**
Estonia	**0.43**	**0.40**	**0.44**	-0.01	-0.08	-0.18	0.00	0.00	0.13	0.04	0.11	0.18
Faroe Islands	**0.65**	**0.65**	**0.56**	**0.28**	**0.50**	**0.59**	**0.33**	0.16	**0.39**	**0.44**	**0.54**	**0.65**
Finland	**0.52**	**0.43**	**0.43**	-0.03	0.05	0.04	-0.01	0.18	**0.29**	0.14	0.13	**0.44**
France	-0.07	0.04	**-0.36**	-0.16	**-0.37**	**-0.45**	**-0.35**	**-0.45**	**-0.33**	**-0.44**	-0.22	-0.10
Germany	**0.38**	**0.24**	0.16	0.19	0.07	-0.19	**-0.27**	**-0.52**	-0.20	-0.09	0.05	**0.25**
Greece	**-0.30**	**-0.46**	**-0.29**	0.07	0.05	-0.09	0.05	0.07	-0.13	-0.03	-0.15	-0.22
Hungary	**-0.36**	**-0.38**	**-0.34**	0.07	-0.12	**-0.31**	**-0.29**	**-0.31**	**-0.40**	**-0.36**	**-0.28**	**-0.30**
Iceland	**0.62**	**0.48**	**0.56**	**0.30**	**0.37**	**0.42**	**0.29**	**0.46**	0.20	**0.38**	**0.46**	**0.40**
Ireland	**0.50**	**0.55**	0.19	**0.41**	**0.24**	**-0.24**	-0.03	0.04	**0.34**	**0.24**	**0.24**	**0.53**
Italy	**-0.48**	**-0.56**	**-0.44**	-0.10	**-0.33**	**-0.25**	-0.14	-0.20	**-0.48**	**-0.36**	**-0.36**	**-0.41**
Kosovo	**-0.51**	**-0.65**	**-0.48**	-0.03	-0.05	-0.02	-0.12	-0.04	**-0.40**	**-0.40**	**-0.33**	**-0.39**
Latvia	**0.41**	**0.30**	**0.38**	-0.06	-0.11	-0.23	-0.05	-0.14	0.04	-0.06	0.09	0.06
Liechtenstein	**0.24**	0.22	0.10	0.16	**0.30**	-0.17	-0.20	**-0.48**	-0.18	-0.22	0.09	0.13
Lithuania	**0.39**	0.23	**0.28**	0.01	-0.08	-0.17	-0.09	**-0.27**	0.00	-0.10	0.08	0.09
Luxembourg	**0.36**	**0.28**	0.03	0.04	0.04	**-0.30**	**-0.33**	**-0.47**	-0.22	-0.09	0.06	0.17
North Macedonia	**-0.47**	**-0.58**	**-0.49**	0.01	-0.04	0.03	0.01	0.00	**-0.26**	**-0.29**	-0.20	**-0.32**
Moldova	**-0.37**	**-0.47**	**-0.26**	0.00	**-0.25**	-0.15	0.06	**-0.25**	**-0.33**	**-0.34**	-0.12	**-0.25**
Montenegro	**-0.52**	**-0.64**	**-0.47**	-0.03	-0.07	-0.07	-0.19	-0.12	**-0.47**	**-0.46**	**-0.38**	**-0.40**
Netherlands	**0.39**	**0.32**	0.14	0.18	-0.01	**-0.26**	**-0.32**	**-0.33**	-0.21	-0.12	0.17	**0.28**
Norway	**0.60**	**0.61**	**0.57**	-0.09	**0.43**	**0.33**	0.13	**0.25**	**0.31**	**0.46**	**0.31**	**0.46**
Poland	**0.27**	0.11	0.10	0.18	-0.02	-0.07	-0.15	**-0.42**	**-0.34**	-0.16	-0.03	0.18
Portugal	**-0.61**	**-0.53**	**-0.65**	**-0.42**	**-0.45**	**-0.28**	-0.02	**-0.32**	**-0.46**	**-0.59**	**-0.44**	**-0.67**
Romania	**-0.42**	**-0.56**	**-0.33**	0.04	-0.16	-0.22	0.02	-0.20	**-0.38**	**-0.34**	-0.19	**-0.25**
Serbia	**-0.47**	**-0.60**	**-0.46**	0.05	-0.04	-0.20	-0.13	-0.06	**-0.44**	**-0.42**	**-0.28**	**-0.33**
Slovakia	-0.14	-0.20	**-0.24**	0.08	-0.11	-0.11	**-0.28**	**-0.29**	**-0.37**	**-0.24**	-0.21	-0.14
Slovenia	**-0.33**	**-0.32**	**-0.30**	0.16	0.16	-0.06	-0.17	**-0.38**	**-0.32**	**-0.27**	-0.20	**-0.24**
Spain	**-0.66**	**-0.59**	**-0.64**	**-0.48**	**-0.55**	**-0.26**	-0.07	-0.05	**-0.44**	**-0.59**	**-0.55**	**-0.74**
Sweden	**0.49**	**0.47**	**0.46**	-0.02	0.08	0.00	-0.05	-0.01	0.05	0.15	**0.24**	**0.44**
Switzerland	0.10	0.17	-0.05	0.09	0.09	**-0.27**	-0.23	**-0.60**	**-0.25**	**-0.31**	0.00	0.05
United Kingdom	**0.58**	**0.56**	**0.29**	0.11	**0.27**	-0.20	-0.07	-0.01	**0.28**	0.09	**0.34**	**0.61**

3.2 AO vs. Precipitation (Table S2)

	Jan	Feb	Mar	Apr	May	Jun	Jul	Aug	Sept	Oct	Nov	Dec
**Albania**	-0.48	-0.53	-0.39	0.00	-0.26	0.16	0.14	0.06	-0.16	-0.36	-0.38	-0.44
**Austria**	-0.06	-0.17	-0.06	-0.04	0.03	-0.20	-0.10	-0.16	-0.31	-0.19	-0.15	-0.19
**Belarus**	-0.04	-0.19	-0.18	-0.08	-0.14	-0.39	-0.22	-0.38	-0.40	-0.32	-0.01	-0.26
**Belgium**	0.08	0.02	-0.12	-0.08	0.02	-0.37	-0.36	-0.43	-0.41	-0.23	-0.03	-0.06
**Bosnia and Herz.**	-0.49	-0.56	-0.41	0.02	-0.10	0.01	-0.02	-0.03	-0.49	-0.43	-0.35	-0.51
**Bulgaria**	-0.52	-0.55	-0.34	-0.05	-0.07	0.15	0.13	0.04	-0.25	-0.21	-0.39	-0.37
**Croatia**	-0.46	-0.47	-0.40	0.01	-0.10	-0.09	-0.03	-0.11	-0.51	-0.37	-0.30	-0.49
**Czech Republic**	-0.08	-0.21	-0.22	-0.10	-0.04	-0.24	-0.07	-0.30	-0.38	-0.29	-0.18	-0.23
**Denmark**	0.28	0.22	0.22	-0.06	0.01	-0.10	-0.26	-0.25	-0.12	-0.14	0.10	0.20
**Estonia**	0.31	0.27	0.35	0.02	-0.11	-0.27	-0.21	0.01	0.01	0.04	0.17	0.10
**Faroe Islands**	0.58	0.55	0.43	0.16	0.25	0.31	0.23	-0.02	0.40	0.26	0.41	0.55
**Finland**	0.37	0.30	0.41	0.07	-0.02	-0.11	0.01	0.00	0.24	0.21	0.12	0.38
**France**	-0.30	-0.13	-0.41	-0.24	-0.15	-0.45	-0.26	-0.42	-0.37	-0.43	-0.28	-0.29
**Germany**	0.07	-0.02	-0.05	-0.09	0.02	-0.26	-0.19	-0.44	-0.39	-0.25	-0.04	-0.05
**Greece**	-0.38	-0.35	-0.27	0.18	-0.05	0.13	0.16	0.22	-0.04	-0.11	-0.32	-0.27
**Hungary**	-0.42	-0.48	-0.42	-0.18	-0.15	-0.16	-0.13	-0.09	-0.51	-0.40	-0.33	-0.49
**Iceland**	0.53	0.60	0.57	0.24	0.34	0.44	0.19	0.35	0.28	0.28	0.34	0.52
**Ireland**	0.19	0.35	0.05	-0.11	-0.03	-0.40	-0.26	-0.24	0.03	-0.02	-0.06	0.20
**Italy**	-0.43	-0.43	-0.34	-0.10	-0.27	0.01	0.09	-0.05	-0.39	-0.32	-0.26	-0.31
**Kosovo**	-0.55	-0.64	-0.43	-0.07	-0.18	0.14	0.05	0.03	-0.36	-0.42	-0.46	-0.53
**Latvia**	0.29	0.11	0.25	-0.06	-0.07	-0.28	-0.22	-0.12	-0.17	-0.11	0.16	-0.03
**Liechtenstein**	-0.05	-0.02	-0.08	-0.01	0.09	-0.27	-0.11	-0.18	-0.37	-0.21	-0.12	-0.07
**Lithuania**	0.24	0.01	0.10	-0.06	-0.03	-0.30	-0.20	-0.29	-0.28	-0.20	0.09	-0.04
**Luxembourg**	0.04	0.00	-0.15	-0.13	-0.02	-0.30	-0.32	-0.52	-0.36	-0.22	-0.09	-0.10
**North Macedonia**	-0.49	-0.53	-0.39	-0.03	-0.10	0.16	0.21	0.18	-0.16	-0.30	-0.41	-0.45
**Moldova**	-0.54	-0.46	-0.35	-0.02	-0.20	-0.05	-0.03	-0.09	-0.40	-0.43	-0.40	-0.38
**Montenegro**	-0.54	-0.61	-0.40	-0.02	-0.18	0.09	-0.03	-0.04	-0.44	-0.45	-0.39	-0.51
**Netherlands**	0.09	0.11	-0.07	-0.07	0.07	-0.27	-0.35	-0.41	-0.37	-0.17	0.05	-0.04
**Norway**	0.69	0.64	0.64	0.37	0.30	0.31	0.18	0.17	0.55	0.56	0.50	0.68
**Poland**	0.06	-0.10	-0.06	0.04	-0.06	-0.17	-0.15	-0.39	-0.53	-0.34	-0.07	-0.08
**Portugal**	-0.55	-0.52	-0.49	-0.33	-0.24	-0.13	-0.13	-0.11	-0.31	-0.36	-0.32	-0.56
**Romania**	-0.55	-0.58	-0.41	-0.01	-0.17	-0.03	0.07	-0.08	-0.48	-0.38	-0.38	-0.46
**Serbia**	-0.58	-0.63	-0.44	-0.08	-0.17	0.03	-0.01	0.03	-0.50	-0.43	-0.40	-0.52
**Slovakia**	-0.22	-0.36	-0.36	-0.06	-0.11	-0.08	-0.16	-0.20	-0.49	-0.37	-0.24	-0.35
**Slovenia**	-0.35	-0.39	-0.27	0.02	0.01	-0.06	-0.17	-0.14	-0.33	-0.24	-0.22	-0.36
**Spain**	-0.56	-0.49	-0.43	-0.15	-0.18	-0.09	-0.01	0.07	-0.19	-0.30	-0.30	-0.56
**Sweden**	0.33	0.38	0.36	-0.01	-0.05	-0.07	-0.17	-0.10	0.13	-0.03	0.06	0.25
**Switzerland**	-0.21	-0.09	-0.20	-0.08	0.00	-0.35	-0.18	-0.31	-0.39	-0.31	-0.20	-0.12
**United Kingdom**	0.28	0.35	0.10	-0.17	0.00	-0.32	-0.27	-0.29	-0.04	-0.11	0.01	0.20

3.3 NCP vs. Precipitation (Table S3)

	Jan	Feb	Mar	Apr	May	Jun	Jul	Aug	Sept	Oct	Nov	Dec
**Albania**	-0.49	-0.38	-0.38	0.03	0.02	0.27	0.40	0.41	0.09	-0.28	-0.32	-0.48
**Austria**	-0.31	-0.63	-0.40	-0.39	-0.45	-0.24	-0.35	-0.18	-0.43	-0.49	-0.64	-0.45
**Belarus**	-0.54	-0.47	-0.44	-0.72	-0.47	-0.49	-0.44	-0.58	-0.53	-0.45	-0.59	-0.57
**Belgium**	-0.64	-0.60	-0.66	-0.65	-0.42	-0.44	-0.54	-0.57	-0.67	-0.75	-0.72	-0.62
**Bosnia and Herz.**	-0.62	-0.71	-0.69	-0.27	-0.12	0.11	0.13	0.20	-0.28	-0.56	-0.54	-0.68
**Bulgaria**	-0.50	-0.30	-0.35	0.07	0.07	0.27	0.41	0.28	0.08	-0.15	-0.19	-0.43
**Croatia**	-0.57	-0.74	-0.70	-0.31	-0.25	0.02	-0.10	0.12	-0.37	-0.54	-0.58	-0.68
**Czech Republic**	-0.25	-0.49	-0.42	-0.33	-0.51	-0.28	-0.34	-0.09	-0.52	-0.51	-0.50	-0.37
**Denmark**	-0.40	-0.45	-0.49	-0.69	-0.61	-0.52	-0.68	-0.68	-0.66	-0.65	-0.67	-0.44
**Estonia**	-0.31	-0.24	-0.22	-0.62	-0.66	-0.50	-0.73	-0.51	-0.58	-0.56	-0.54	-0.50
**Faroe Islands**	0.07	-0.06	0.00	-0.13	-0.15	0.08	0.07	-0.35	-0.15	0.04	0.06	0.06
**Finland**	-0.20	-0.25	-0.18	-0.65	-0.54	-0.38	-0.61	-0.31	-0.41	-0.38	-0.50	-0.35
**France**	-0.70	-0.77	-0.70	-0.51	-0.20	-0.27	-0.16	-0.27	-0.35	-0.59	-0.62	-0.74
**Germany**	-0.53	-0.57	-0.57	-0.62	-0.57	-0.53	-0.57	-0.40	-0.72	-0.74	-0.66	-0.54
**Greece**	-0.38	0.02	-0.09	0.22	0.09	0.33	0.43	0.50	0.12	0.05	-0.12	-0.29
**Hungary**	-0.48	-0.75	-0.70	-0.37	-0.30	-0.06	-0.16	-0.02	-0.45	-0.56	-0.49	-0.68
**Iceland**	0.44	0.51	0.49	0.51	0.59	0.55	0.41	0.60	0.31	0.54	0.50	0.55
**Ireland**	-0.32	-0.41	-0.45	-0.25	-0.30	-0.43	-0.41	-0.47	-0.16	-0.27	-0.33	-0.28
**Italy**	-0.30	-0.47	-0.44	-0.09	-0.09	0.12	0.09	0.30	-0.06	-0.20	-0.27	-0.33
**Kosovo**	-0.59	-0.55	-0.59	-0.16	-0.05	0.27	0.32	0.27	-0.03	-0.46	-0.38	-0.59
**Latvia**	-0.36	-0.26	-0.30	-0.68	-0.65	-0.57	-0.71	-0.53	-0.60	-0.57	-0.49	-0.56
**Liechtenstein**	-0.44	-0.51	-0.49	-0.58	-0.31	-0.47	-0.29	-0.23	-0.57	-0.64	-0.59	-0.48
**Lithuania**	-0.40	-0.32	-0.38	-0.71	-0.63	-0.58	-0.63	-0.55	-0.61	-0.50	-0.46	-0.51
**Luxembourg**	-0.62	-0.60	-0.67	-0.65	-0.42	-0.37	-0.50	-0.51	-0.70	-0.73	-0.72	-0.65
**North Macedonia**	-0.45	-0.35	-0.40	0.06	0.08	0.32	0.44	0.43	0.16	-0.19	-0.21	-0.44
**Moldova**	-0.49	-0.25	-0.43	-0.20	0.02	-0.16	0.01	0.11	-0.04	-0.43	-0.22	-0.32
**Montenegro**	-0.60	-0.64	-0.64	-0.21	-0.08	0.18	0.19	0.27	-0.19	-0.51	-0.49	-0.64
**Netherlands**	-0.64	-0.57	-0.60	-0.63	-0.51	-0.52	-0.64	-0.60	-0.69	-0.79	-0.73	-0.60
**Norway**	0.36	0.28	0.31	-0.07	-0.11	-0.06	-0.23	-0.23	0.08	0.28	0.31	0.37
**Poland**	-0.40	-0.40	-0.46	-0.56	-0.56	-0.42	-0.52	-0.50	-0.65	-0.53	-0.49	-0.37
**Portugal**	-0.31	-0.35	-0.24	0.04	0.19	0.11	0.09	0.00	0.17	-0.07	-0.02	-0.13
**Romania**	-0.60	-0.47	-0.52	-0.28	-0.09	-0.05	0.08	0.13	-0.20	-0.46	-0.28	-0.50
**Serbia**	-0.64	-0.64	-0.65	-0.28	-0.16	0.14	0.24	0.19	-0.20	-0.53	-0.40	-0.65
**Slovakia**	-0.36	-0.64	-0.55	-0.40	-0.32	-0.10	-0.24	-0.09	-0.52	-0.56	-0.52	-0.54
**Slovenia**	-0.46	-0.72	-0.59	-0.29	-0.37	-0.11	-0.27	-0.04	-0.36	-0.47	-0.60	-0.61
**Spain**	-0.32	-0.28	-0.26	0.12	0.22	0.21	0.17	0.34	0.26	-0.10	-0.06	-0.18
**Sweden**	-0.18	-0.21	-0.26	-0.66	-0.53	-0.54	-0.62	-0.60	-0.48	-0.49	-0.53	-0.41
**Switzerland**	-0.60	-0.69	-0.62	-0.71	-0.32	-0.48	-0.23	-0.37	-0.54	-0.68	-0.66	-0.59
**United Kingdom**	-0.38	-0.45	-0.54	-0.59	-0.45	-0.54	-0.49	-0.65	-0.39	-0.50	-0.49	-0.32

3.4 MOI2 vs. Precipitation (Table S4)

	Jan	Feb	Mar	Apr	May	Jun	Jul	Aug	Sept	Oct	Nov	Dec
**Albania**	-0.36	-0.43	-0.37	0.12	0.02	-0.31	-0.37	-0.48	-0.31	-0.22	-0.09	-0.32
**Austria**	0.34	0.04	0.01	0.34	0.00	-0.06	-0.11	0.04	-0.14	0.05	0.26	0.36
**Belarus**	0.17	0.03	0.12	0.07	0.03	-0.07	0.10	0.08	-0.10	0.14	0.21	0.17
**Belgium**	0.46	0.23	0.11	0.31	0.22	-0.03	-0.02	-0.10	-0.02	0.15	0.31	0.43
**Bosnia and Herz.**	-0.39	-0.49	-0.32	0.17	-0.16	-0.26	-0.44	-0.32	-0.34	-0.21	-0.13	-0.24
**Bulgaria**	-0.10	-0.37	-0.14	0.05	-0.07	-0.16	-0.30	-0.39	-0.28	-0.13	0.03	0.03
**Croatia**	-0.46	-0.49	-0.36	0.12	-0.10	-0.27	-0.29	-0.36	-0.31	-0.20	-0.15	-0.31
**Czech Republic**	0.26	0.13	-0.07	0.25	0.16	-0.05	-0.12	0.14	-0.15	0.15	0.06	0.35
**Denmark**	0.55	0.28	0.24	0.32	0.43	0.11	0.17	0.13	0.17	0.31	0.38	0.55
**Estonia**	0.40	0.31	0.28	0.30	0.20	-0.08	0.15	0.30	0.11	0.27	0.28	0.31
**Faroe Islands**	0.48	0.51	0.38	0.22	0.34	0.19	0.19	0.04	0.13	0.31	0.37	0.48
**Finland**	0.53	0.35	0.28	0.27	0.10	0.06	0.05	0.26	0.30	0.27	0.25	0.39
**France**	-0.13	-0.19	-0.23	0.06	0.04	0.06	-0.01	-0.14	0.03	-0.38	-0.17	0.00
**Germany**	0.56	0.22	0.19	0.41	0.30	-0.05	-0.10	0.12	-0.01	0.28	0.30	0.49
**Greece**	0.01	-0.08	-0.09	0.06	0.22	-0.17	-0.39	-0.36	-0.22	-0.07	0.00	-0.02
**Hungary**	-0.30	-0.33	-0.26	0.16	-0.22	-0.20	-0.20	-0.23	-0.24	-0.08	-0.05	-0.06
**Iceland**	0.46	0.35	0.27	-0.05	-0.06	-0.13	-0.13	-0.30	-0.10	0.12	0.17	0.18
**Ireland**	0.36	0.26	0.25	0.37	0.25	0.05	0.27	0.06	0.26	0.16	0.14	0.37
**Italy**	-0.47	-0.57	-0.42	0.09	-0.17	-0.20	-0.37	-0.31	-0.38	-0.37	-0.28	-0.46
**Kosovo**	-0.31	-0.48	-0.30	0.06	-0.07	-0.29	-0.37	-0.44	-0.35	-0.21	-0.07	-0.20
**Latvia**	0.37	0.23	0.26	0.26	0.16	-0.13	0.10	0.24	0.06	0.25	0.29	0.26
**Liechtenstein**	0.45	0.21	0.18	0.41	0.13	0.07	-0.13	0.07	-0.05	0.07	0.33	0.41
**Lithuania**	0.37	0.18	0.21	0.30	0.15	-0.14	0.09	0.12	0.06	0.23	0.28	0.29
**Luxembourg**	0.46	0.21	0.10	0.24	0.23	-0.06	-0.03	-0.06	0.02	0.19	0.27	0.38
**North Macedonia**	-0.26	-0.40	-0.31	0.06	0.10	-0.21	-0.31	-0.43	-0.28	-0.22	-0.05	-0.16
**Moldova**	-0.13	-0.29	-0.07	0.16	-0.35	-0.05	0.04	-0.14	-0.30	-0.14	0.05	-0.04
**Montenegro**	-0.44	-0.53	-0.35	0.07	-0.14	-0.26	-0.44	-0.41	-0.36	-0.24	-0.14	-0.31
**Netherlands**	0.48	0.25	0.20	0.34	0.34	-0.07	-0.02	-0.02	0.03	0.17	0.37	0.46
**Norway**	0.57	0.55	0.36	-0.16	0.20	0.29	0.29	0.15	0.30	0.40	0.28	0.30
**Poland**	0.39	0.17	0.06	0.39	0.15	-0.01	0.06	0.11	-0.12	0.22	0.18	0.40
**Portugal**	-0.74	-0.69	-0.54	-0.45	-0.17	0.04	0.02	-0.17	-0.32	-0.74	-0.56	-0.67
**Romania**	-0.16	-0.34	-0.12	0.14	-0.32	-0.15	-0.11	-0.34	-0.34	-0.11	0.05	0.00
**Serbia**	-0.26	-0.43	-0.31	0.13	-0.17	-0.25	-0.36	-0.31	-0.31	-0.19	-0.02	-0.13
**Slovakia**	-0.06	-0.10	-0.20	0.24	-0.14	-0.09	-0.14	-0.06	-0.26	0.06	0.01	0.15
**Slovenia**	-0.37	-0.41	-0.34	0.15	-0.06	-0.14	-0.17	-0.19	-0.15	-0.10	-0.06	-0.19
**Spain**	-0.78	-0.72	-0.64	-0.45	-0.21	-0.04	-0.18	-0.28	-0.30	-0.77	-0.68	-0.77
**Sweden**	0.51	0.36	0.32	0.18	0.15	0.18	0.14	0.35	0.16	0.33	0.33	0.41
**Switzerland**	0.23	0.06	0.02	0.32	0.15	0.06	-0.08	0.01	-0.04	-0.08	0.19	0.26
**United Kingdom**	0.43	0.27	0.27	0.25	0.36	0.06	0.15	0.16	0.27	0.13	0.23	0.51

3.5 WeMOI vs. Precipitation (Table S5)

	Jan	Feb	Mar	Apr	May	Jun	Jul	Aug	Sept	Oct	Nov	Dec
**Albania**	0.33	0.16	0.08	0.46	0.19	-0.15	0.02	0.04	0.01	0.26	0.33	0.41
**Austria**	0.64	0.67	0.49	0.57	0.29	0.39	0.42	0.21	0.34	0.58	0.69	0.64
**Belarus**	0.44	0.38	0.42	0.30	0.18	0.24	0.30	0.27	0.21	0.38	0.47	0.51
**Belgium**	0.73	0.65	0.65	0.67	0.28	0.33	0.34	0.50	0.50	0.57	0.73	0.73
**Bosnia and Herz.**	0.35	0.42	0.38	0.49	0.26	0.08	0.28	0.13	0.29	0.45	0.51	0.57
**Bulgaria**	0.39	0.06	0.17	0.18	0.04	0.00	0.00	-0.05	-0.03	-0.03	0.15	0.44
**Croatia**	0.29	0.40	0.43	0.51	0.33	0.22	0.38	0.16	0.31	0.51	0.51	0.54
**Czech Republic**	0.48	0.53	0.41	0.46	0.29	0.39	0.42	0.30	0.30	0.63	0.55	0.58
**Denmark**	0.56	0.46	0.47	0.55	0.32	0.04	0.14	0.29	0.38	0.50	0.62	0.49
**Estonia**	0.27	0.27	0.28	0.33	0.28	-0.10	0.22	-0.16	0.14	0.36	0.45	0.36
**Faroe Islands**	0.13	0.27	0.17	0.17	0.07	-0.24	-0.17	-0.03	-0.05	-0.06	0.03	0.14
**Finland**	0.27	0.29	0.22	0.27	-0.08	-0.14	0.10	-0.20	0.07	0.22	0.34	0.32
**France**	0.43	0.61	0.51	0.42	0.19	0.45	0.28	0.61	0.45	0.26	0.44	0.72
**Germany**	0.77	0.63	0.61	0.66	0.39	0.44	0.35	0.49	0.50	0.67	0.68	0.68
**Greece**	0.46	-0.03	-0.01	0.13	0.16	-0.13	-0.03	-0.13	-0.02	-0.07	0.10	0.37
**Hungary**	0.31	0.48	0.48	0.47	0.17	0.24	0.45	0.13	0.29	0.50	0.49	0.66
**Iceland**	0.01	-0.28	-0.24	-0.39	-0.24	-0.15	-0.16	-0.31	-0.41	-0.34	-0.28	-0.35
**Ireland**	0.38	0.49	0.43	0.52	0.09	0.15	0.14	0.24	0.37	0.20	0.25	0.37
**Italy**	0.17	0.18	0.26	0.48	0.21	0.19	0.14	0.15	0.10	0.28	0.24	0.24
**Kosovo**	0.36	0.27	0.28	0.46	0.20	-0.05	0.15	0.00	0.08	0.33	0.34	0.51
**Latvia**	0.34	0.26	0.34	0.34	0.32	0.01	0.15	-0.01	0.21	0.43	0.42	0.42
**Liechtenstein**	0.72	0.65	0.60	0.57	0.26	0.49	0.31	0.33	0.48	0.58	0.65	0.66
**Lithuania**	0.43	0.35	0.41	0.39	0.29	0.19	0.21	0.14	0.27	0.46	0.40	0.44
**Luxembourg**	0.72	0.66	0.62	0.60	0.34	0.32	0.35	0.51	0.52	0.54	0.72	0.74
**North Macedonia**	0.32	0.12	0.10	0.34	0.09	-0.13	0.00	-0.17	-0.06	0.11	0.19	0.44
**Moldova**	0.32	0.12	0.31	0.25	-0.02	0.18	0.14	0.27	0.12	0.22	0.21	0.33
**Montenegro**	0.31	0.33	0.32	0.48	0.25	-0.01	0.22	0.10	0.24	0.42	0.45	0.50
**Netherlands**	0.71	0.61	0.62	0.67	0.33	0.26	0.27	0.48	0.50	0.57	0.72	0.69
**Norway**	-0.03	-0.05	-0.08	-0.21	-0.14	-0.22	-0.11	-0.23	-0.19	-0.13	-0.16	-0.31
**Poland**	0.57	0.44	0.42	0.55	0.28	0.25	0.40	0.31	0.30	0.54	0.47	0.52
**Portugal**	-0.27	-0.12	-0.14	-0.30	-0.30	0.19	-0.02	0.18	-0.12	-0.45	-0.36	-0.19
**Romania**	0.37	0.28	0.36	0.29	0.06	0.23	0.23	0.11	0.20	0.26	0.28	0.51
**Serbia**	0.39	0.38	0.34	0.44	0.19	0.13	0.25	0.04	0.23	0.36	0.41	0.59
**Slovakia**	0.35	0.49	0.44	0.48	0.17	0.26	0.38	0.18	0.25	0.54	0.53	0.63
**Slovenia**	0.28	0.46	0.39	0.48	0.32	0.27	0.37	0.13	0.28	0.51	0.52	0.56
**Spain**	-0.25	-0.19	-0.20	-0.36	-0.25	0.24	-0.03	0.20	-0.29	-0.36	-0.37	-0.17
**Sweden**	0.29	0.28	0.30	0.38	-0.05	-0.02	0.12	0.13	0.10	0.41	0.46	0.36
**Switzerland**	0.69	0.71	0.62	0.58	0.31	0.53	0.32	0.51	0.49	0.54	0.64	0.70
**United Kingdom**	0.45	0.50	0.54	0.55	0.17	0.19	0.07	0.29	0.51	0.24	0.36	0.46

4. Correlation Coefficient Maps

The maps in Figures S1-S5 show the most positive (left column) and most negative (right column) correlation coefficients for monthly rainfall compared to, NAO, AO, NCP, MOI2 and WeMOI on a country-by-country basis. Pearson *r* values are from Tables S1-S5. In order not to focus on statistically significant but spurious correlations, only *r* values for *r* equal or better than ±0.40 are shown.

The geographical base map of Europe was retrieved from https://www.d-maps.com/carte.php?num_car=2232&lang=en.

4.1 NAO vs. Precipitation (Figure S1)

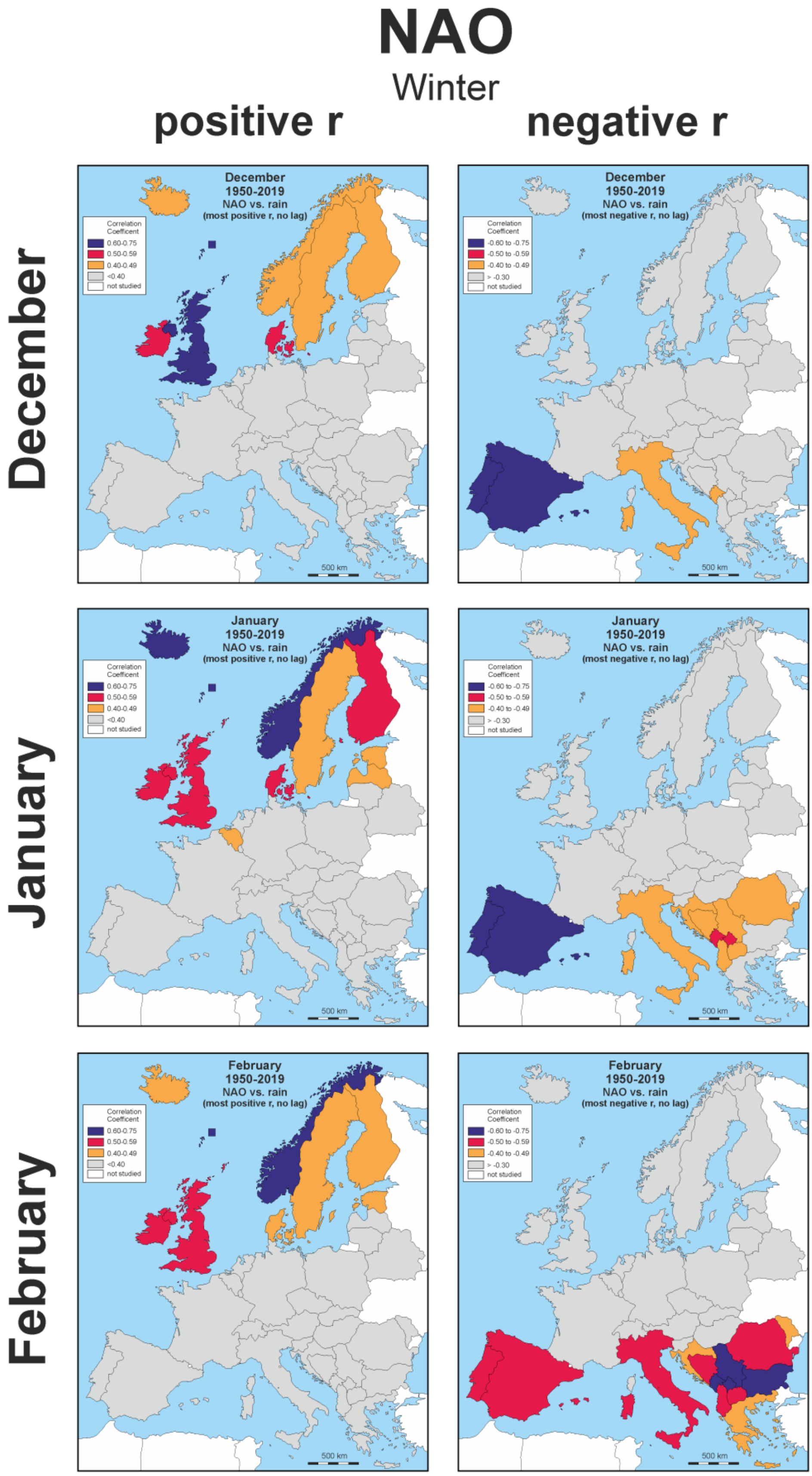

NAO

Spring

positive r **negative r**

March **April** **May**

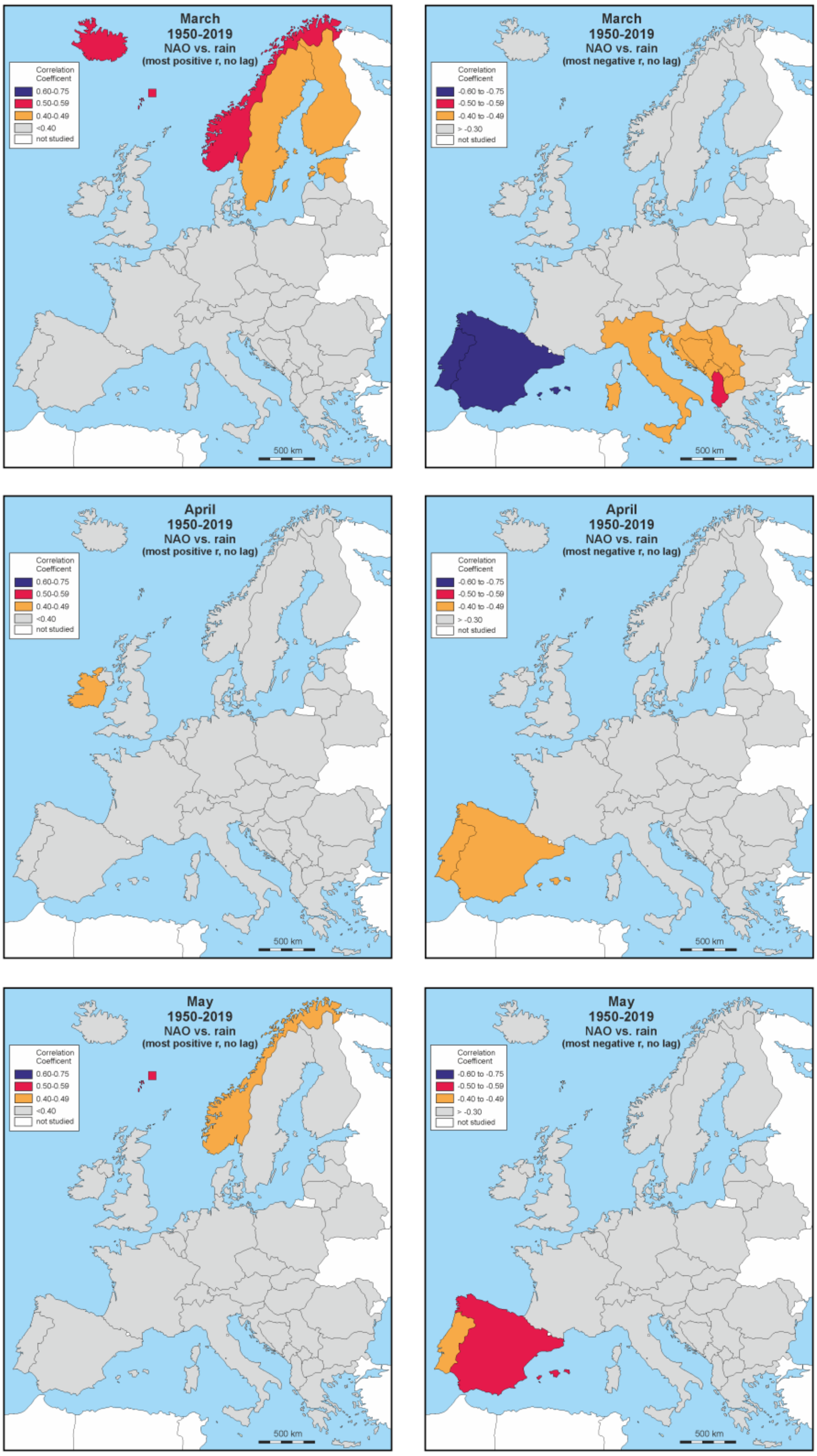

NAO

Summer

positive r **negative r**

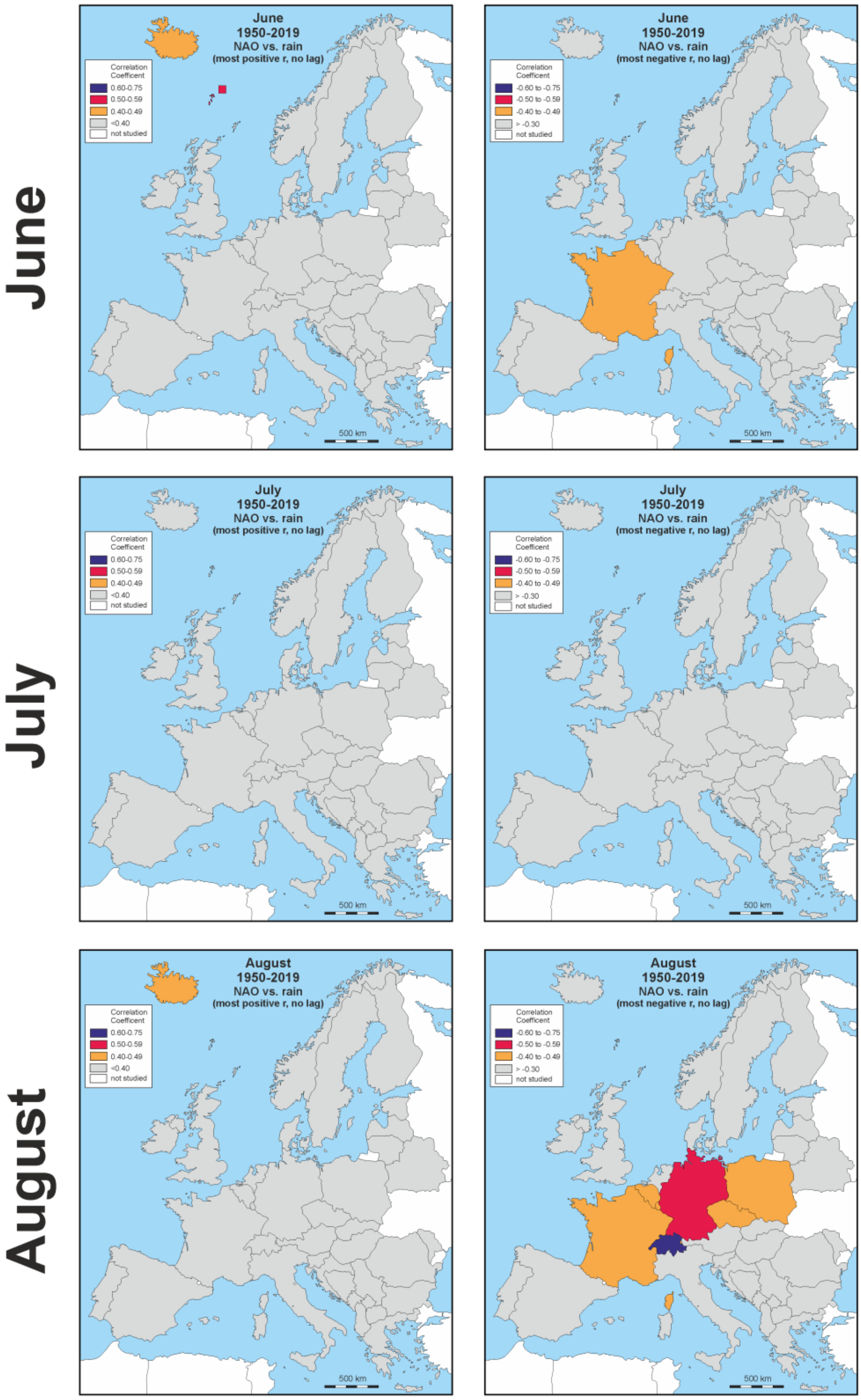

NAO

Autumn

positive r

negative r

September

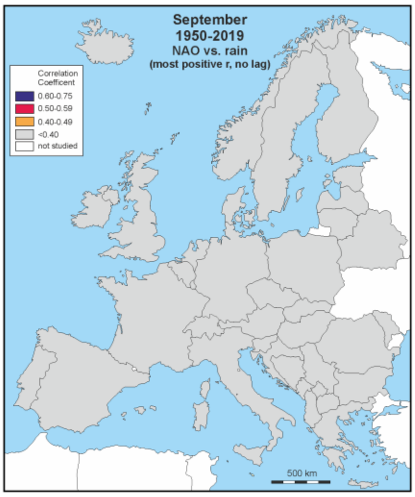

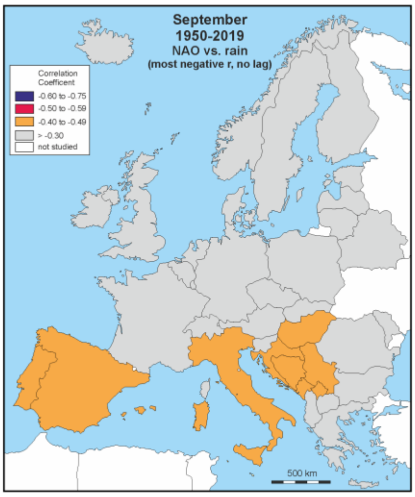

October

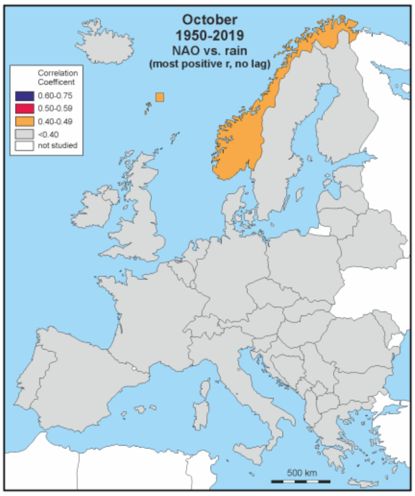

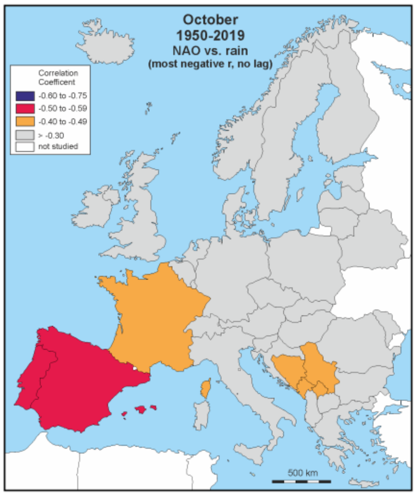

November

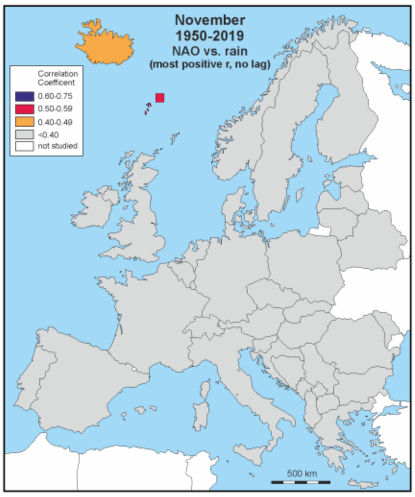

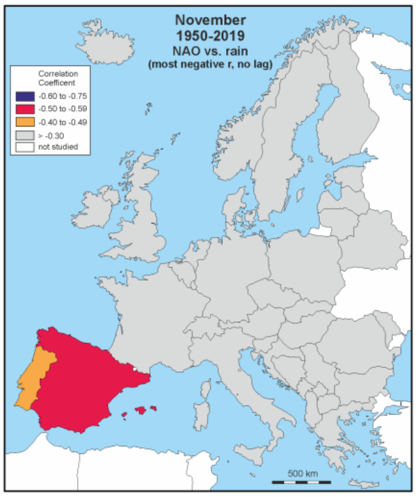

4.2 AO vs. Precipitation (Figure S2)

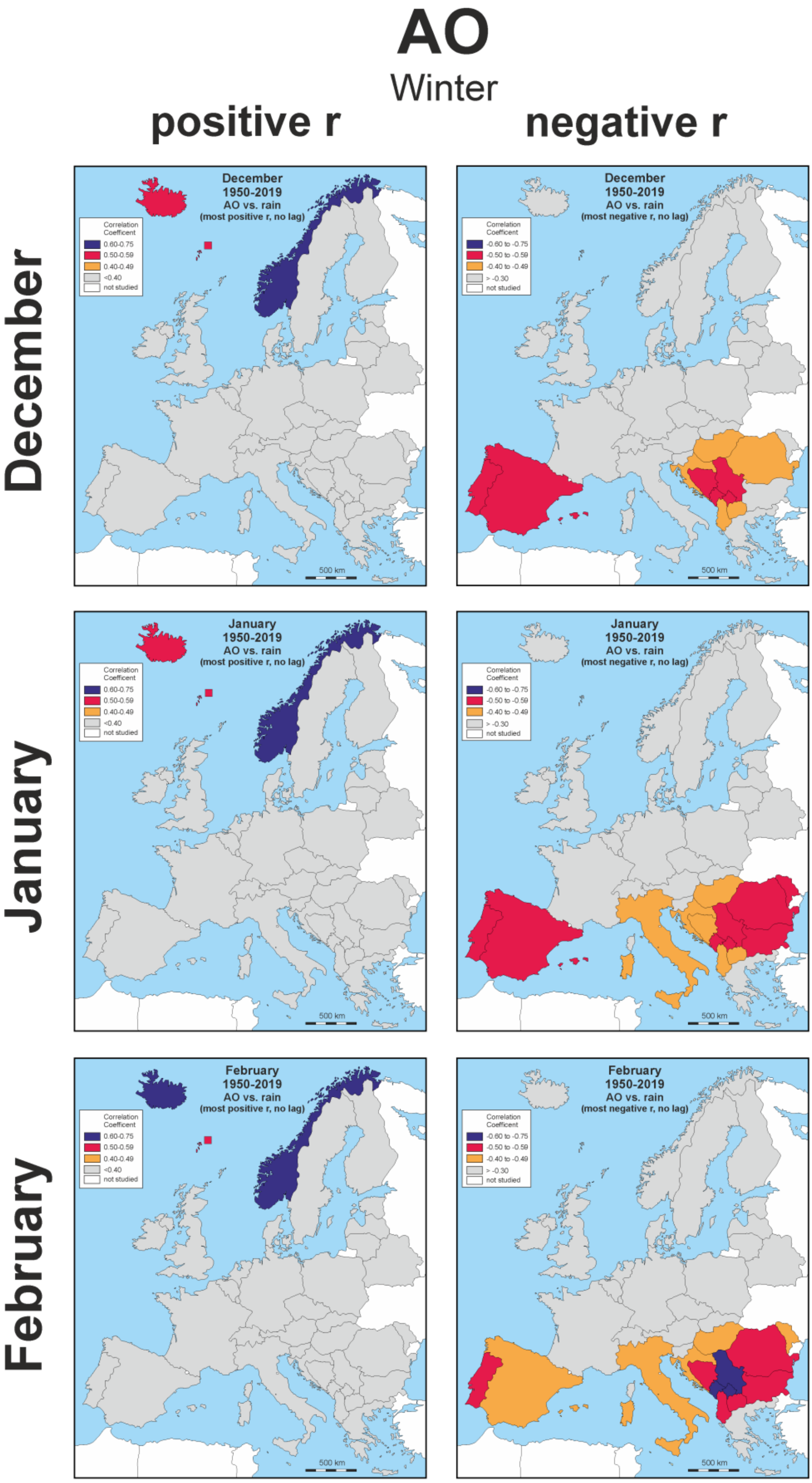

AO

Spring

positive r **negative r**

March **April** **May**

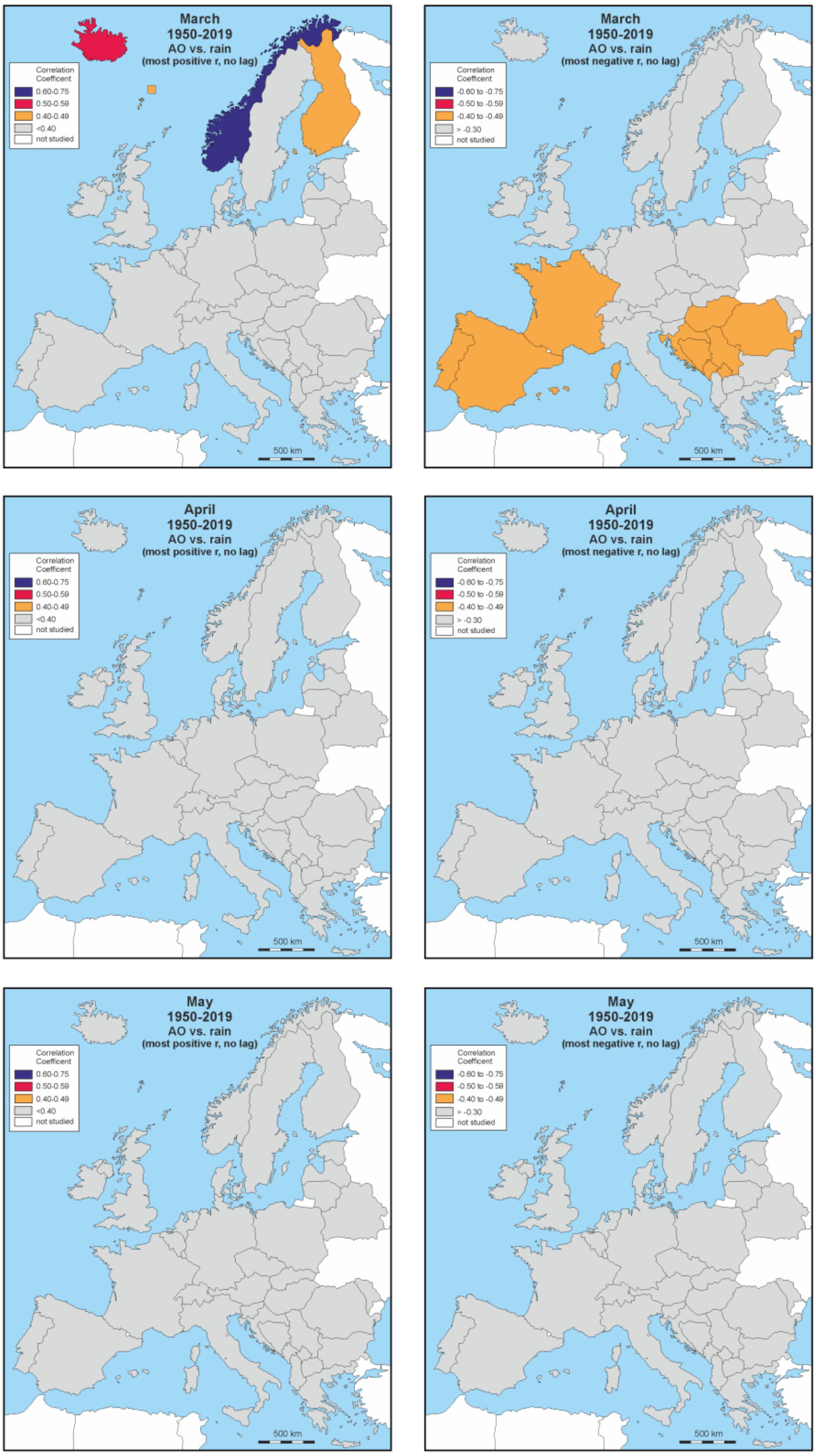

AO

Summer

positive r | negative r

June | July | August

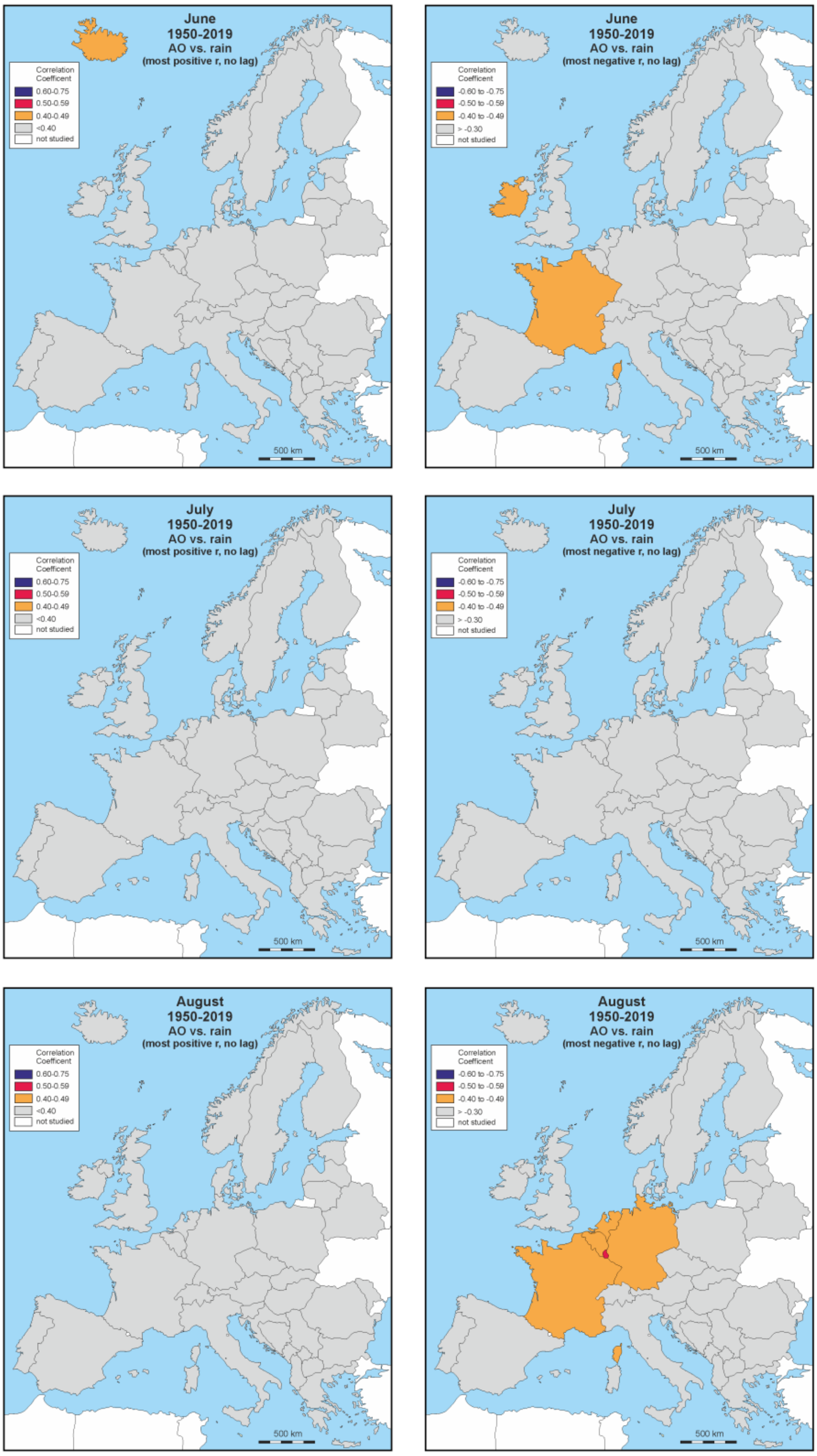

AO

Autumn

positive r **negative r**

September

October

November

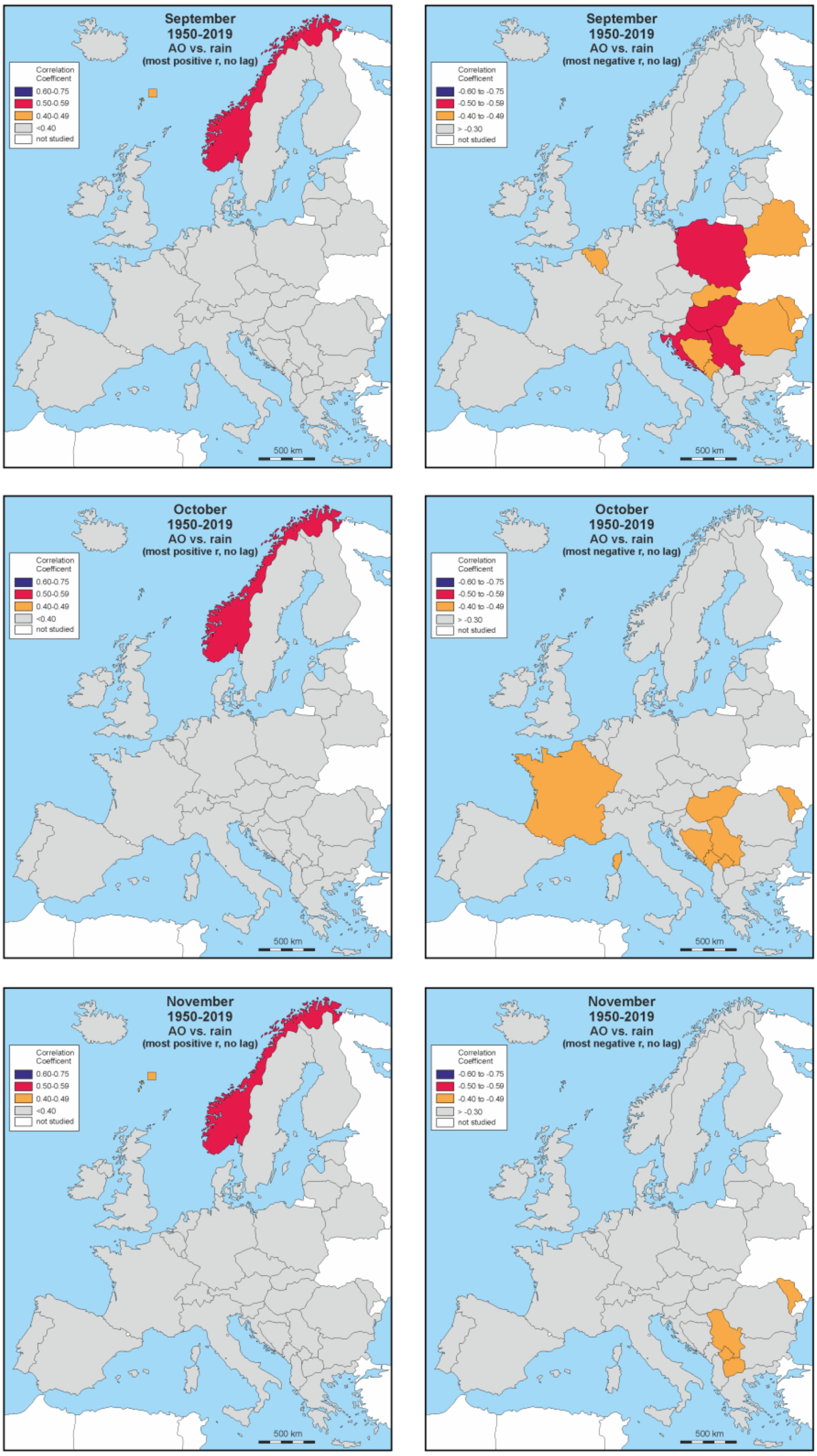

4.3 NCP vs. Precipitation (Figure S3)

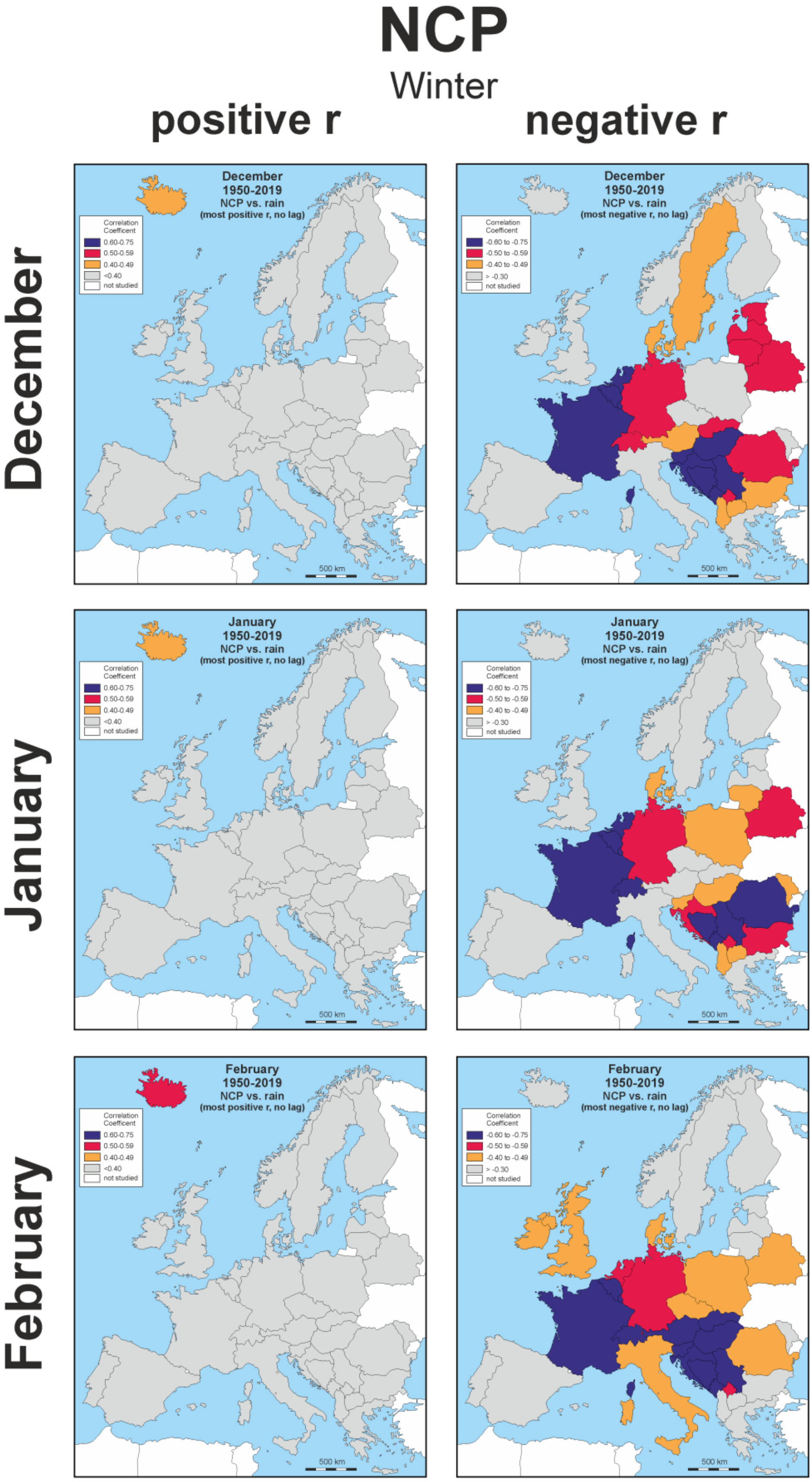

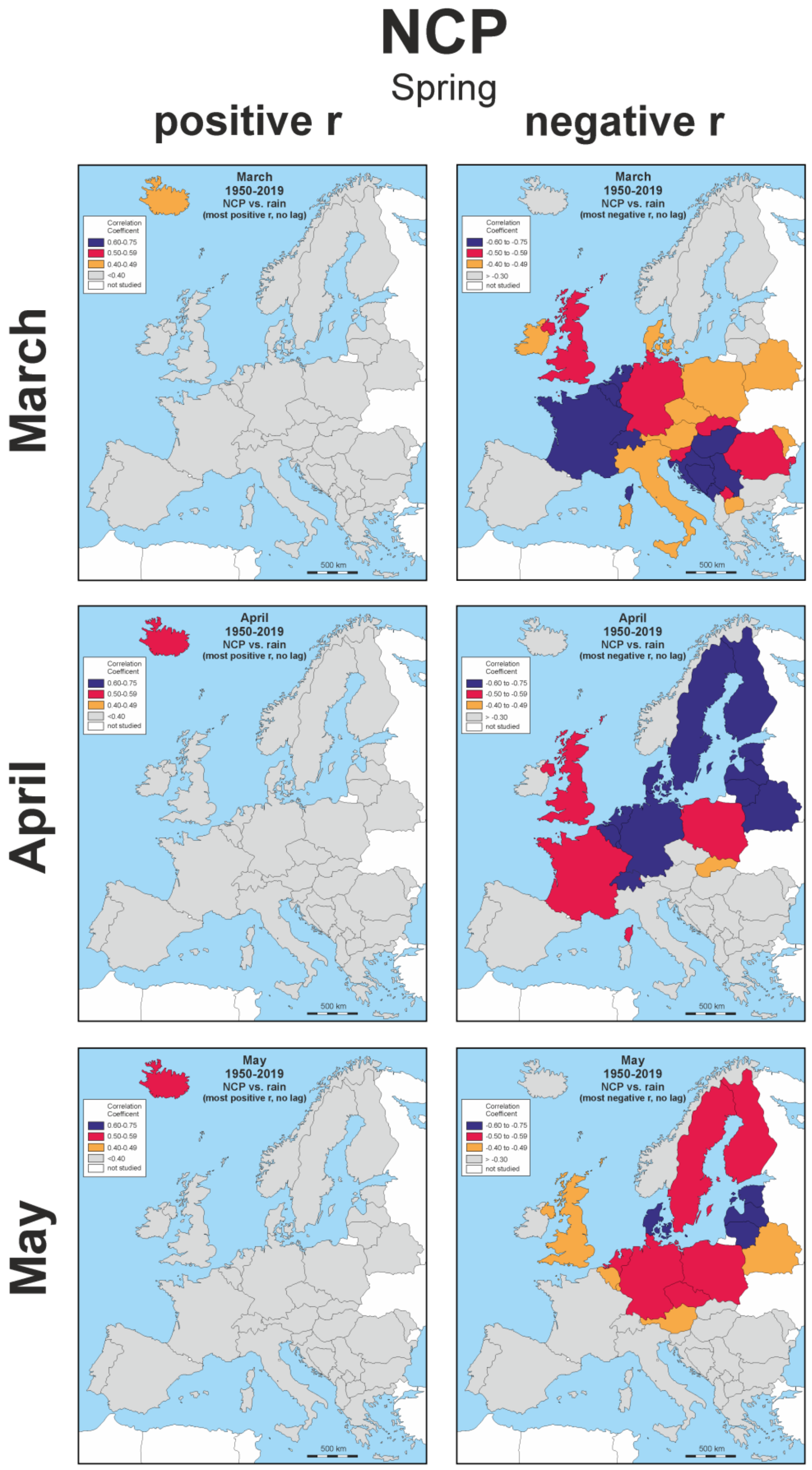
NCP
Spring
positive r
negative r
March
April
May
March
1950-2019
NCP vs. rain
(most positive r, no lag)
Correlation Coefficent
0.60-0.75
0.50-0.59
0.40-0.49
<0.40
not studied
500 km
March
1950-2019
NCP vs. rain
(most negative r, no lag)
Correlation Coefficent
-0.60 to -0.75
-0.50 to -0.59
-0.40 to -0.49
> -0.30
not studied
500 km
April
1950-2019
NCP vs. rain
(most positive r, no lag)
Correlation Coefficent
0.60-0.75
0.50-0.59
0.40-0.49
<0.40
not studied
500 km
April
1950-2019
NCP vs. rain
(most negative r, no lag)
Correlation Coefficent
-0.60 to -0.75
-0.50 to -0.59
-0.40 to -0.49
> -0.30
not studied
500 km
May
1950-2019
NCP vs. rain
(most positive r, no lag)
Correlation Coefficent
0.60-0.75
0.50-0.59
0.40-0.49
<0.40
not studied
500 km
May
1950-2019
NCP vs. rain
(most negative r, no lag)
Correlation Coefficent
-0.60 to -0.75
-0.50 to -0.59
-0.40 to -0.49
> -0.30
not studied
500 km

NCP

Summer

positive r **negative r**

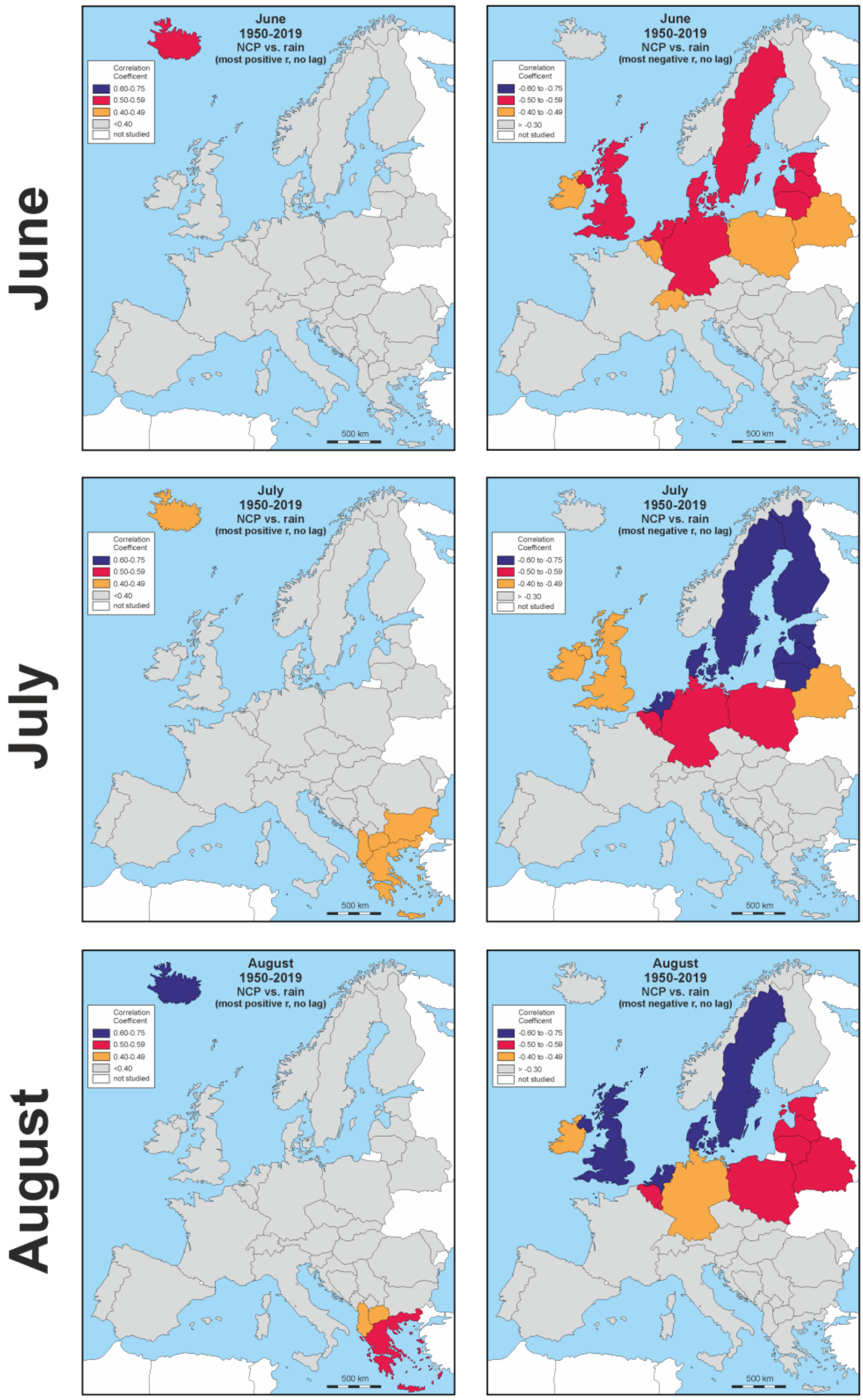

NCP

Autumn

positive r | **negative r**

September

October

November

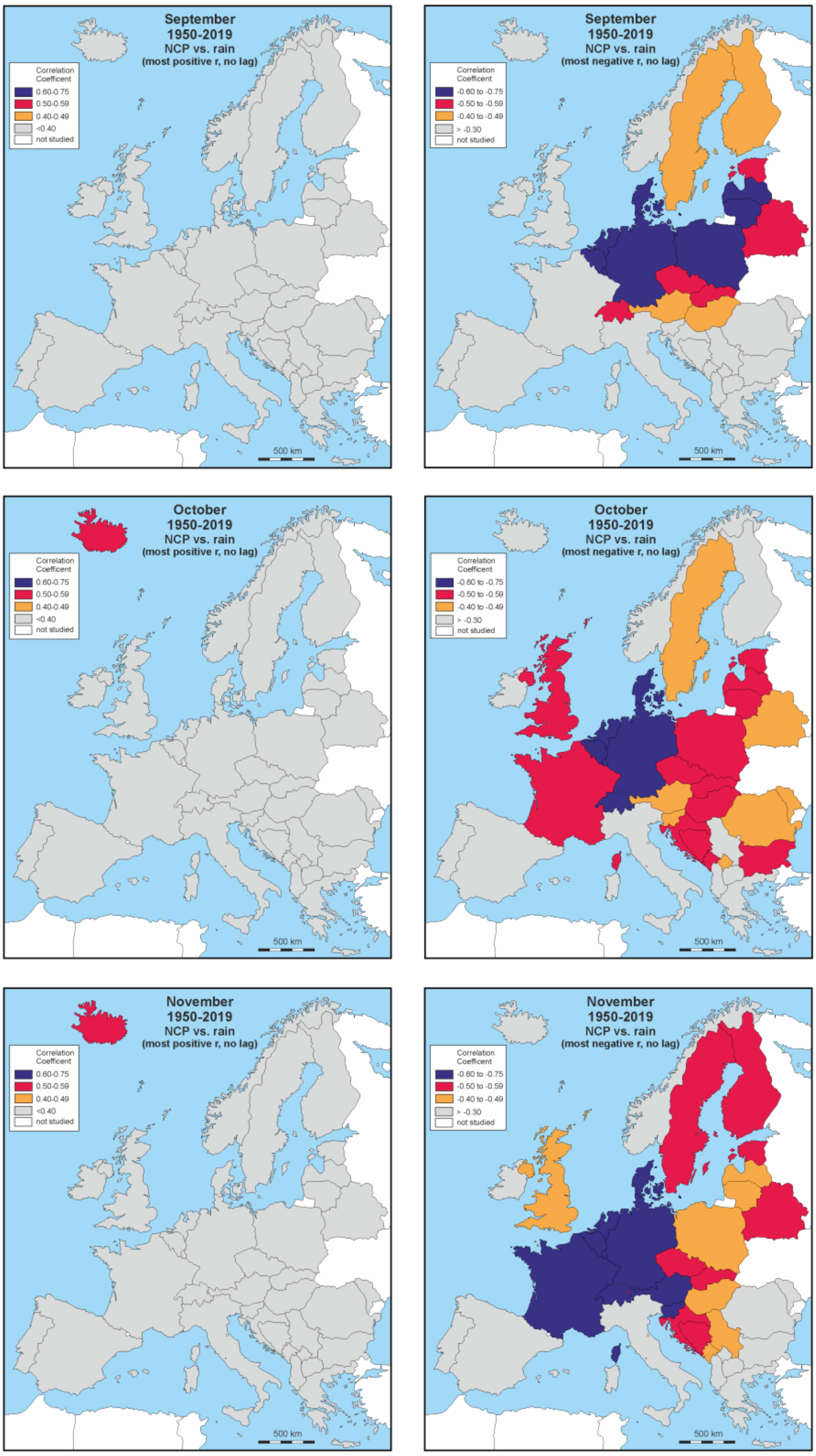

4.4 MOI2 vs. Precipitation (Figure S4)

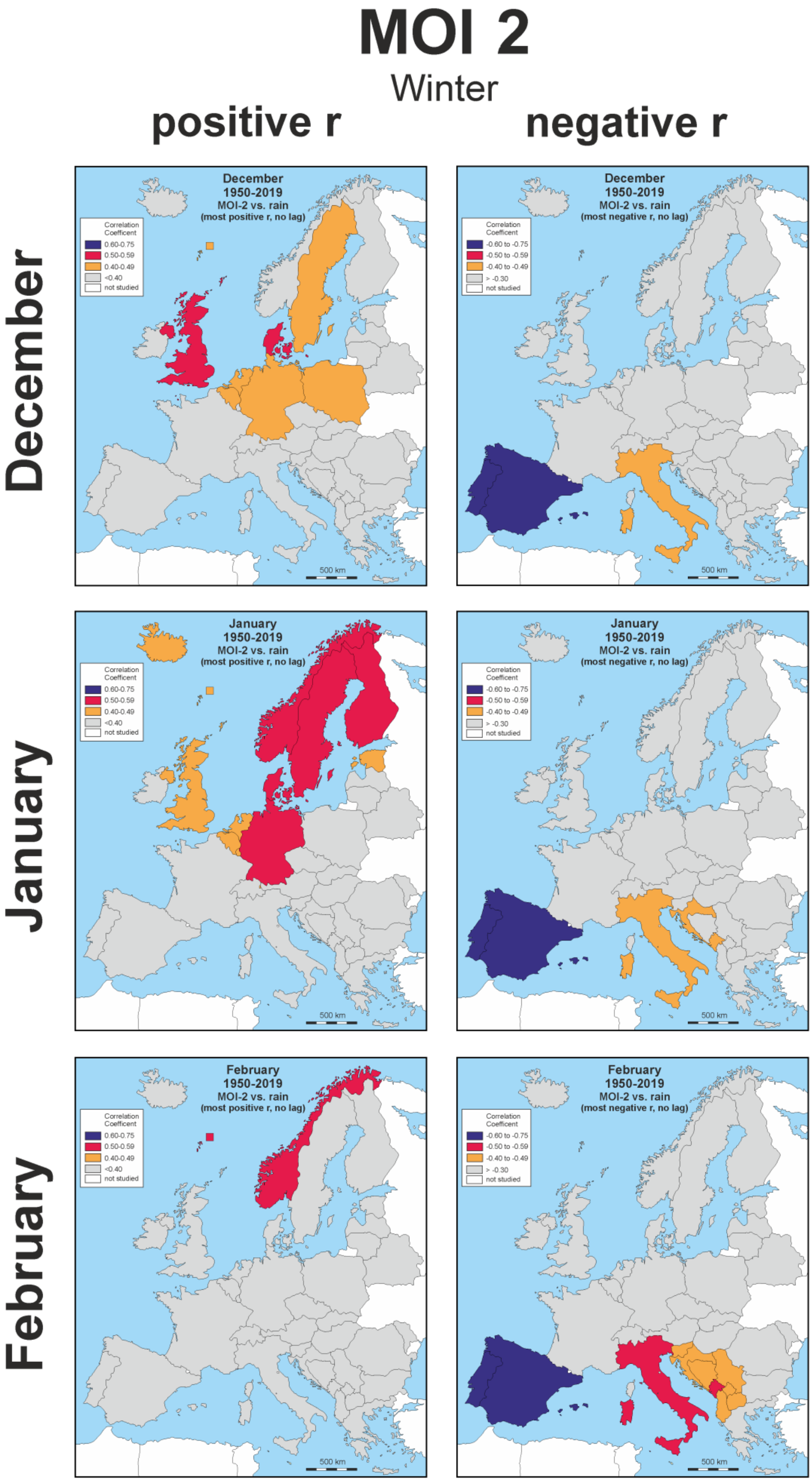

MOI 2

Spring

positive r **negative r**

March

April

May

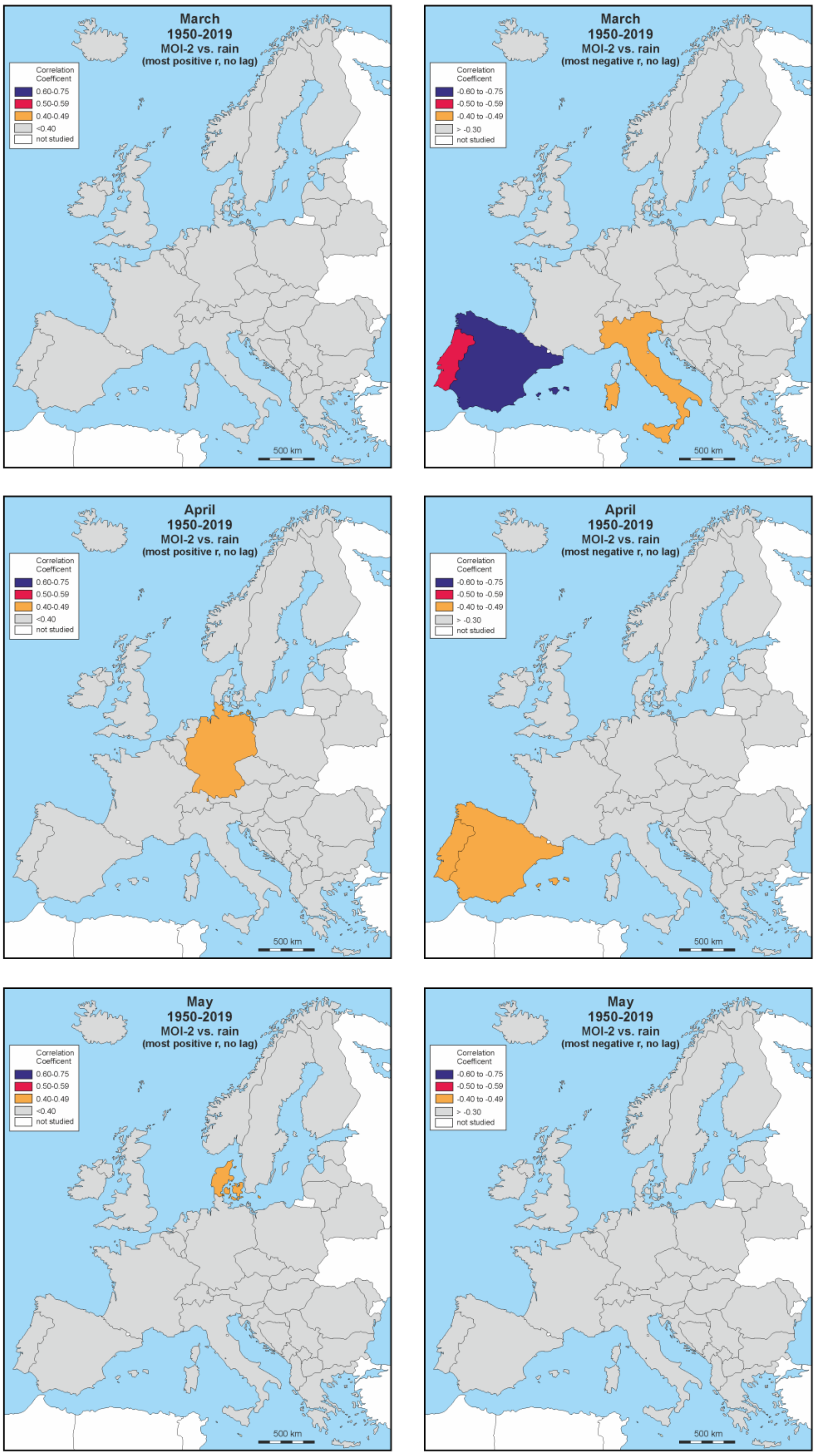

MOI 2

Summer

positive r **negative r**

June

July

August

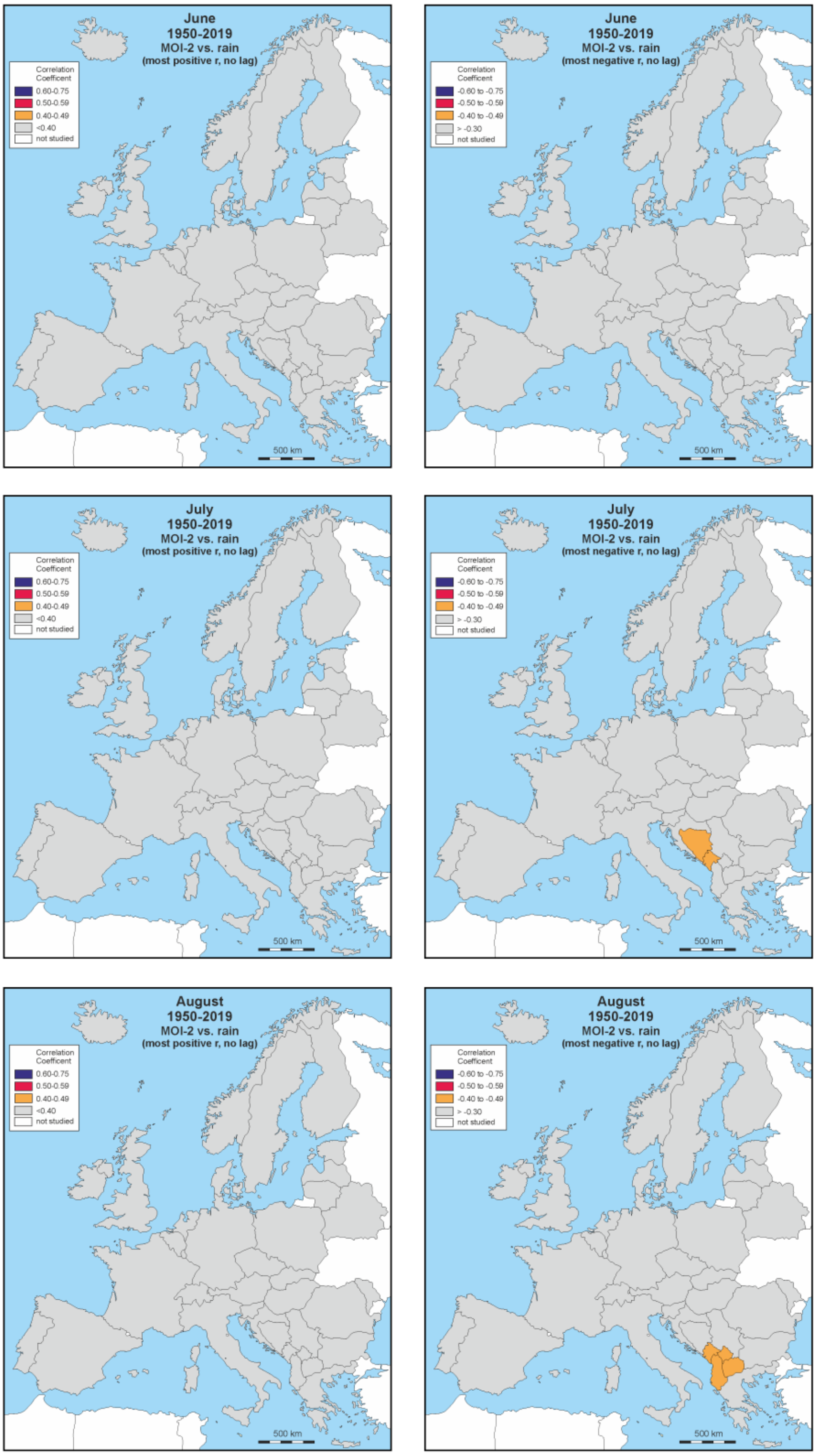

MOI 2

Autumn

positive r | **negative r**

September

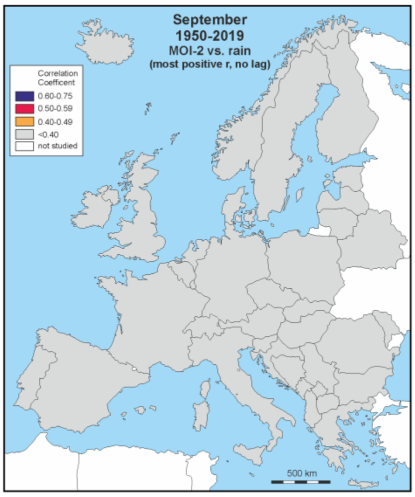

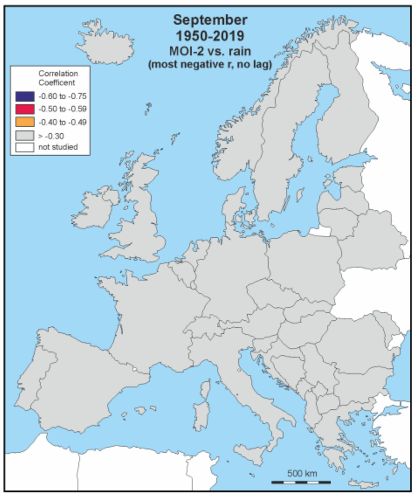

October

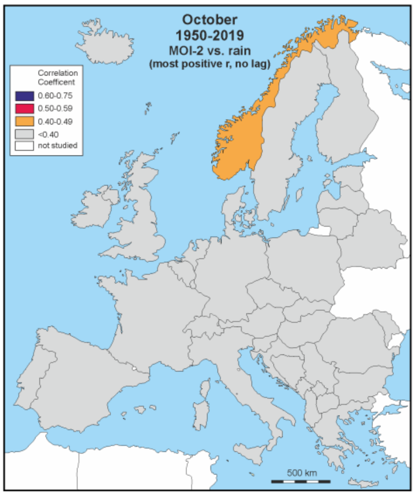

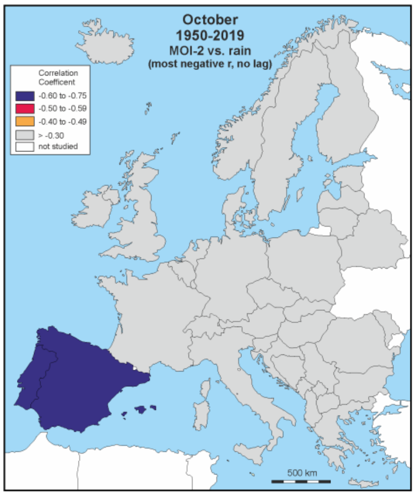

November

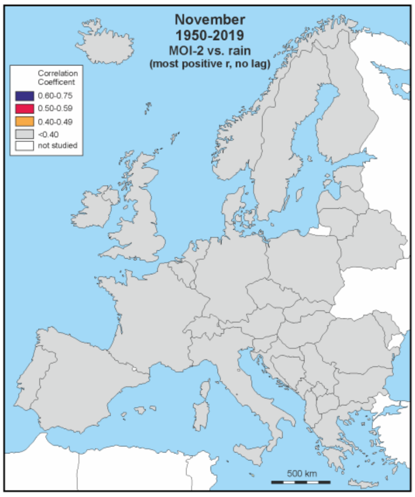

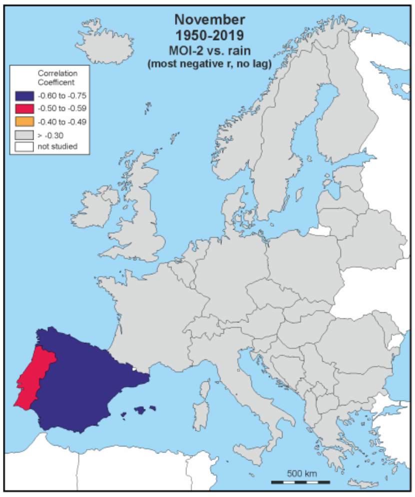

4.5 WeMOI vs. Precipitation (Figure S5)

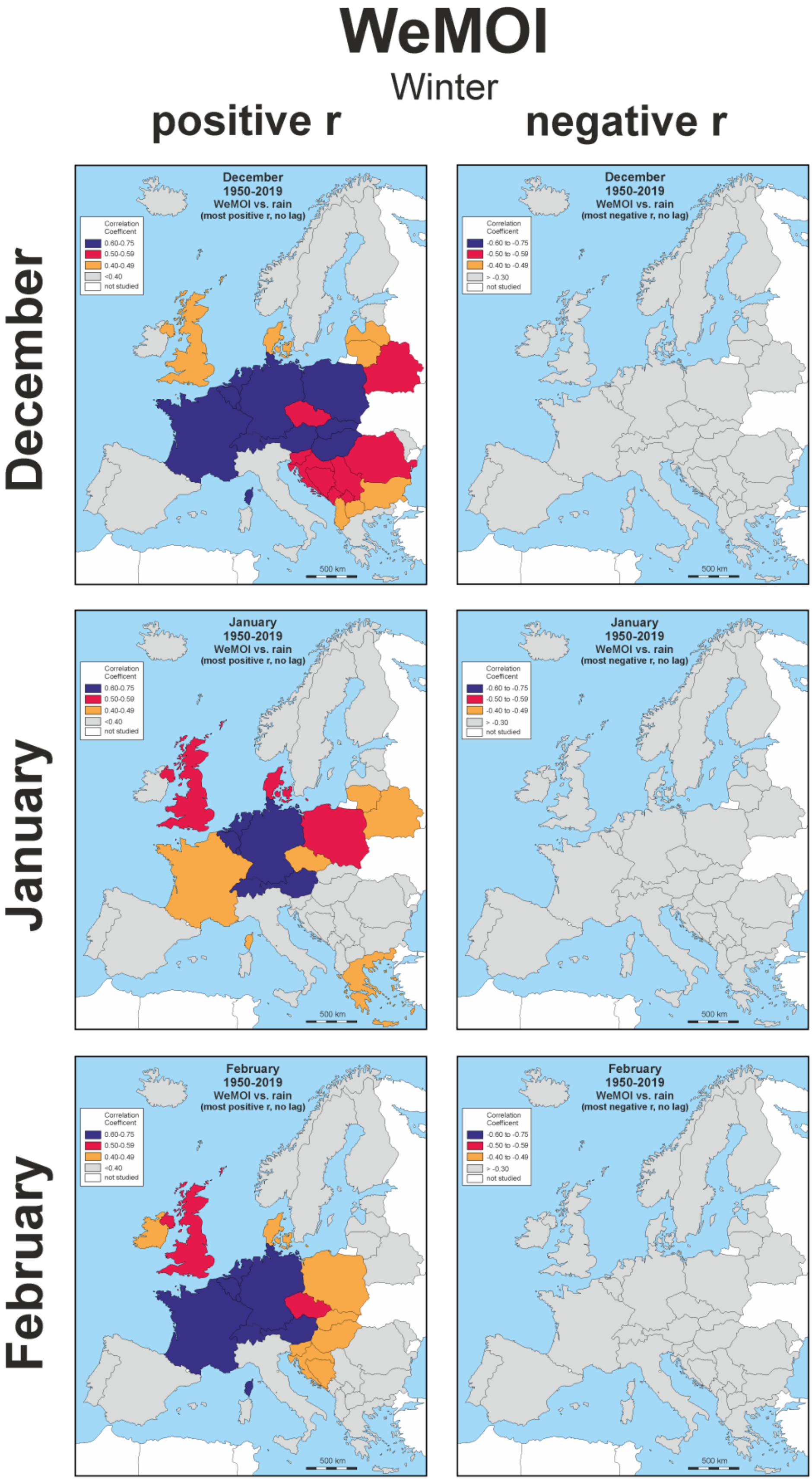

WeMOI

Spring

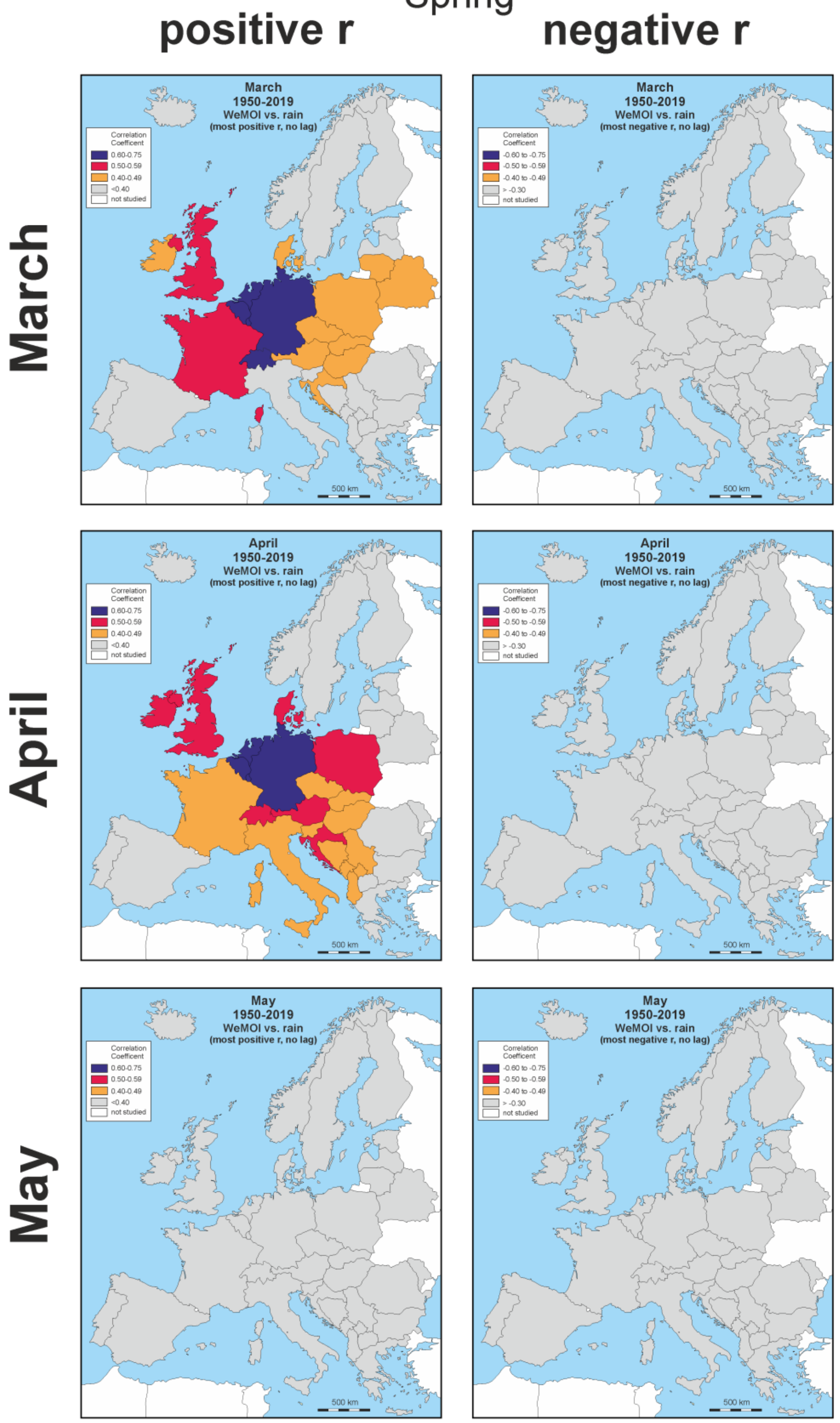

WeMOI

Summer

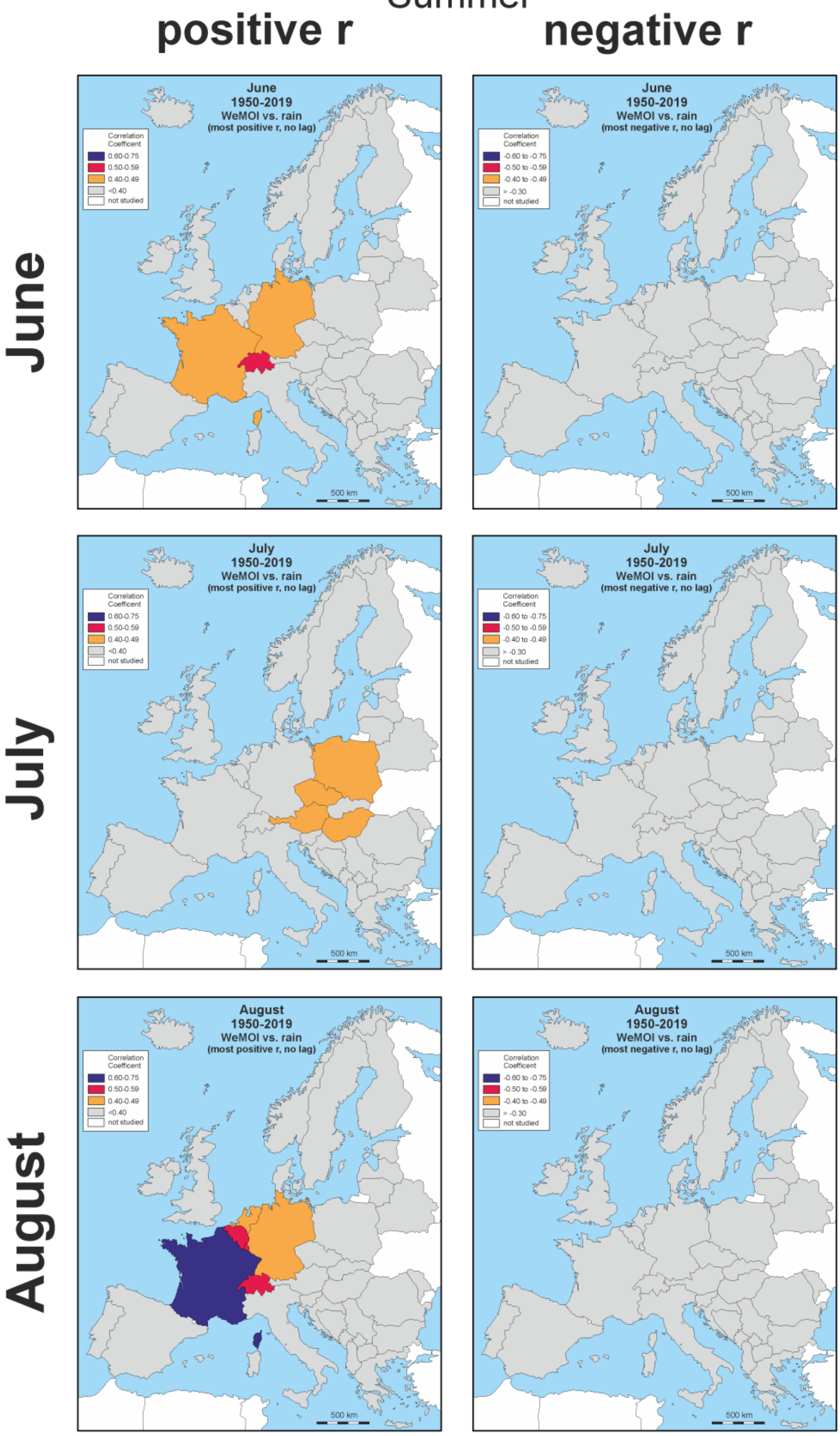

WeMOI

Autumn

positive r **negative r**

September

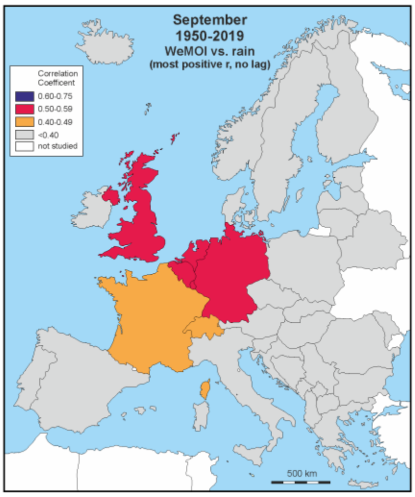

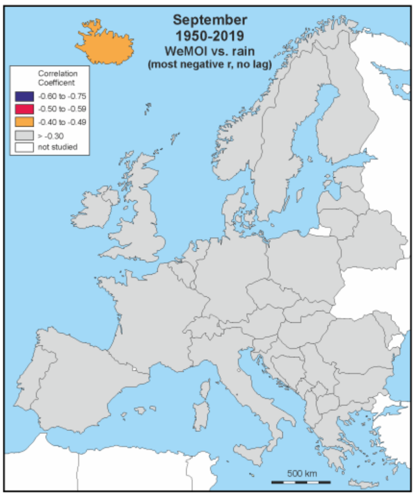

October

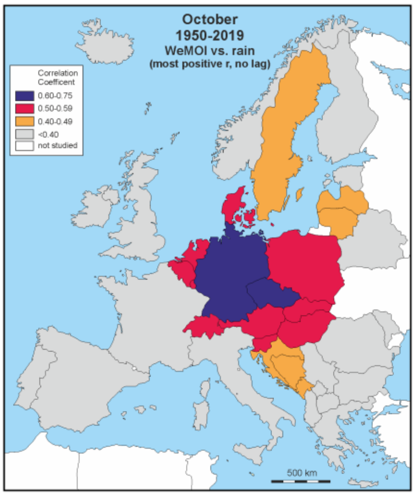

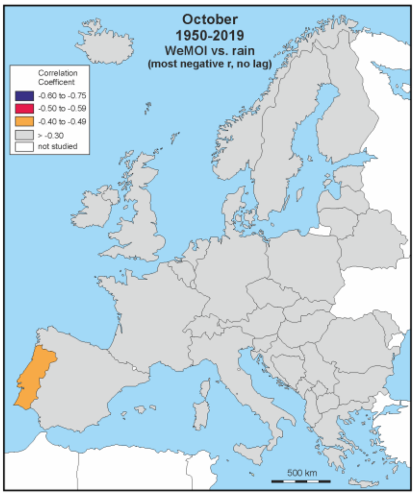

November

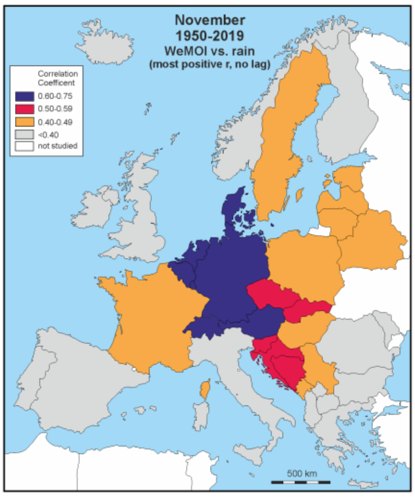

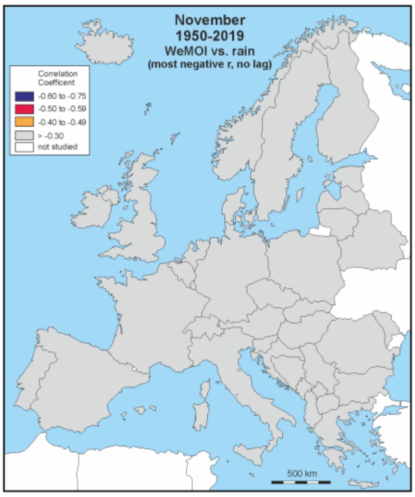

5. Comparative Times Series of Best Correlations (Figure S6)

The graphs visualise the best correlations from the Tables S1-S5 in a time series format. We limited the number of graphs to 32, implying that only correlations equal or larger than |*r*| = 0.72 are shown. One correlation of the NAO, none of the AO, 15 of the NCP, 6 of the MOI2, and 10 of the WeMOI made it into this "first league". To facilitate comparison, both the precipitation and the atmospheric index series are plotted as standard (z) scores, i.e. anomalies from the mean divided by the standard deviation. In case of negative correlation, the atmospheric index is mirrored at z = 0. In the plot titles, the month is given in a numerical format, i.e. 1 = January, …, 12 = December.

(-1)NAO vs Spain precipitation(black), 1950-2019, month = 12, r = -0.74

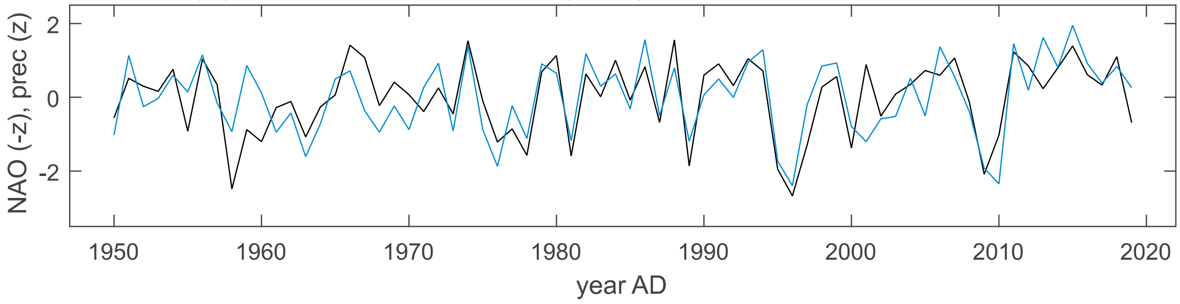

(-1)NCP vs Netherlands precipitation(black), 1950-2019, month = 10, r = -0.79

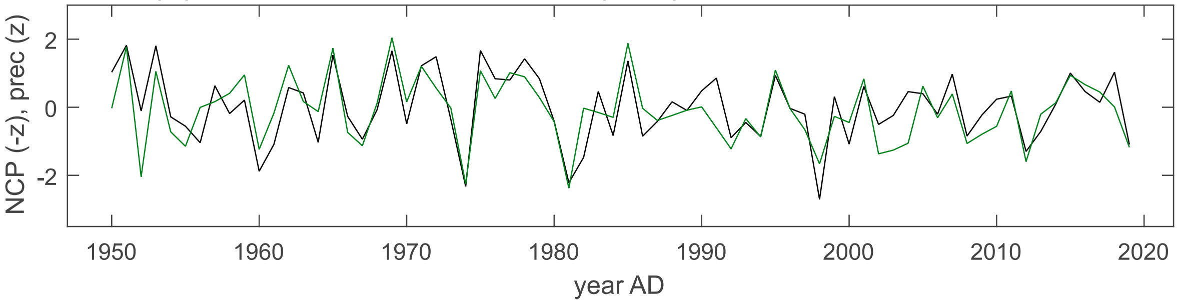

(-1)NCP vs France precipitation(black), 1950-2019, month = 2, r = -0.77

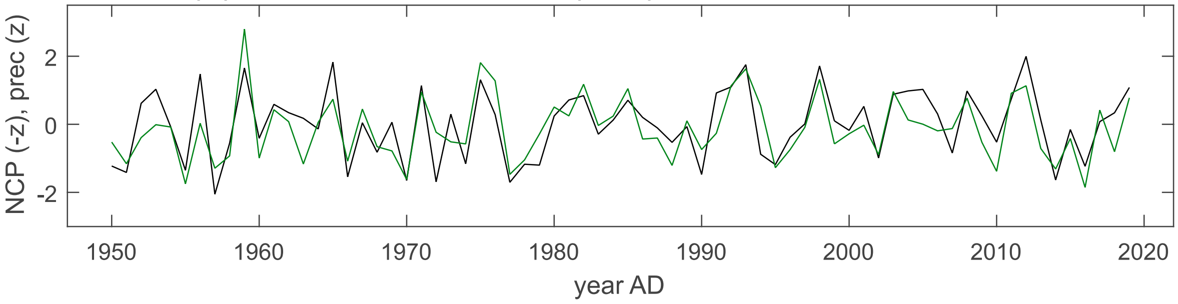

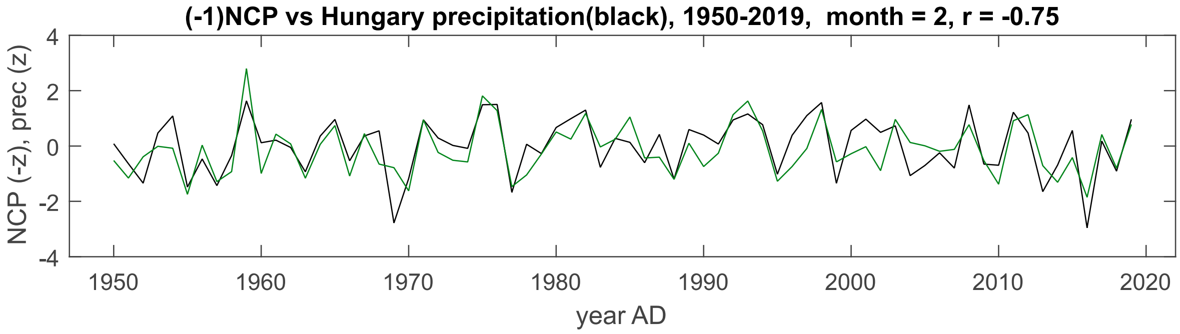
(-1)NCP vs Hungary precipitation(black), 1950-2019, month = 2, r = -0.75
NCP (-z), prec (z)
year AD

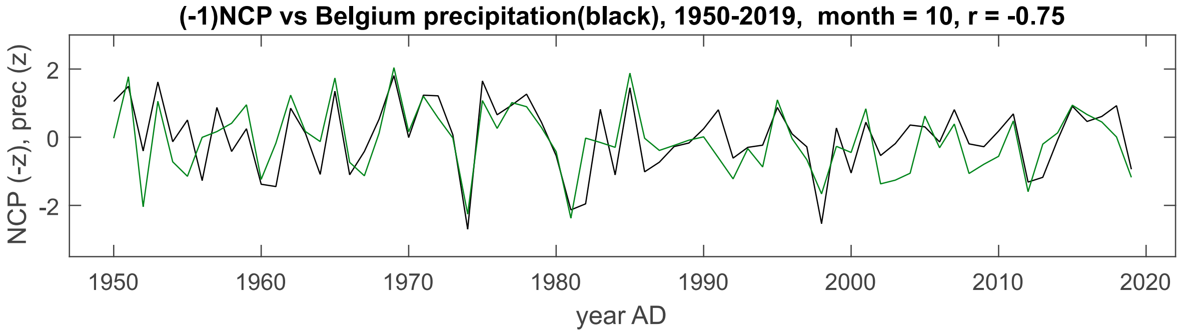
(-1)NCP vs Belgium precipitation(black), 1950-2019, month = 10, r = -0.75
NCP (-z), prec (z)
year AD

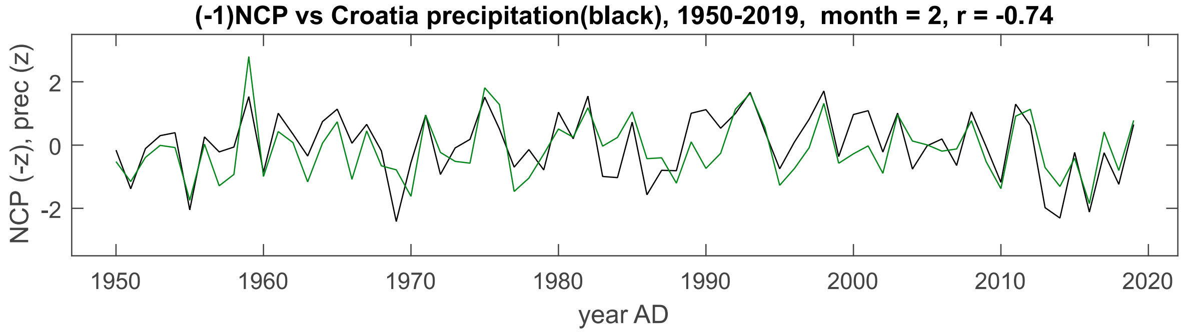
(-1)NCP vs Croatia precipitation(black), 1950-2019, month = 2, r = -0.74
NCP (-z), prec (z)
year AD

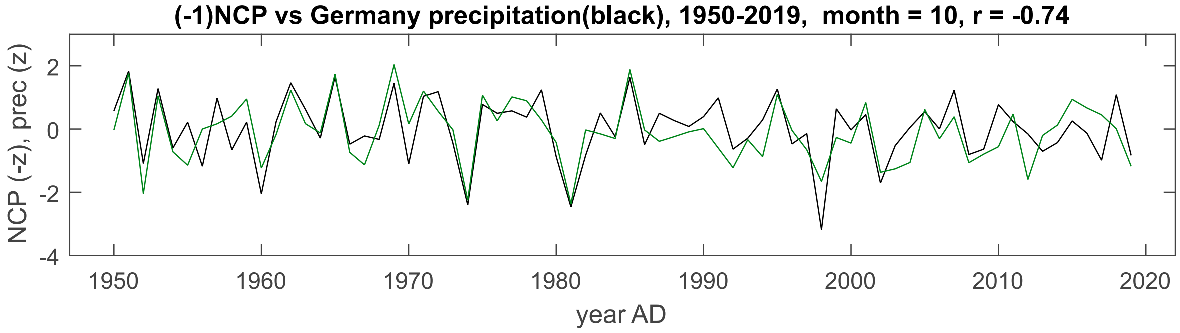
(-1)NCP vs Germany precipitation(black), 1950-2019, month = 10, r = -0.74
NCP (-z), prec (z)
year AD

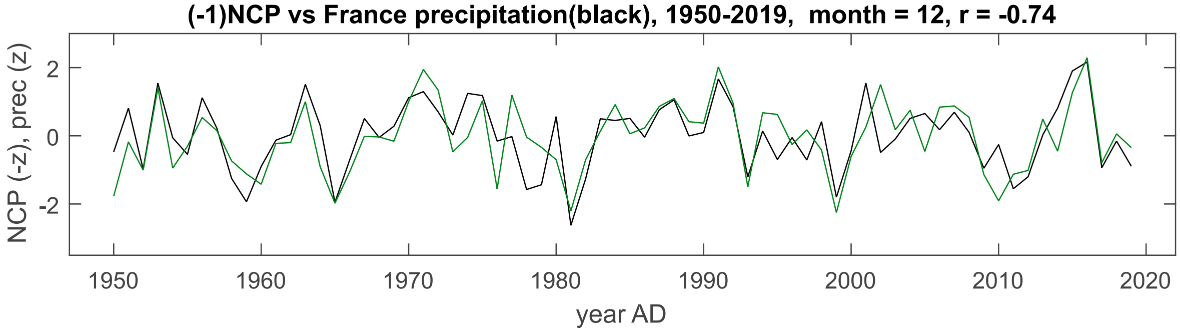
(-1)NCP vs France precipitation(black), 1950-2019, month = 12, r = -0.74
NCP (-z), prec (z)
year AD

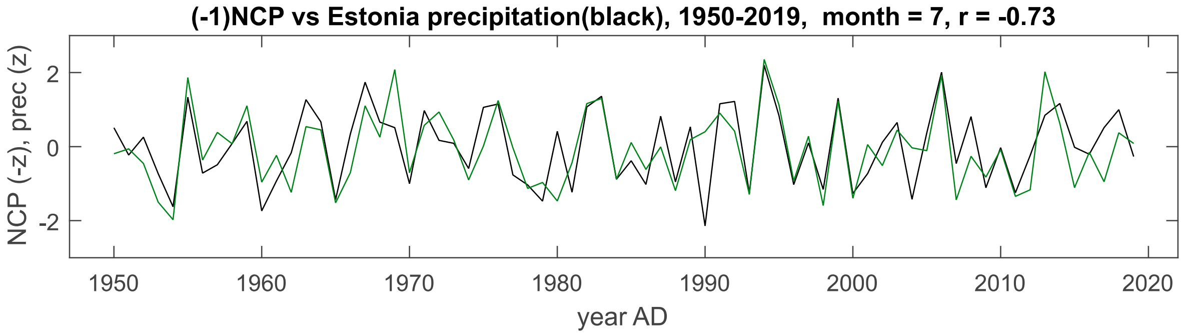
(-1)NCP vs Estonia precipitation(black), 1950-2019, month = 7, r = -0.73
NCP (-z), prec (z)
year AD

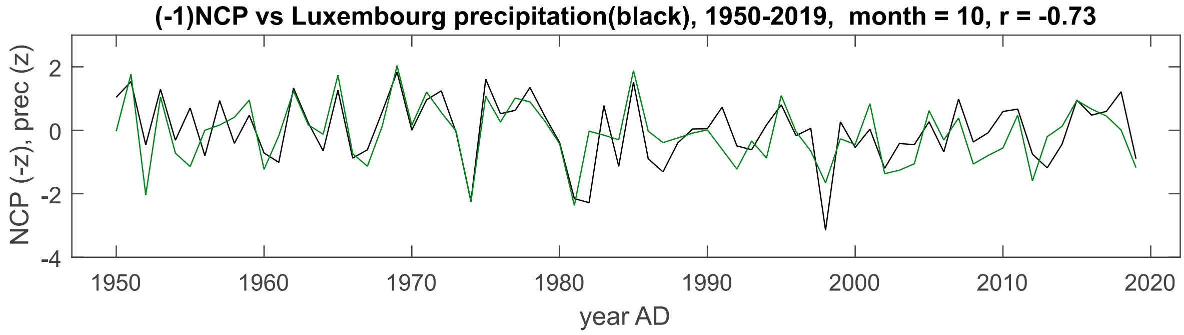
(-1)NCP vs Luxembourg precipitation(black), 1950-2019, month = 10, r = -0.73
NCP (-z), prec (z)
year AD

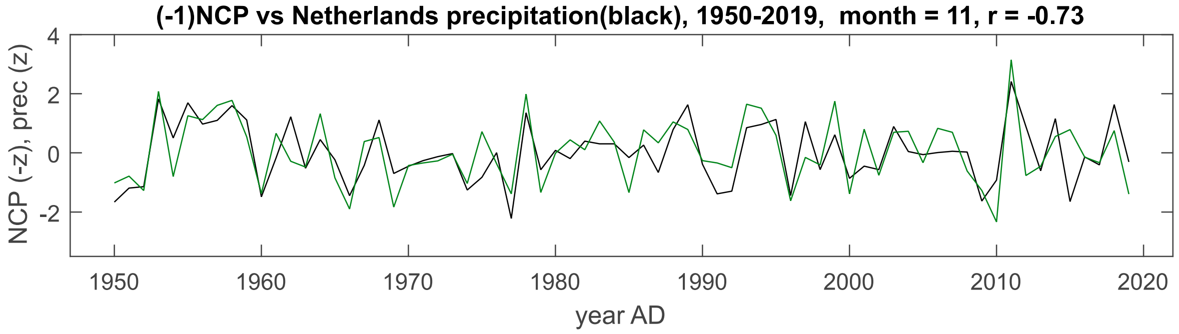
(-1)NCP vs Netherlands precipitation(black), 1950-2019, month = 11, r = -0.73
NCP (-z), prec (z)
year AD

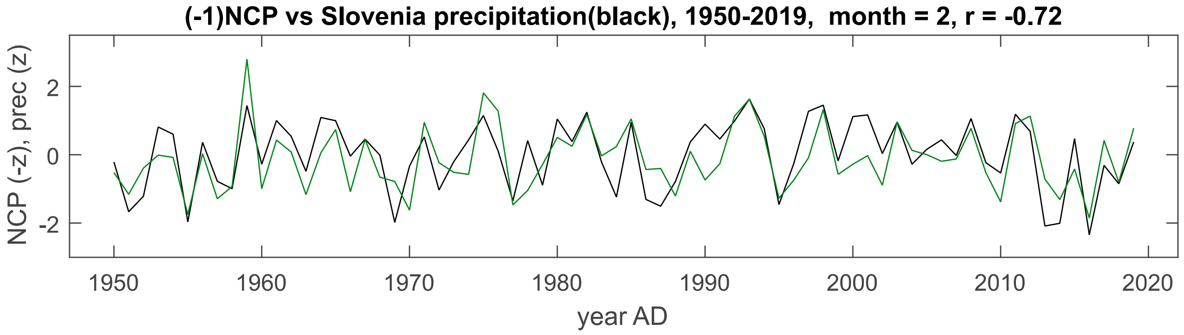
(-1)NCP vs Slovenia precipitation(black), 1950-2019, month = 2, r = -0.72
NCP (-z), prec (z)
year AD

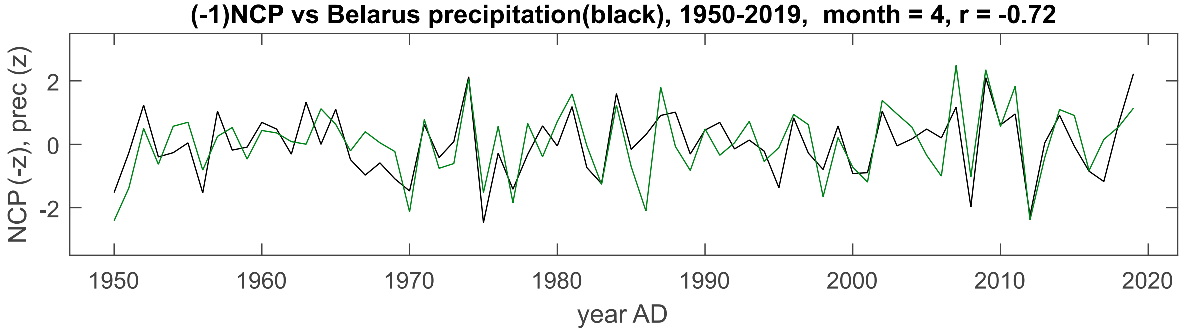
(-1)NCP vs Belarus precipitation(black), 1950-2019, month = 4, r = -0.72
NCP (-z), prec (z)
year AD

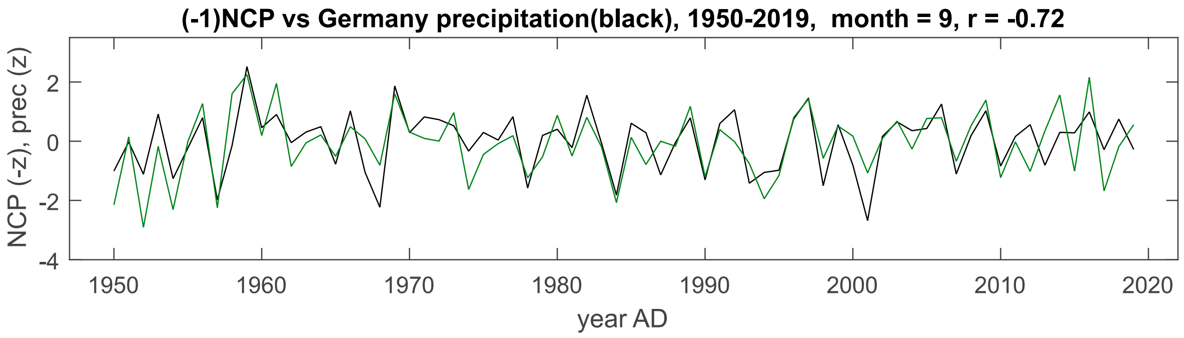
(-1)NCP vs Germany precipitation(black), 1950-2019, month = 9, r = -0.72
NCP (-z), prec (z)
year AD

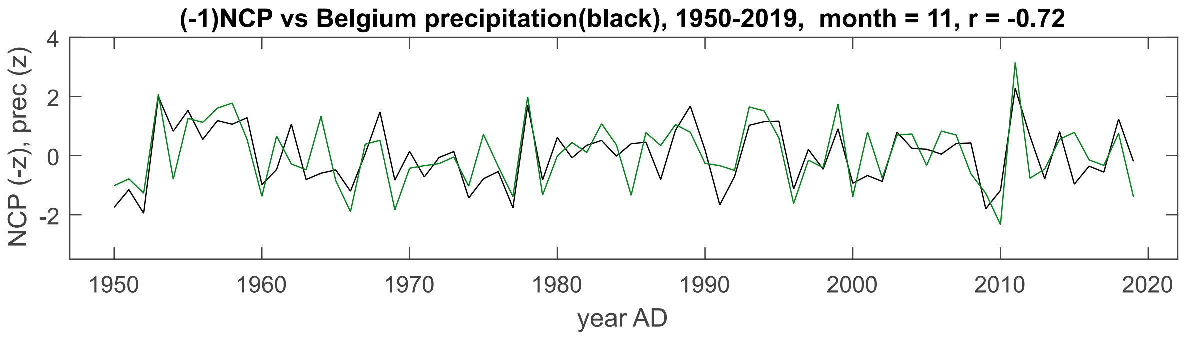
(-1)NCP vs Belgium precipitation(black), 1950-2019, month = 11, r = -0.72
NCP (-z), prec (z)
year AD

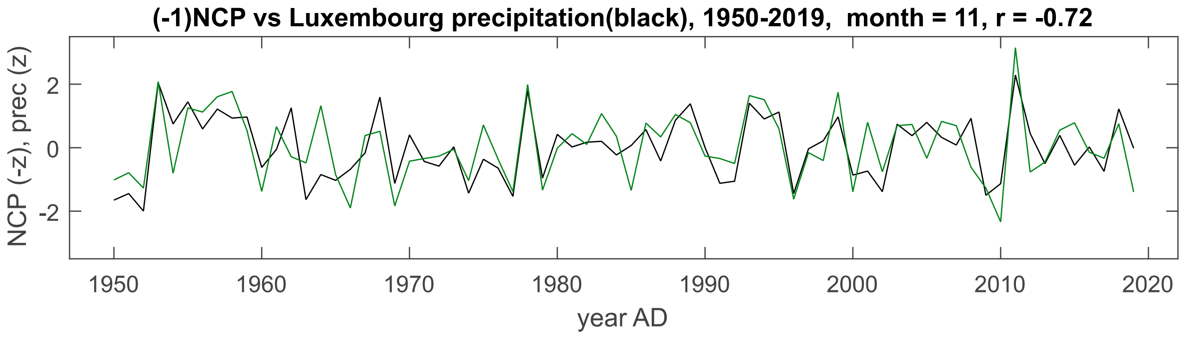
(-1)NCP vs Luxembourg precipitation(black), 1950-2019, month = 11, r = -0.72
NCP (-z), prec (z)
year AD

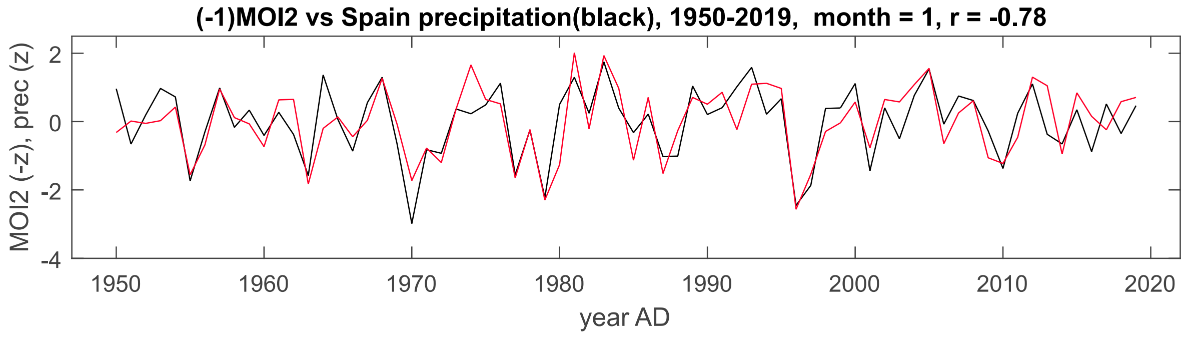
(-1)MOI2 vs Spain precipitation(black), 1950-2019, month = 1, r = -0.78
MOI2 (-z), prec (z)
year AD

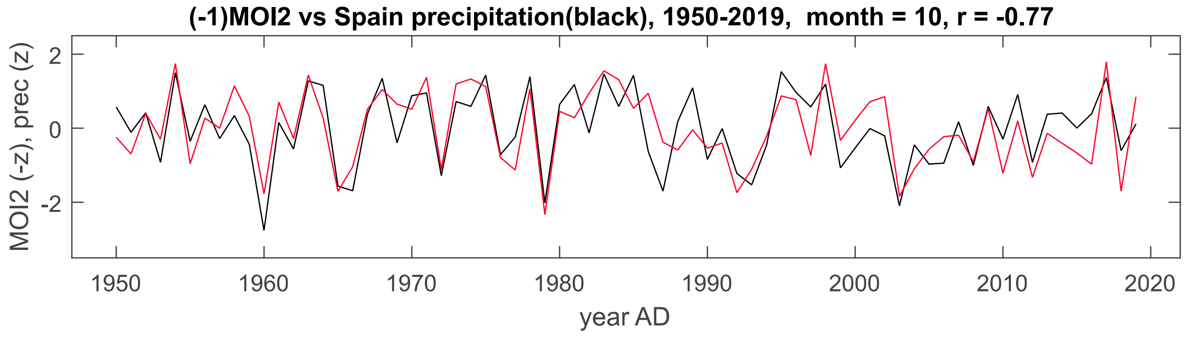
(-1)MOI2 vs Spain precipitation(black), 1950-2019, month = 10, r = -0.77
MOI2 (-z), prec (z)
year AD

(-1)MOI2 vs Spain precipitation(black), 1950-2019, month = 12, r = -0.77

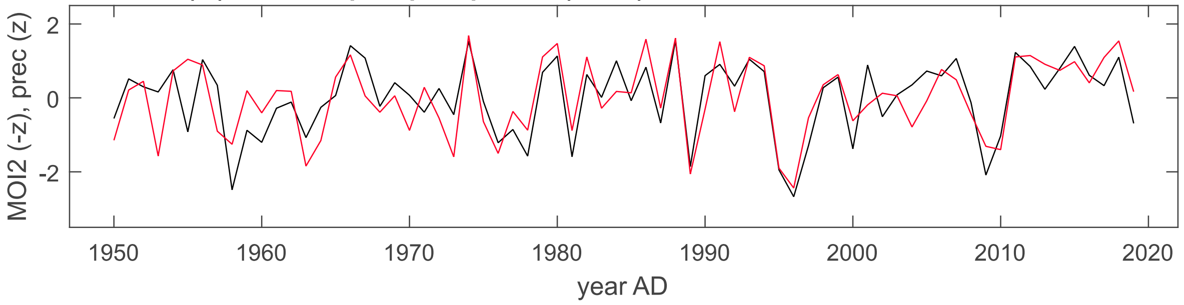

(-1)MOI2 vs Portugal precipitation(black), 1950-2019, month = 1, r = -0.74

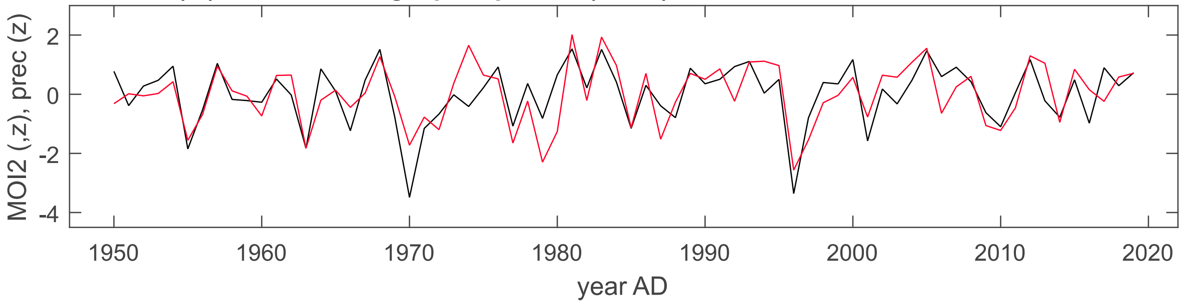

(-1)MOI2 vs Portugal precipitation(black), 1950-2019, month = 10, r = -0.74

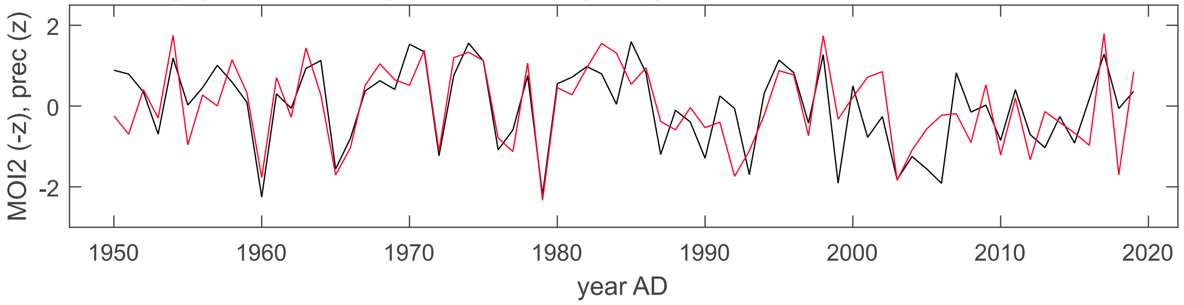

(-1)MOI2 vs Spain precipitation(black), 1950-2019, month = 2, r = -0.72

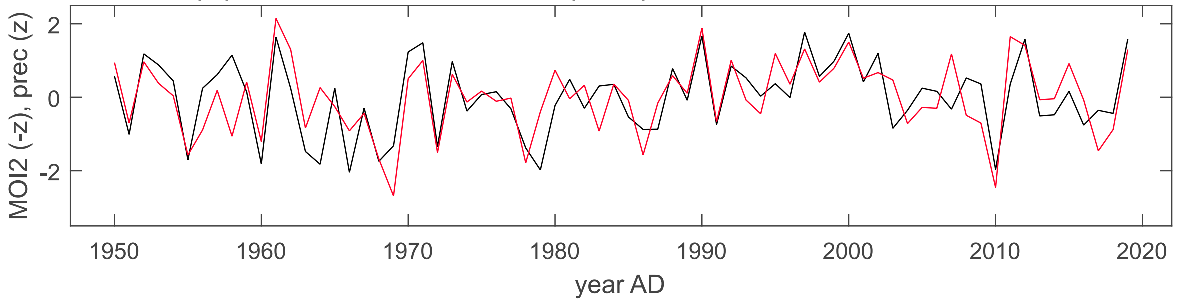

WeMOI vs Germany precipitation(black), 1950-2019, month = 1, r = 0.76

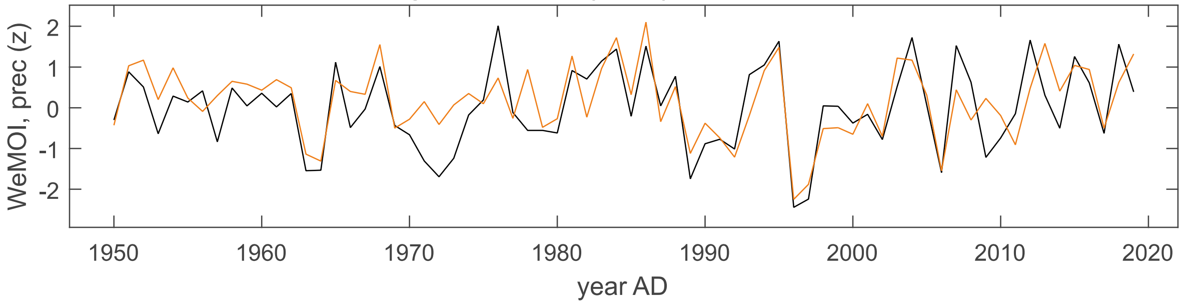

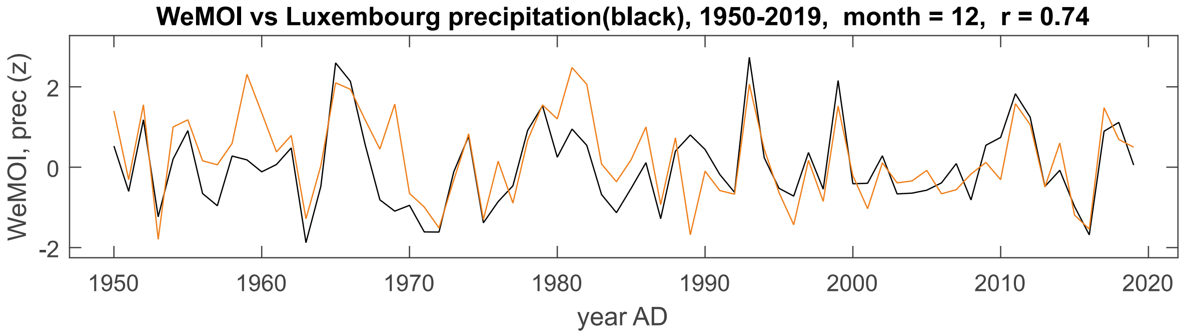
WeMOI vs Luxembourg precipitation(black), 1950-2019, month = 12, r = 0.74
WeMOI, prec (z)
year AD

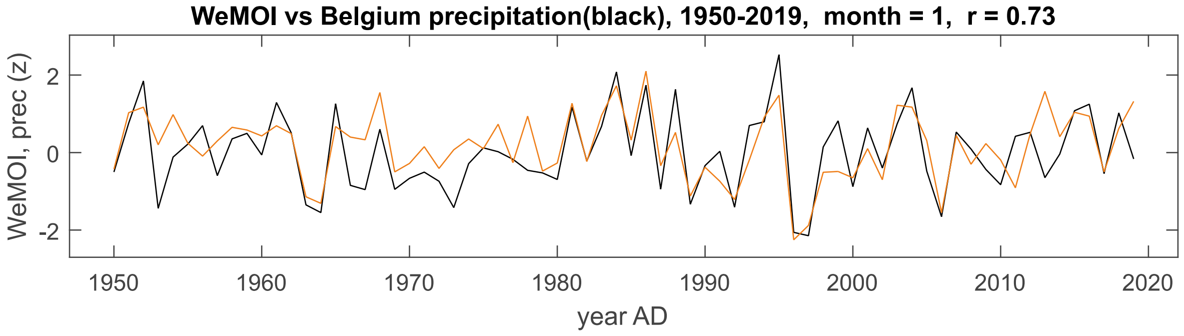
WeMOI vs Belgium precipitation(black), 1950-2019, month = 1, r = 0.73
WeMOI, prec (z)
year AD

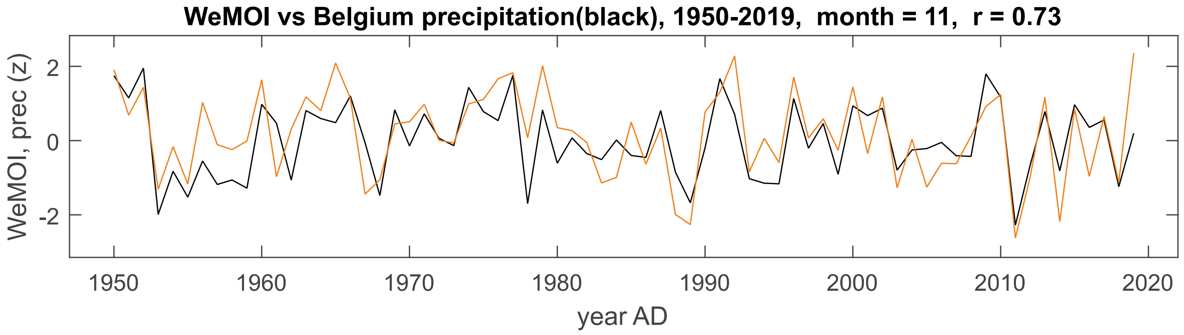
WeMOI vs Belgium precipitation(black), 1950-2019, month = 11, r = 0.73
WeMOI, prec (z)
year AD

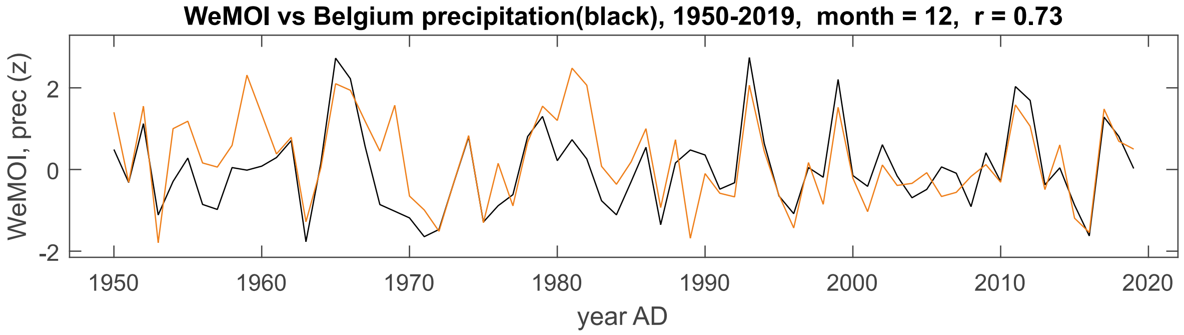
WeMOI vs Belgium precipitation(black), 1950-2019, month = 12, r = 0.73
WeMOI, prec (z)
year AD

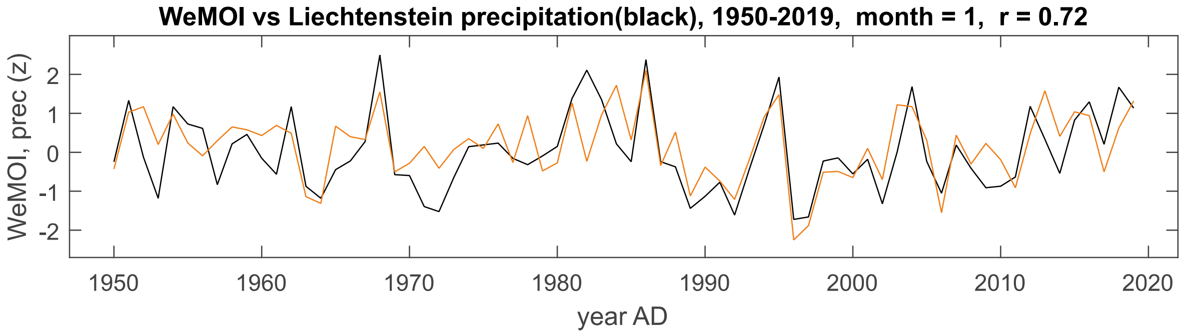
WeMOI vs Liechtenstein precipitation(black), 1950-2019, month = 1, r = 0.72
WeMOI, prec (z)
year AD

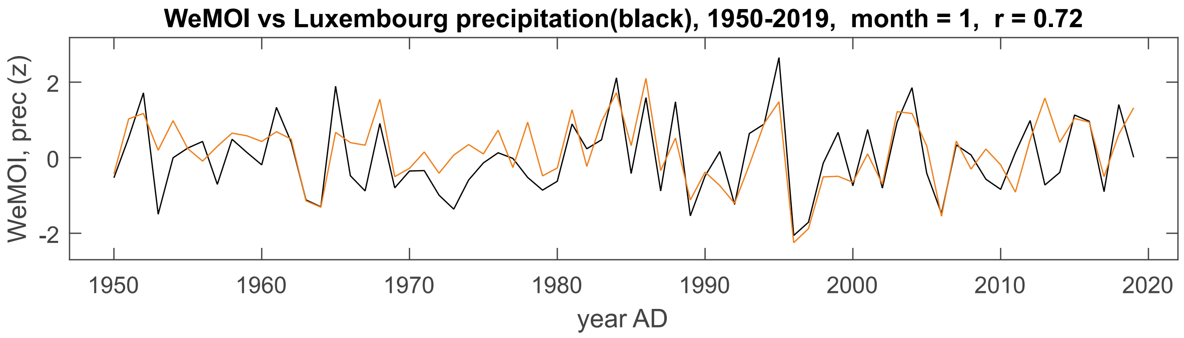
WeMOI vs Luxembourg precipitation(black), 1950-2019, month = 1, r = 0.72
WeMOI, prec (z)
year AD

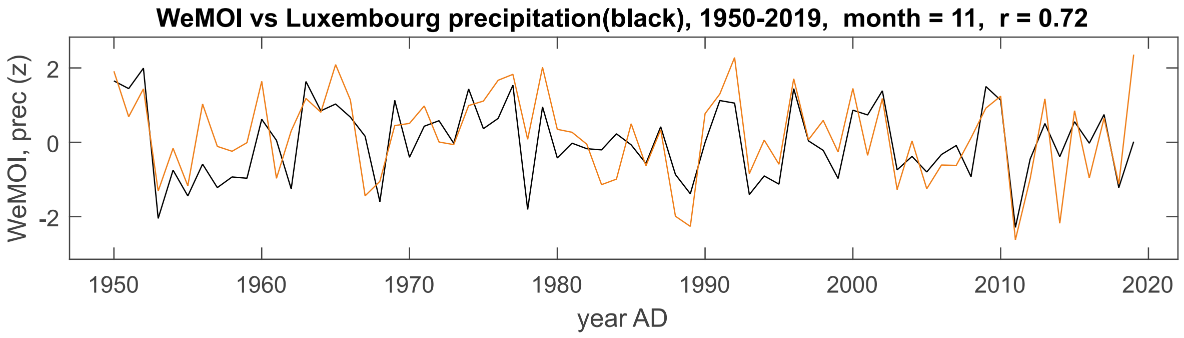
WeMOI vs Luxembourg precipitation(black), 1950-2019, month = 11, r = 0.72
WeMOI, prec (z)
year AD

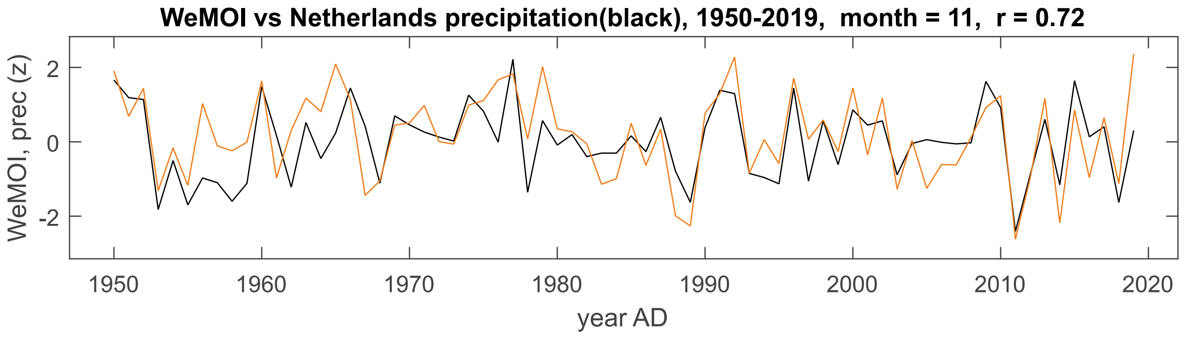
WeMOI vs Netherlands precipitation(black), 1950-2019, month = 11, r = 0.72
WeMOI, prec (z)
year AD

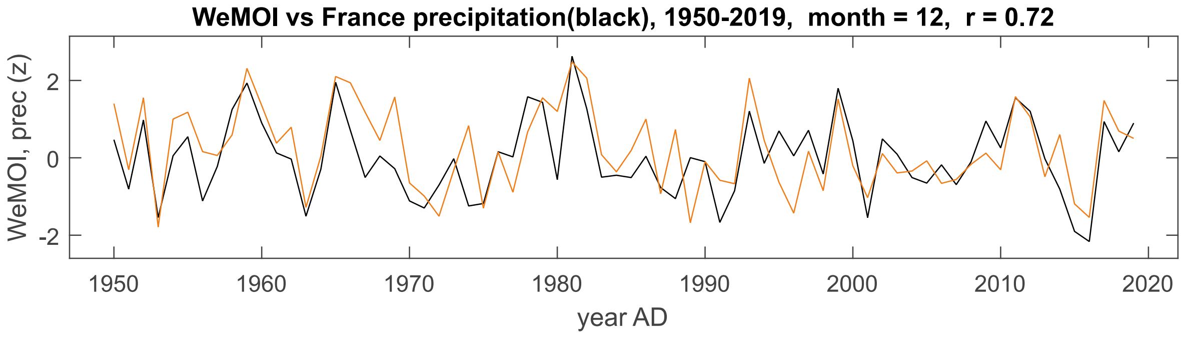
WeMOI vs France precipitation(black), 1950-2019, month = 12, r = 0.72
WeMOI, prec (z)
year AD

6. Evolution of Pearson *r* for all European Countries Through the Year (Figure S7)

The numbers on the abscissa indicate the months.

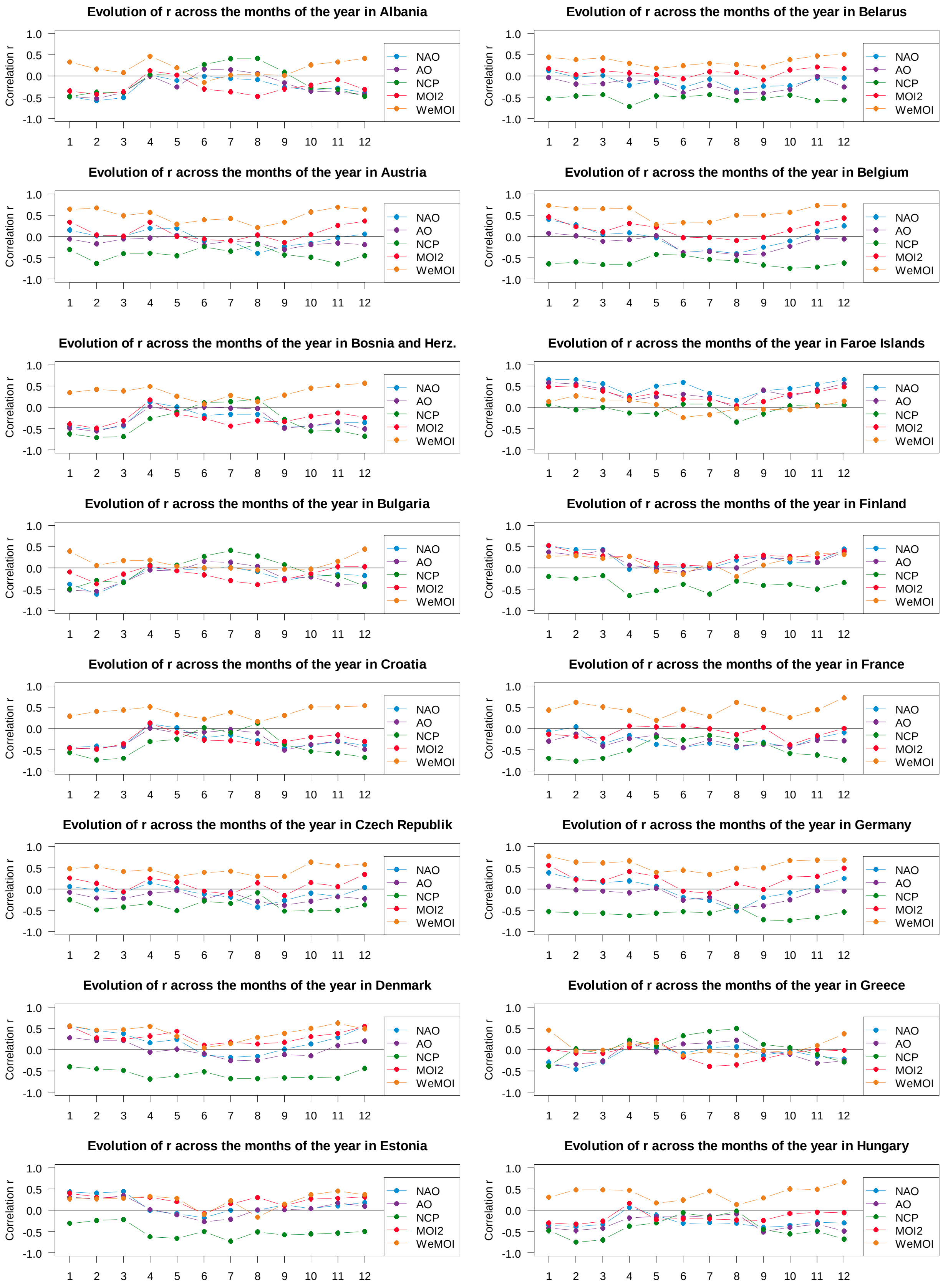

Evolution of r across the months of the year in Iceland

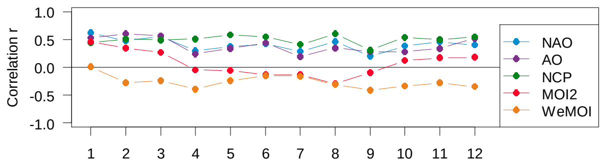

Evolution of r across the months of the year in Ireland

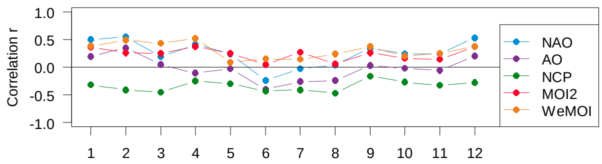

Evolution of r across the months of the year in Italy

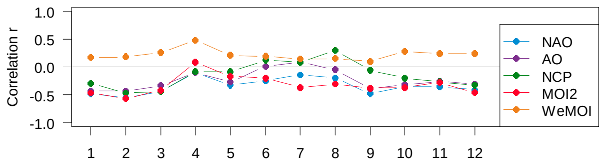

Evolution of r across the months of the year in Kosovo

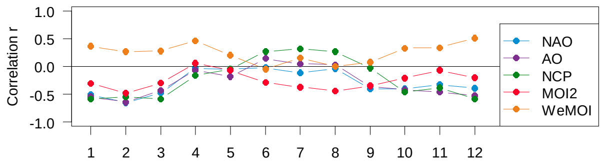

Evolution of r across the months of the year in Latvia

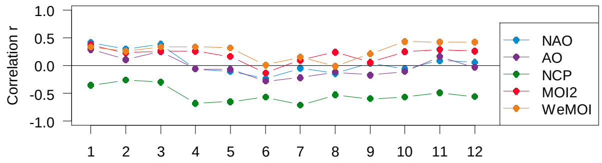

Evolution of r across the months of the year in Liechtenstein

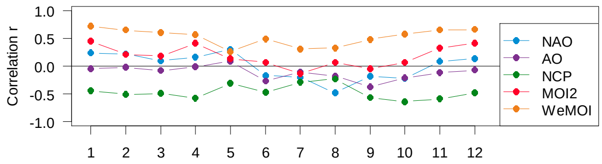

Evolution of r across the months of the year in Lithuania

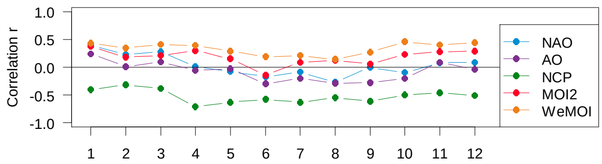

Evolution of r across the months of the year in Luxembourg

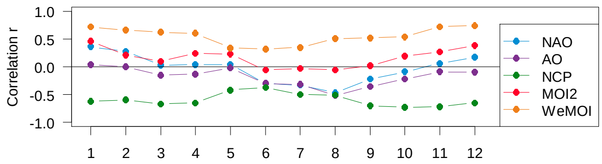

Evolution of r across the months of the year in Makedonia

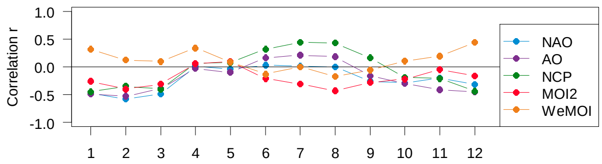

Evolution of r across the months of the year in Moldova

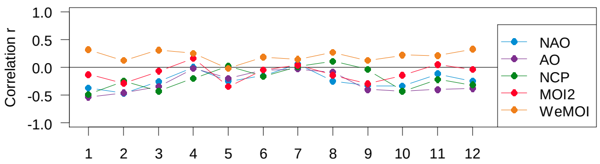

Evolution of r across the months of the year in Montenegro

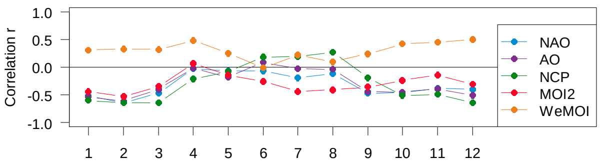

Evolution of r across the months of the year in Netherlands

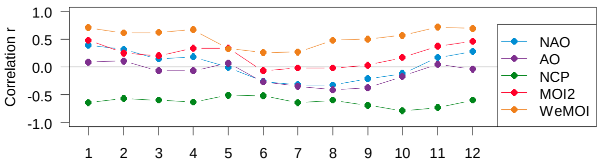

Evolution of r across the months of the year in Norway

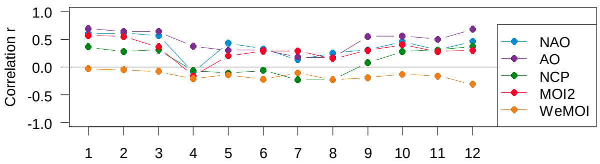

Evolution of r across the months of the year in Poland

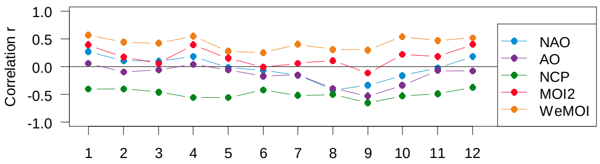

Evolution of r across the months of the year in Portugal

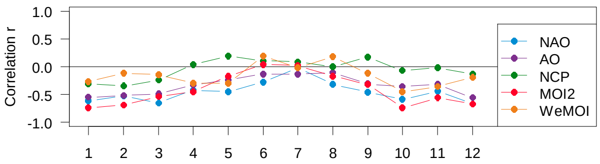

Evolution of r across the months of the year in Romania

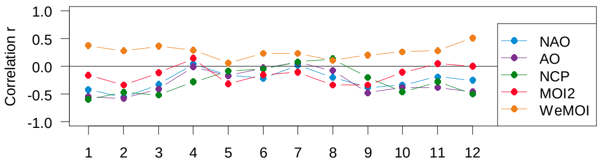

Evolution of r across the months of the year in Serbia

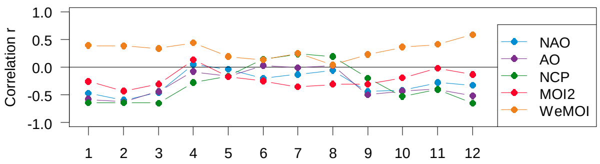

Evolution of r across the months of the year in Slovakia

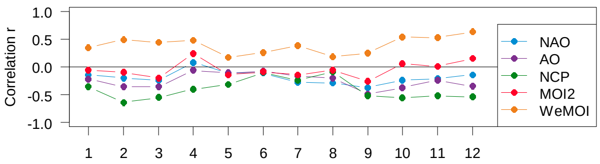

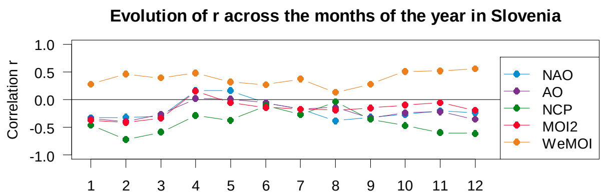
Evolution of r across the months of the year in Slovenia
Correlation r
1.0
0.5
0.0
-0.5
-1.0
1 2 3 4 5 6 7 8 9 10 11 12
NAO
AO
NCP
MOI2
WeMOI

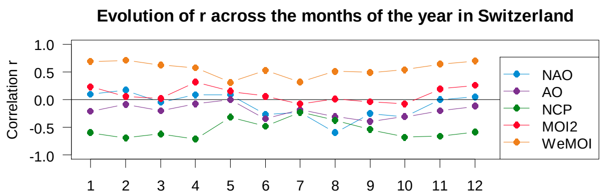
Evolution of r across the months of the year in Switzerland
Correlation r
1.0
0.5
0.0
-0.5
-1.0
1 2 3 4 5 6 7 8 9 10 11 12
NAO
AO
NCP
MOI2
WeMOI

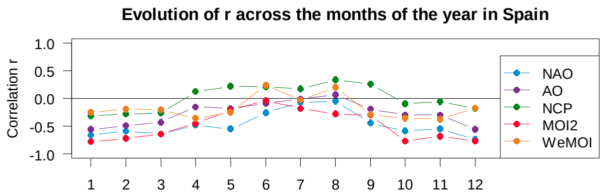
Evolution of r across the months of the year in Spain
Correlation r
1.0
0.5
0.0
-0.5
-1.0
1 2 3 4 5 6 7 8 9 10 11 12
NAO
AO
NCP
MOI2
WeMOI

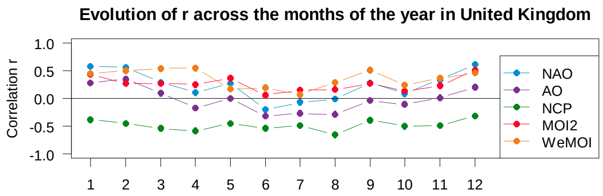
Evolution of r across the months of the year in United Kingdom
Correlation r
1.0
0.5
0.0
-0.5
-1.0
1 2 3 4 5 6 7 8 9 10 11 12
NAO
AO
NCP
MOI2
WeMOI

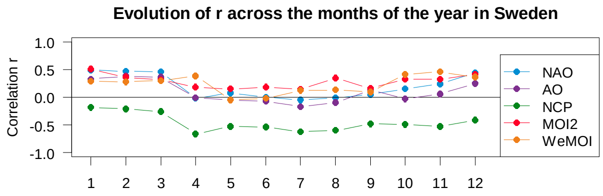
Evolution of r across the months of the year in Sweden
Correlation r
1.0
0.5
0.0
-0.5
-1.0
1 2 3 4 5 6 7 8 9 10 11 12
NAO
AO
NCP
MOI2
WeMOI

7. Intercorrelation of Atmospheric Indices (Potential Drivers) for All Months

Table S6. Pairwise correlation (Pearson *r* values) of the monthly indices of the five atmospheric indices across the years 1950-2019. Significant values are marked with * ($p < 0.05$), ** ($p < 0.01$), *** ($p < 0.001$), without correction for multiple testing.

January

	NAO	AO	NCP	MOI2	WeMOI
NAO	1	0.79***	0.18	0.83***	0.37**
AO	0.79***	1	0.38**	0.55***	0.02
NCP	0.18	0.38**	1	0.06	-0.61***
MOI2	0.83***	0.55***	0.06	1	0.55***
WeMOI	0.37**	0.02	-0.61***	0.55***	1

February

	NAO	AO	NCP	MOI2	WeMOI
NAO	1	0.85***	0.23	0.76***	0.21
AO	0.85***	1	0.39***	0.64***	-0.01
NCP	0.23	0.39***	1	0.30*	-0.66***
MOI2	0.76***	0.64***	0.30*	1	0.33**
WeMOI	0.21	-0.01	-0.66***	0.33**	1

March

	NAO	AO	NCP	MOI2	WeMOI
NAO	1	0.82***	0.35**	0.77***	0.28*
AO	0.82***	1	0.38**	0.48***	0.00
NCP	0.35**	0.38**	1	0.24*	-0.48***
MOI2	0.77***	0.48***	0.24*	1	0.46***
WeMOI	0.28*	0.00	-0.48***	0.46***	1

April

	NAO	AO	NCP	MOI2	WeMOI
NAO	1	0.41***	0.14	0.48***	0.28*
AO	0.41***	1	0.18	0.21	-0.09
NCP	0.14	0.18	1	-0.21	-0.50***
MOI2	0.48***	0.21	-0.21	1	0.63***
WeMOI	0.28*	-0.09	-0.50***	0.63***	1

May

	NAO	AO	NCP	MOI2	WeMOI
NAO	1	0.57***	0.09	0.43***	0.21
AO	0.57***	1	0.23	0.28*	-0.01
NCP	0.09	0.23	1	-0.20	-0.35**
MOI2	0.43***	0.28*	-0.20	1	0.48***
WeMOI	0.21	-0.01	-0.35**	0.48***	1

June

	NAO	AO	NCP	MOI2	WeMOI
NAO	1	0.64***	0.24*	0.33**	0.00
AO	0.64***	1	0.42***	0.09	-0.22
NCP	0.24*	0.42***	1	-0.02	-0.20
MOI2	0.33**	0.09	-0.02	1	0.40***
WeMOI	0.00	-0.22	-0.20	0.40***	1

July

	NAO	AO	NCP	MOI2	WeMOI
NAO	1	0.53***	0.22	0.30*	-0.18
AO	0.53***	1	0.20	0.17	-0.09
NCP	0.22	0.20	1	-0.13	-0.29*
MOI2	0.30*	0.17	-0.13	1	0.22
WeMOI	-0.18	-0.09	-0.29*	0.22	1

August

	NAO	AO	NCP	MOI2	WeMOI
NAO	1	0.53***	0.29*	0.24*	-0.29*
AO	0.53***	1	0.27*	0.14	-0.21
NCP	0.29*	0.27*	1	-0.06	-0.26*
MOI2	0.24*	0.14	-0.06	1	0.13
WeMOI	-0.29*	-0.21	-0.26*	0.13	1

September

	NAO	AO	NCP	MOI2	WeMOI
NAO	1	0.54***	0.13	0.62***	-0.20
AO	0.54***	1	0.31**	0.31**	-0.26*
NCP	0.13	0.31**	1	-0.09	-0.52***
MOI2	0.62***	0.31**	-0.09	1	0.39***
WeMOI	-0.20	-0.26*	-0.52***	0.39***	1

October

	NAO	AO	NCP	MOI2	WeMOI
NAO	1	0.63***	0.36**	0.71***	0.21
AO	0.63***	1	0.32**	0.37**	-0.04
NCP	0.36**	0.32**	1	0.04	-0.52***
MOI2	0.71***	0.37**	0.04	1	0.51***
WeMOI	0.21	-0.04	-0.52***	0.51***	1

November

	NAO	AO	NCP	MOI2	WeMOI
NAO	1	0.56***	0.19	0.78***	0.26*
AO	0.56***	1	0.29*	0.41***	-0.06
NCP	0.19	0.29*	1	-0.06	-0.66***
MOI2	0.78***	0.41***	-0.06	1	0.56***
WeMOI	0.26*	-0.06	-0.66***	0.56***	1

December

	NAO	AO	NCP	MOI2	WeMOI
NAO	1	0.74***	0.17	0.81***	0.24*
AO	0.74***	1	0.41***	0.45***	-0.15
NCP	0.17	0.41***	1	0.03	-0.72***
MOI2	0.81***	0.45***	0.03	1	0.45***
WeMOI	0.24*	-0.15	-0.72***	0.45***	1

8. Best Subset Regression: Fit and Validation for Exemplary Regions and Months.

Leading Tables S7-S9 and detailed Figures S8-S10 for three selected European regions and months.

Caption for Tables: Best subset regression for geographical adjacent countries in a selected month. Shown are the estimated coefficients in the regression equation $\hat{Y} = \hat{\alpha} + \hat{\beta}_1 X_1 + \cdots + \hat{\beta}_5 X_5$, with $\hat{Y}$ denoting the modelled rainfall and $X_{1,\dots,} X_5$ the five atmospheric indices, fitted to the data of the years 1950-2009 ("fit sample", see Methods). The slash indicates that the respective atmospheric index X_j was dispensable according to the best subset criterion. The last two columns show the correlation between predicted and observed rainfall in the fit sample (1950-2009) and the validation sample (2010-2019).

Caption for Figures: Best subset regression and validation in time series format. The black line represents the actual rainfall 1950-2019 - average marked by the horizontal. In the left part of the graph (1950-2009), the blue line shows the rainfall $\hat{\mathrm{Y}}$ as estimated by the regression equation. The right part illustrates the validation in the years 2010-2019 with the red line as the predicted rainfall that resulted from inserting the atmospheric indices observed in 2010-2019 into the regression equation obtained from 1950-2009. The pink area is the 70% confidence interval of prediction PI (see Statistical Methods in Suppl. ch. 2.2).

Table S7 and Figure S8: January rainfall in Balkan coast countries

Country	Intercept	NAO	AO	NCP	MOI2	WeMOI	r (fit)	r (val)
Slovenia	49.5	/	/	/	-40.9	+22.2	0.67	0.80
Croatia	71.6	/	/	/	-56.4	+29.9	0.79	0.90
Bosnia-Herz.	68.9	/	/	-8.7	-40.1	+19.7	0.78	0.80
Montenegro	98.9	-7.2	/	/	-54.3	+39.5	0.81	0.83
Albania	101.4	-9.3	/	/	-38.2	+38.0	0.77	0.63
N Macedonia	46.3	-7.7	/	/	/	+12.8	0.73	0.46
Greece	97.3	-16.2	/	/	+22.6	+24.4	0.71	0.65

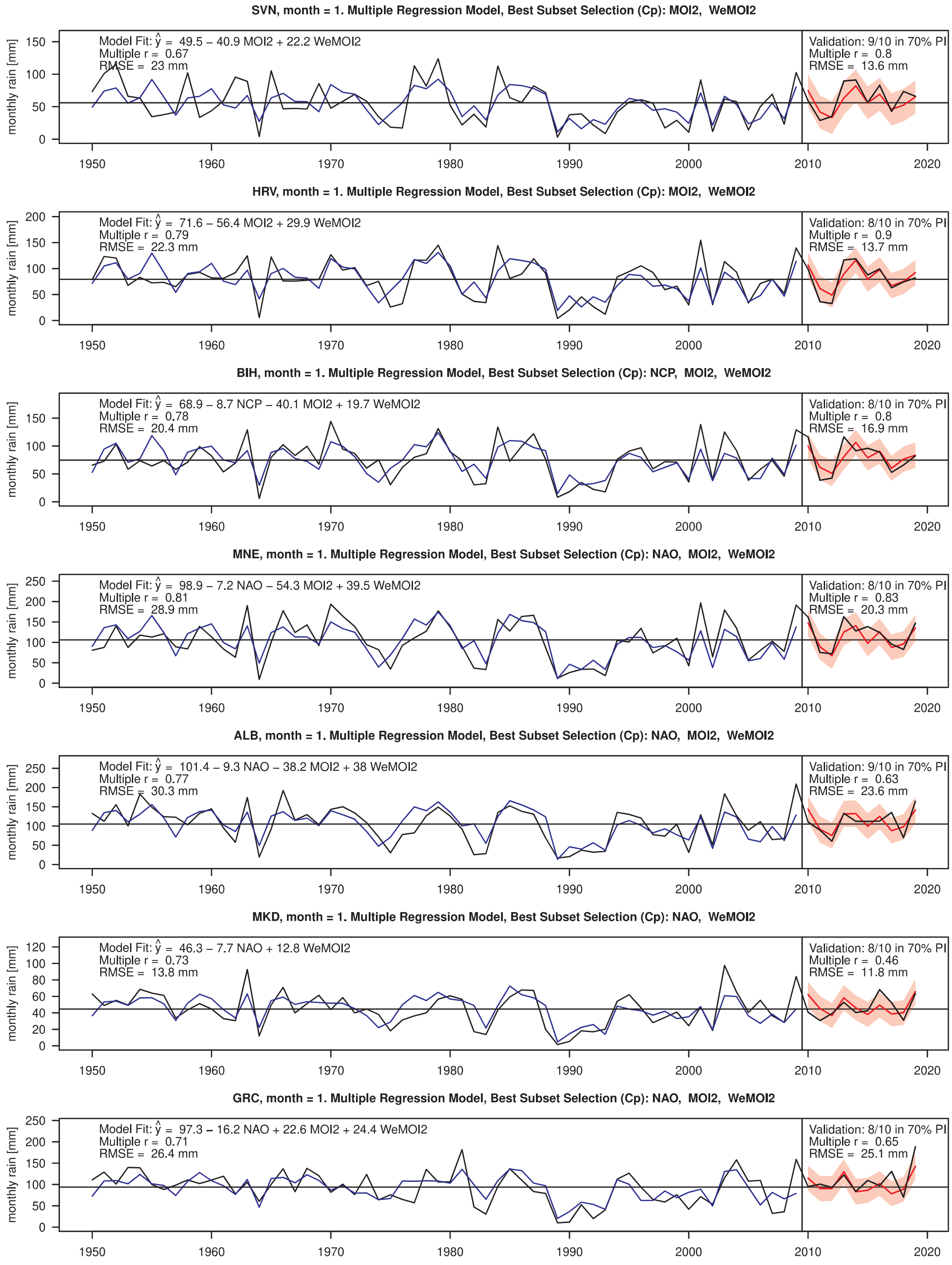

Table S8 and Figure S9: March rainfall in Atlantic coast countries

Country	Intercept	NAO	AO	NCP	MOI2	WeMOI	r (fit)	r (val)
Portugal	89.7	-19.1	/	/	/	/	0.59	0.92
Spain	51.9	-6.2	/	/	-21.6	/	0.62	0.92
France	64.5	-5.4	/	-9.6	/	+11.9	0.77	0.88
Great Britain	91.0	+13.9	-5.7	-17.9	/	/	0.74	0.89
Ireland	84.4	+7.1	/	-15.3	/	/	0.54	0.89
Faroe Islands	133.4	+18.1	/	-10.8	/	/	0.60	0.58
Iceland	79.0	+10.4	/	/	/	-11.1	0.67	0.77

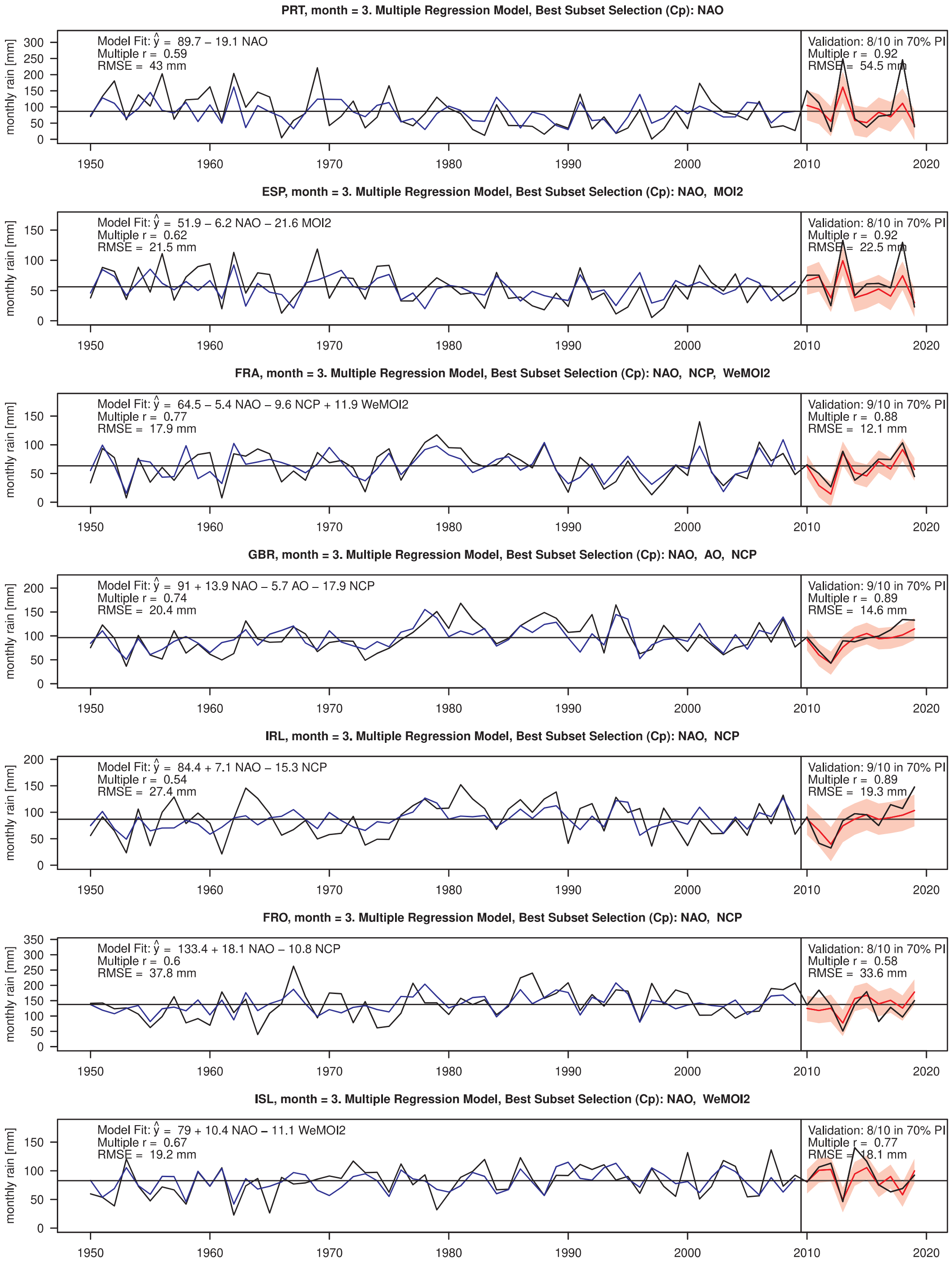

Table S9 and Figure S10: August rainfall in Baltic coast countries

Country	Intercept	NAO	AO	NCP	MOI2	WeMOI	r (fit)	r (val)
Germany	60.4	-3.2	-8.2	/	+23.4	/	0.70	0.72
Poland	65.6	/	-13.6	-6.8	/	/	0.60	0.47
Lithuania	70.8	/	-11.8	-13.5	/	/	0.56	0.65
Latvia	48.9	/	/	-15.2	+46.8	-6.3	0.55	0.83
Estonia	41.2	/	/	-17.5	+71.6	-13.3	0.64	0.84
Finland	48.3	/	/	-8.6	+44.9	-9.5	0.55	0.61
Sweden	51.2	/	/	-10.9	+46.2	/	0.70	0.47

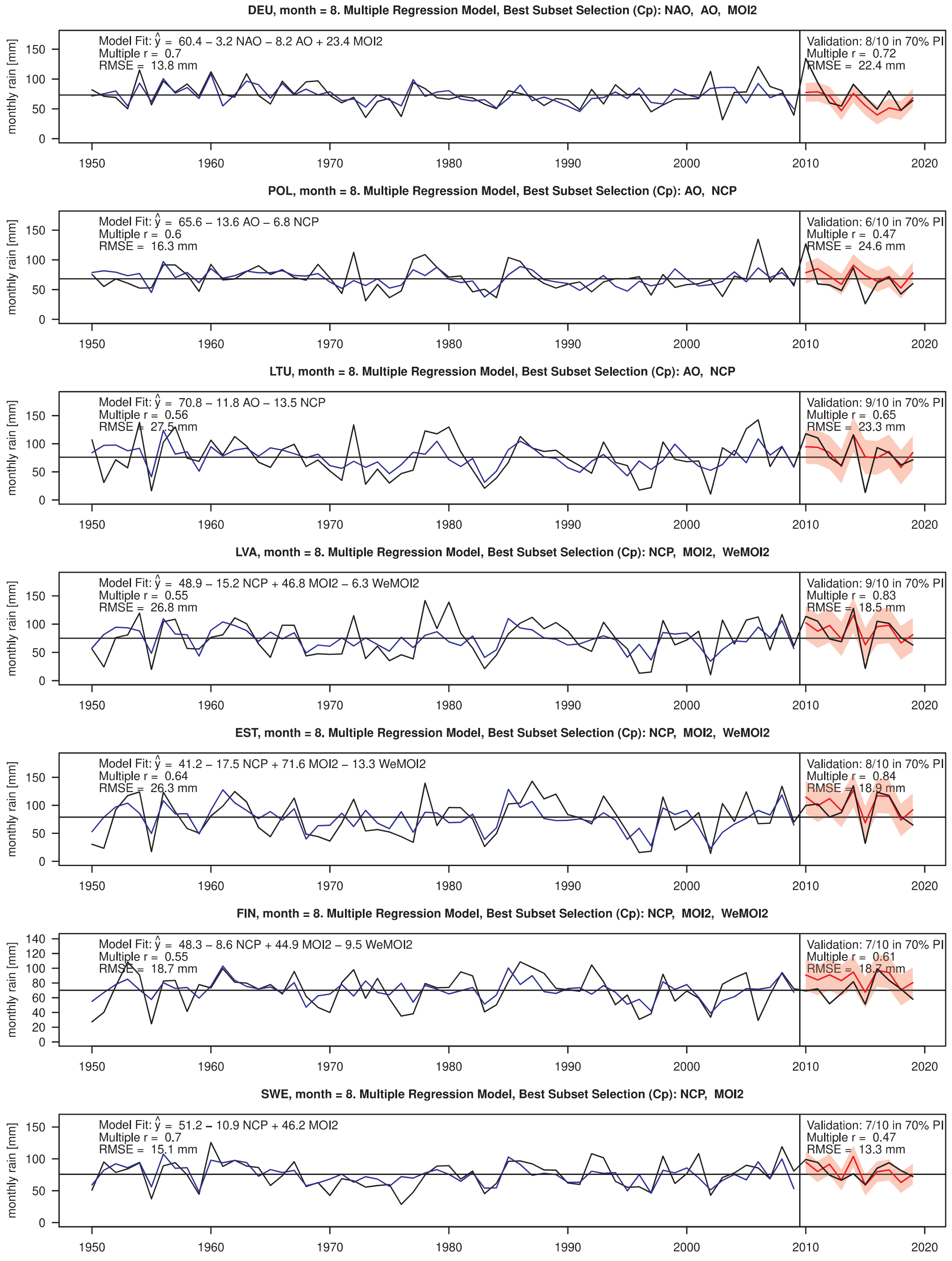

9. Exemplary Pearson Correlation Coefficients for Gridded Precipitation

This chapter contains tables with Pearson correlation coefficients *r* between rainfall and the potential drivers NAO, AO, NCP, MOI2 and WeMOI for seven exemplary grid cells of 1° by 1° size, distributed across three European countries (France, Germany, Italy), for all months of the year for the period 1950-2018. (Unfortunately, the full length of the data series up to 2019 was not yet available for the gridded precipitation data at the time of study.) To facilitate comparison with the country data, these are repeated form the Tables S1-S5 below each table. Statistical significances were not computed because of the arbitrary and sparse selection of grid cells.

The grids are located as follows:
France North: 49°-50° N, 2°-3° E; South: 44°-45° N, 2°-3° E
Germany North: 53°-54° N, 9°-10° E; South: 48°-49° N, 9°-10° E
Italy North: 44°-45° N, 11°-12° E; Central: 41-42 N, 14-15 E; South: 37°-38° N, 14°-15° E.

9.1 NAO vs. Precipitation (Table S10)

Grid data (1950-2018)

	Jan	Feb	Mar	Apr	May	Jun	Jul	Aug	Sept	Oct	Nov	Dec
**France North**	0.29	0.22	-0.10	-0.03	-0.16	-0.47	-0.41	-0.49	-0.19	-0.21	0.05	0.22
**France South**	-0.33	-0.18	-0.46	-0.20	-0.42	-0.27	-0.16	-0.27	-0.29	-0.47	-0.35	-0.37
**Germany North**	0.50	0.38	0.30	0.28	0.03	-0.13	-0.24	-0.26	-0.08	0.02	0.17	0.43
**Germany South**	0.15	0.12	-0.01	0.05	0.09	-0.27	-0.24	-0.53	-0.18	-0.23	0.06	0.06
**Italy North**	-0.48	-0.36	-0.35	-0.04	-0.33	-0.19	-0.02	-0.14	-0.39	-0.34	-0.30	-0.45
**Italy Central**	-0.35	-0.55	-0.36	-0.10	-0.30	-0.13	-0.06	0.00	-0.39	-0.18	-0.24	-0.44
**Italy South**	-0.15	-0.27	-0.18	0.11	-0.16	-0.11	-0.05	0.20	-0.07	-0.05	0.00	-0.04

Country data from Table S1 (1950-2019)

**France**	-0.07	0.04	-0.36	-0.16	-0.37	-0.45	-0.35	-0.45	-0.33	-0.44	-0.22	-0.10
**Germany**	0.38	0.24	0.16	0.19	0.07	-0.19	-0.27	-0.52	-0.20	-0.09	0.05	0.25
**Italy**	-0.48	-0.56	-0.44	-0.10	-0.33	-0.25	-0.14	-0.20	-0.48	-0.36	-0.36	-0.41

9.2 AO vs. Precipitation (Table S11)

Grid data (1950-2018)

	Jan	Feb	Mar	Apr	May	Jun	Jul	Aug	Sept	Oct	Nov	Dec
**France North**	-0.02	-0.02	-0.22	-0.17	-0.05	-0.47	-0.39	-0.41	-0.36	-0.32	-0.08	-0.08
**France South**	-0.30	-0.18	-0.37	-0.20	-0.16	-0.31	-0.10	-0.34	-0.24	-0.35	-0.21	-0.30
**Germany North**	0.22	0.16	0.08	-0.05	-0.05	-0.14	-0.29	-0.36	-0.32	-0.11	0.10	0.14
**Germany South**	-0.18	-0.17	-0.16	-0.11	0.10	-0.33	-0.10	-0.36	-0.28	-0.28	-0.10	-0.13
**Italy North**	-0.45	-0.34	-0.32	-0.22	-0.10	0.03	0.18	-0.02	-0.36	-0.24	-0.15	-0.41
**Italy Central**	-0.33	-0.45	-0.27	0.01	-0.27	0.14	0.16	-0.01	-0.30	-0.14	-0.18	-0.35
**Italy South**	-0.01	0.01	-0.09	0.09	-0.09	0.10	0.15	0.16	0.04	-0.04	0.04	-0.04

Country data from Table S2 (1950-2019)

**France**	-0.30	-0.13	-0.41	-0.24	-0.15	-0.45	-0.26	-0.42	-0.37	-0.43	-0.28	-0.29
**Germany**	0.07	-0.02	-0.05	-0.09	0.02	-0.26	-0.19	-0.44	-0.39	-0.25	-0.04	-0.05
**Italy**	-0.43	-0.43	-0.34	-0.10	-0.27	0.01	0.09	-0.05	-0.39	-0.32	-0.26	-0.31

9.3 NCP vs. Precipitation (Table S12)

Grid data (1950-2018)

	Jan	Feb	Mar	Apr	May	Jun	Jul	Aug	Sept	Oct	Nov	Dec
**France North**	-0.64	-0.69	-0.71	-0.63	-0.30	-0.42	-0.29	-0.52	-0.63	-0.73	-0.74	-0.65
**France South**	-0.42	-0.49	-0.43	-0.16	0.00	-0.01	0.07	0.10	-0.05	-0.18	-0.10	-0.39
**Germany North**	-0.44	-0.49	-0.50	-0.59	-0.65	-0.56	-0.70	-0.57	-0.68	-0.71	-0.64	-0.43
**Germany South**	-0.59	-0.61	-0.49	-0.58	-0.20	-0.28	-0.19	-0.24	-0.60	-0.67	-0.56	-0.56
**Italy North**	-0.31	-0.55	-0.41	-0.11	-0.08	0.10	0.08	0.28	-0.11	-0.24	-0.35	-0.41
**Italy Central**	-0.24	-0.26	-0.37	0.21	0.04	0.30	0.35	0.42	0.01	-0.14	-0.20	-0.22
**Italy South**	0.23	0.25	0.08	0.09	0.08	0.24	0.26	0.22	0.29	0.26	0.34	0.10

Country data from Table S3 (1950-2019)

**France**	-0.70	-0.77	-0.70	-0.51	-0.20	-0.27	-0.16	-0.27	-0.35	-0.59	-0.62	-0.74
**Germany**	-0.53	-0.57	-0.57	-0.62	-0.57	-0.53	-0.57	-0.40	-0.72	-0.74	-0.66	-0.54
**Italy**	-0.30	-0.47	-0.44	-0.09	-0.09	0.12	0.09	0.30	-0.06	-0.20	-0.27	-0.33

9.4 MOI2 vs. Precipitation (Table S13)

Grid data (1950-2018)

	Jan	Feb	Mar	Apr	May	Jun	Jul	Aug	Sept	Oct	Nov	Dec
**France North**	0.29	0.09	0.00	0.19	0.04	0.01	-0.03	-0.18	0.07	-0.03	0.16	0.35
**France South**	-0.42	-0.35	-0.41	0.00	-0.12	0.14	-0.03	-0.17	0.05	-0.61	-0.40	-0.38
**Germany North**	0.61	0.29	0.24	0.40	0.36	-0.05	0.01	0.22	0.05	0.38	0.36	0.56
**Germany South**	0.32	0.10	0.09	0.28	0.09	0.01	-0.19	0.01	-0.04	0.10	0.26	0.29
**Italy North**	-0.50	-0.53	-0.36	0.05	-0.05	-0.19	-0.28	-0.21	-0.28	-0.29	-0.30	-0.41
**Italy Central**	-0.30	-0.39	-0.30	-0.05	-0.15	-0.28	-0.35	-0.32	-0.38	-0.13	-0.12	-0.46
**Italy South**	-0.15	-0.03	-0.09	0.35	-0.11	-0.11	-0.30	-0.12	-0.13	-0.19	-0.06	-0.12

Country data from Table S4 (1950-2019)

**France**	-0.13	-0.19	-0.23	0.06	0.04	0.06	-0.01	-0.14	0.03	-0.38	-0.17	0.00
**Germany**	0.56	0.22	0.19	0.41	0.30	-0.05	-0.10	0.12	-0.01	0.28	0.30	0.49
**Italy**	-0.47	-0.57	-0.42	0.09	-0.17	-0.20	-0.37	-0.31	-0.38	-0.37	-0.28	-0.46

9.5 WeMOI vs. Precipitation (Table S14)

Grid data (1950-2018)

	Jan	Feb	Mar	Apr	May	Jun	Jul	Aug	Sept	Oct	Nov	Dec
**France North**	0.64	0.66	0.64	0.57	0.15	0.38	0.23	0.46	0.59	0.49	0.68	0.72
**France South**	0.08	0.27	0.13	0.17	0.14	0.30	0.12	0.42	0.17	-0.08	-0.07	0.32
**Germany North**	0.67	0.52	0.54	0.63	0.44	0.20	0.24	0.45	0.42	0.57	0.65	0.55
**Germany South**	0.70	0.66	0.53	0.53	0.25	0.53	0.26	0.42	0.53	0.58	0.61	0.64
**Italy North**	0.08	0.18	0.25	0.49	0.26	0.11	0.09	0.06	0.15	0.31	0.16	0.31
**Italy Central**	0.22	0.12	0.19	0.21	0.18	-0.19	0.00	0.07	0.00	0.26	0.21	0.18
**Italy South**	-0.08	-0.21	-0.09	0.23	0.00	-0.11	-0.12	-0.01	-0.20	-0.21	-0.26	-0.06

Country data from Table S5 (1950-2019)

**France**	0.43	0.61	0.51	0.42	0.19	0.45	0.28	0.61	0.45	0.26	0.44	0.72
**Germany**	0.77	0.63	0.61	0.66	0.39	0.44	0.35	0.49	0.50	0.67	0.68	0.68
**Italy**	0.17	0.18	0.26	0.48	0.21	0.19	0.14	0.15	0.10	0.28	0.24	0.24

10. References

Fox, J. (3rd ed. 2016). *Applied Regression and Generalized Linear Models*. Thousand Oaks, CA: Sage.

Kantelhardt, J.W., Koscielny-Bunde, E., Rego, H.H.A., Havlin, S., Bunde, A. (2001). Detecting long-range correlations with detrended fluctuation analysis. *Physica A: Statistical Mechanics and its Applications*, 295(3), 441-454.

Kutiel, H., and Benaroch, Y. (2002). North Sea-Caspian Pattern (NCP) – an upper level atmospheric teleconnection affecting the Eastern Mediterranean: Identification and definition: *Theoretical and Applied Climatology*, 71(1), 17-28.

Matalas, N.C. (1963). Autocorrelation of rainfall and streamflow minimums. *Geological Survey Professional Paper 434-B*, U.S. Department of the Interior.
